# Synthesis of cholera toxin B subunit glycoconjugates using site-specific orthogonal oxime and sortase ligation reactions

**DOI:** 10.3389/fchem.2022.958272

**Published:** 2022-09-14

**Authors:** Jonathan P. Dolan, Darren C. Machin, Simone Dedola, Robert A. Field, Michael E. Webb, W. Bruce Turnbull

**Affiliations:** ^1^ School of Chemistry and Astbury Centre of Structural Biology, University of Leeds, Leeds, United Kingdom; ^2^ Iceni Glycoscience Ltd., Norwich, United Kingdom; ^3^ Department of Chemistry and Manchester Institute of Biotechnology, University of Manchester, Manchester, United Kingdom

**Keywords:** sortase, glycopeptide, glycoconjugates, oxime ligation, protein modification, transpeptidase, neoglycoproteins

## Abstract

The chemoenzymatic synthesis of a series of dual N- and C-terminal–functionalized cholera toxin B subunit (CTB) glycoconjugates is described. Mucin 1 peptides bearing different levels of Tn antigen glycosylation [MUC1(Tn)] were prepared *via* solid-phase peptide synthesis. Using sortase-mediated ligation, the MUC1(Tn) epitopes were conjugated to the C-terminus of CTB in a well-defined manner allowing for high-density display of the MUC1(Tn) epitopes. This work explores the challenges of using sortase-mediated ligation in combination with glycopeptides and the practical considerations to obtain high levels of conjugation. Furthermore, we describe methods to combine two orthogonal labeling methodologies, oxime- and sortase-mediated ligation, to expand the biochemical toolkit and produce dual N- and C-terminal–labeled conjugates.

## Introduction

The development of a methodology for the site-specific conjugation of glycopeptides and carbohydrate epitopes to multimeric scaffolds is important because of their potential as vaccines, therapeutics, or in glycan/glycopeptide arrays (Doores et al., 2006; Godula and Bertozzi, 2012; Branson et al., 2014; Ulrich et al., 2014; Pifferi et al., 2020). In many cases it is advantageous to be able to attach more than one distinct group to a scaffold in a controlled manner, which requires the use of orthogonal ligation strategies; a variety of approaches for protein ligations, such as cysteine derivatization (Dadová et al., 2018; Tessier et al., 2019), azide–alkyne cycloaddition (Wang et al., 2003; Agard et al., 2004; Bruins et al., 2018), and oxime ligation (Geoghegan and Stroh, 1992; Chen et al., 2003) have been combined to enable orthogonal labeling (Smith et al., 2009; Yi et al., 2011; Rashidian et al., 2013; Matos et al., 2020). Another popular ligation strategy employs transpeptidases (Harmand et al., 2018; Fottner et al., 2021; Zhang et al., 2021); for example, the sortases that are responsible for reversibly catalyzing the attachment of virulence factors to the cell walls of Gram-positive bacteria (Mazmanian et al., 1999; Popp et al., 2007). Sortase A (SrtA) from *Staphylococcus aureus* ligates proteins carrying an LPXTGX recognition motif to substrates carrying an N-terminal glycine residue (Ton-That et al., 2000; Spirig et al., 2011). This class of reactions has been extensively optimized both by engineering the substrate and the enzymes involved (Morgan et al., 2022). In particular, directed evolution has yielded sortases with enhanced activity (Chen et al., 2011, 2016; Hirakawa et al., 2012), perhaps most notably Sortase 7M which exhibits enhanced kinetics and Ca^2+^-independent activity (Wuethrich et al., 2014). Sortase ligation has been used for the cyclisation of glycopeptides (Wu et al., 2011), conjugation of two mucin glycopeptides (Matsushita et al., 2009), synthesis of GPI analogues (Guo et al., 2009), and generation of glycoform-defined VHH–MUC1 vaccine constructs (Fang et al., 2017), but we are not aware of reports of sortase-mediated ligation of glycopeptides to multimeric proteins to generate multivalent neoglycoproteins.

Here, we report synthetic studies for the conjugation of MUC1 glycopeptides to the cholera toxin B subunit (CTB). Mucin 1 (MUC1) is an extensively *O-*glycosylated protein whose extracellular domain extends up to 200–500 nm beyond the surface of the cell, with a total molecular weight of 250–500 kDa (Brayman et al., 2004; Hattrup and Gendler, 2008; Nath and Mukherjee, 2014). The extracellular domain comprises a highly conserved variable number of tandem repeats (VNTR) of 20 amino acids, which is repeated between 20–125 times depending on the individual (Baldus et al., 2004; Hattrup and Gendler, 2008). This VNTR domain is rich in serine and threonine permitting heavy *O*-glycosylation and proline, which contributes to the rod-like structure of the extracellular domain (Baldus et al., 2004; Hollingsworth and Swanson, 2004). Dramatic changes to MUC1 glycosylation are associated with various carcinomas (Gendler, 2001; Awaya et al., 2004; Singh et al., 2006; Krishn et al., 2016). Abnormal truncated *O*-linked glycans are recognized as potential cancer markers and result in more of the peptide backbone being exposed to the environment (Burchell et al., 2001; Munkley, 2016). The Tn (core) antigen is the simplest of the tumor associated carbohydrate antigens comprising of α-*O*-GalNAc linked to either serine or threonine.

We report methods to attach MUC1 glycopeptides to the cholera toxin B subunit (CTB). The natural role of this pentameric glycan-binding protein is to deliver a toxic ADP-ribosyltransferase enzyme into intestinal cells (Sánchez and Holmgren, 2008). However, the non-toxic CTB subunit is also widely used as a neuronal cell tracer (Vercelli et al., 2000; Saleeba et al., 2019), and it has also been used as a scaffold for the preparation of multivalent neoglycoprotein inhibitors of bacterial toxin adhesion and as a mucosal adjuvant (Holmgren et al., 1993, 2005; Pizza et al., 2001; Branson et al., 2014). It is our interest in these applications of the CTB protein that has led us to develop a range of ligation methods for the site-specific modification of this protein at its N-terminus and also through site-specific incorporation of azidohomoalanine for bioorthogonal ligation reactions (Branson et al., 2014; Machin et al., 2019; Haigh et al., 2020; Balmforth et al., 2021). The work reported here extends our toolbox of methods to C-terminal modification of CTB by exploring the feasibility of using sortase to conjugate naturally occurring glycopeptide epitopes to produce a series of well-defined glycoconjugates. Furthermore, we also demonstrate the feasibility of orthogonal modification combining sortase and oxime ligation to add additional functionality to the N-terminus. The ability to expand the functionality of site-specific glycoconjugates for immobilization, add additional carbohydrate epitopes, or to increase immunogenicity is key to developing relevant and useful therapeutics or increasing the chances of detecting glycan-binding proteins.

## Materials and methods

### General procedure for solid-phase glycopeptide synthesis

Low loading Rink Amide MBHA resin (loading: 0.33–0.35 mmol/g, 100 µmol) was shaken in DMF (5 ml) for 2 h, filtered, and washed with DMF. Fmoc deprotection: resin-bound peptide intermediates were shaken in 20% piperidine in DMF (3 × 5 ml × 3 min) and washed with DMF, CH_2_Cl_2_, and DMF. Couplings: the resin was treated with a 5-fold excess of Fmoc-amino acid (except for the Fmoc-Ser/Thr (GalNAc)-OH which was employed in a 2-fold excess) in 2 ml DMF containing HCTU (5 equiv) and DIPEA (10 equiv). The mixture was shaken and coupling times were 40 min (except for the Fmoc-Ser/Thr (GalNAc)-OH and the subsequent coupling each of which were 12 h in duration). The excess reagents removed by filtration, and the resin was washed with DMF, CH_2_Cl_2_ and DMF. *O*-acetyl deprotection: following coupling of the final amino acid, resin was washed with methanol, shaken in 70% hydrazine hydrate in methanol (3 × 5 ml × 5 min), and washed with methanol. Glycopeptide cleavage: following Fmoc and O-acetyl deprotection, the resin was washed with CH_2_Cl_2_ and methanol, before drying under vacuum. The resin was suspended in TFA:H_2_O:TIS (95:2.5:2.5, 5 ml) and shaken for a maximum of 2 h. The mixture was filtered and washed with TFA. The solution was concentrated under a stream of N_2_ to <1 ml. Glycopeptide obtained by ether precipitation, isolated by centrifugation and pellet further washed with ether. The white precipitate was dissolved in H_2_O and lyophilized to give white foam. The yield and characterization data of all synthesized peptides/glycopeptides can be found in Supplementary Material.

### Determination of sortase-mediated ligation efficiency

For a total volume of 100 µl, CTB–LPETGA (200 µM) and appropriate peptide/glycopeptide (3 mM, 15 equiv) were mixed, and the reaction mixture was made up to volume with the HEPES buffer {+DMSO [10% *v*/*v* (final)], if required} before the addition of Sortase 7M (30 µM, 15 mol%). The reaction mixture was incubated at 25°C, and timepoints for analysis by SDS-PAGE were taken at 2, 5, 10, 20, 30, 60, 90, and 120 min. For time point samples, 5 µl of the reaction was diluted in 15 µl H_2_O and mixed with 5 × SDS loading buffer (5 µl). Samples were heated to 95°C for 5 min before being frozen and stored at −20°C before analysis by SDS-PAGE. Samples were run on a 15% Tris-glycine SDS-PAGE gel, stained with Coomassie blue (12 h), destained, and imaged using a Bio-Rad Gel Doc XR system. The relative quantity of labeled product and unlabeled starting material was quantified by densitometry (Bio-Rad Image Lab 6.0.1, Bio-Rad Laboratories Ltd.). Data were analyzed in OriginPro 2019b (OriginLab Corporation, Northampton, MA, United States). Original SDS-PAGE gels are included within the Supplementary Material.

### General procedure for preparative scale sortase ligation using CBD-Sortase 7M

For a total volume of 200 µl, the protein to be labeled (200 µM) and peptide/glycopeptide (3,000 µM, 15 eq.) were mixed, and the reaction mixture was made up to volume with the appropriate buffer before the addition of His_6_-CBD-Srt7M (30 µM, 15 mol%). The reaction mixture was incubated for 2 h at 25°C. Once the reaction has reached completion by mass spectrometry, 200 µl of chitin resin [50% slurry in TBS (pH 7.2)] was added, and the mixture incubated on ice for 15 min. The solution was filtered using a centrifugal cellulose acetate filter (4,000 *× g*, 1 min). The excess substrate was removed by either diafiltration, dialysis, or size exclusion chromatography [Superdex 75 10/300, Tris-buffered saline (pH 7.2)].

### General procedure for Thr/Ser oxidation and oxime ligation

Protein to be labeled was buffer exchanged into sodium phosphate buffer (pH 7.4); it is very important that the buffer does not contain potassium. Performing reactions in the presence of K^+^ ions will result in oxidation failing to reach completion (Brabham et al., 2020). Oxidation: the protein to be labeled (500 µl, 200 µM) in sodium phosphate buffer was mixed with L-methionine (4 µl of 250 mM stock, final concentration 2.0 mM, 10 eq) and NaIO4 (2 µl of 250 mM stock, final concentration 1.0 mM, 5 eq). The reaction was followed by ES-MS, and it typically completed within 10 min. The protein was purified using a G-25 minitrap desalting column equilibrated and eluted with sodium phosphate buffer (pH 6.8). Oxime ligation: to the oxidized protein obtained from the G-25 minitrap desalting column (1 ml, ∼100 µM) was added aniline (18 µl, final concentration 200 mM, 1,000 equiv.) and the oxyamine substrate (8 µl of 250 mM stock in DMSO, final concentration 2.0 mM, 10 equiv.). The mixture was briefly mixed by vortexing and incubated at 37°C for 8–16 h. The oxime-labeled protein was purified using a PD-10 desalting column equilibrated and eluted with sodium phosphate buffer (pH 6.8). The reactions were followed by protein HRMS, which are included within the Supplementary Material.

## Results and discussion

### Preparation of glycopeptide and protein substrates

**FIGURE 1:**
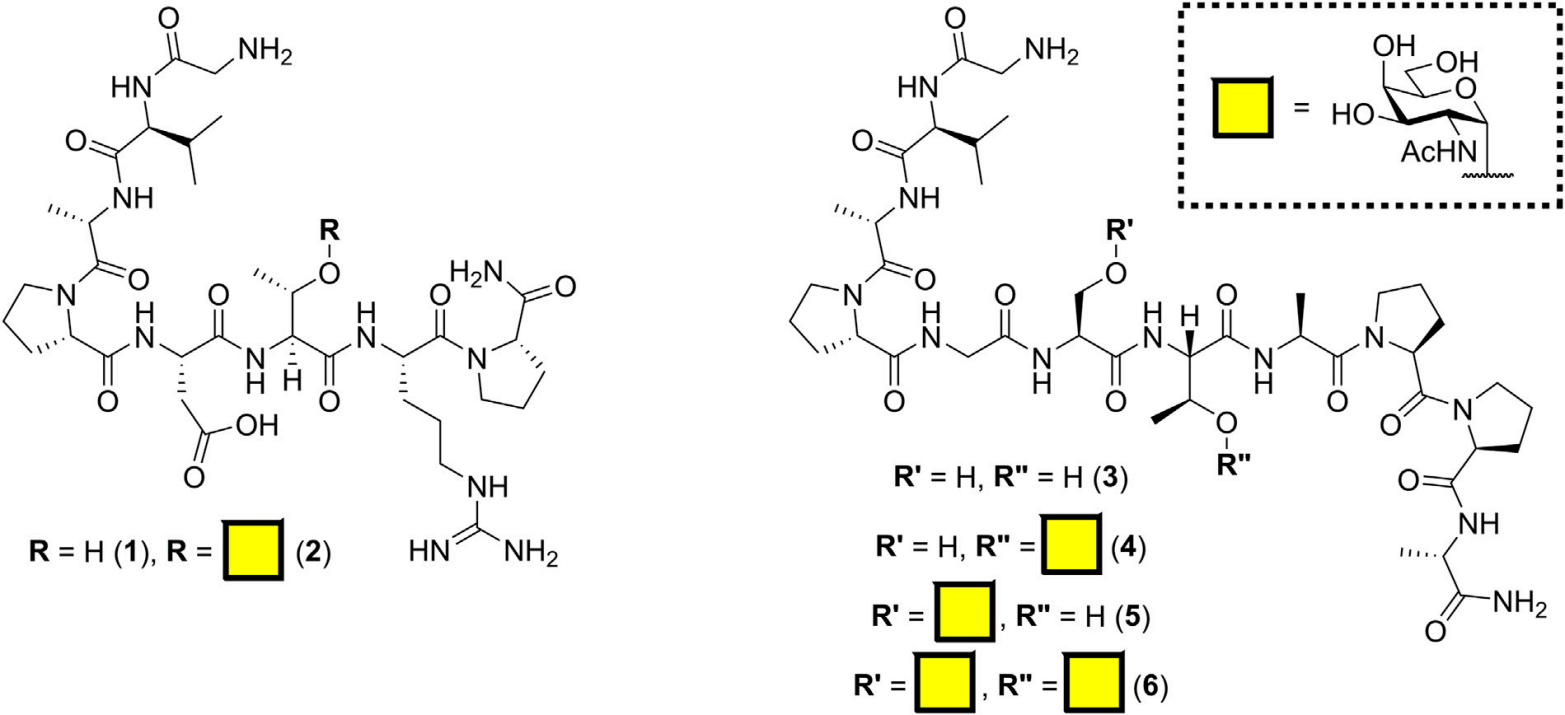
Structure of MUC1 (Tn) epitopes based on SM3 (**1–2**) and H-2D^b^ (**3–6**) binding domains.

The antibody SM3 (identified from the sera of breast cancer patients) and the mouse class I MHC product H-2D^b^ are known to bind regions of incompletely glycosylated MUC1, either APDTRP or APGSTAPPA, respectively (Apostolopoulos et al., 1998; Dokurno et al., 1998; Burchell et al., 2001; Martínez-Sáez et al., 2015). Previously, Fang et al. (2017) reported the C-terminal sortase conjugation of αMUC1(Tn) glycopeptide [GGGCTSAPDT (GalNAc)RPAP] to camelid-derived heavy chain–only antibody fragments (VHHs) using Sortase 5M. Our initial aim was to keep the linker length to a minimum; therefore, the SM3 and H-2D^b^ sequences were synthesized by solid-phase peptide synthesis with a Gly–Val N-terminal tag for sortase-mediated ligation. Inclusion of valine provided both a minimal length spacer and a unique signal enabling quantification of peptide concentration by NMR spectroscopy. Peracetylated α-O-GalNAc-Ser/Thr amino acids, synthesized from D-galactosamine as outlined in Supplementary Scheme S1 (Xu et al., 2004; De Silva et al., 2009), were incorporated into the peptides to create all three potential GalNAc (Tn) glycosylation patterns (Figure 1). To minimize off-resin manipulation of the glycopeptides, *O*-acetyl protecting groups were removed on resin using 70% hydrazine hydrate in methanol. This deacetylation reaction reached completion within 15 min rather than 2 h as previously reported (Mitchell et al., 2001). Glycopeptides were cleaved from the resin using standard TFA cleavage conditions; no acid-catalyzed cleavage of GalNAc moiety was observed, provided cleavage was not allowed to proceed for more than 2 h.

Cholera toxin B subunit (CTB) was chosen as the model pentameric scaffold protein for glycopeptide conjugation. A synthetic gene for cholera toxin B subunit harboring a C-terminal NGGNLPETGA extension including a sortase recognition motif (CTB–LPETGA) was constructed by assembly PCR. The protein was expressed in *E. coli* and isolated from the growth media by ammonium sulfate precipitation, followed by nickel affinity and size exclusion chromatography steps. Characterization of CTB–LPETGA by SDS-PAGE and HRMS revealed a truncation present in approximately 10% of the CTB protomer units corresponding to 11 amino acids from the C-terminus being absent including the LPETGA sortase motif (Supplementary Figure S1). The cause of the truncation could potentially have been premature termination of translation or subsequent proteolysis; however, addition of protease inhibitors to the growth media (both prior to induction and ammonium sulfate precipitation) or to the buffer used to resuspend the ammonium sulfate precipitate did not have any effect. Having 10% of peptide truncation means that on average there are 4.5 sortase sites available for ligation per pentamer, rather than the expected 5, but this is unlikely to have any negative impact on any downstream application of the ligation methods reported here.

### Effect of DMSO and Pro4 on ligation efficiency

**FIGURE 2:**
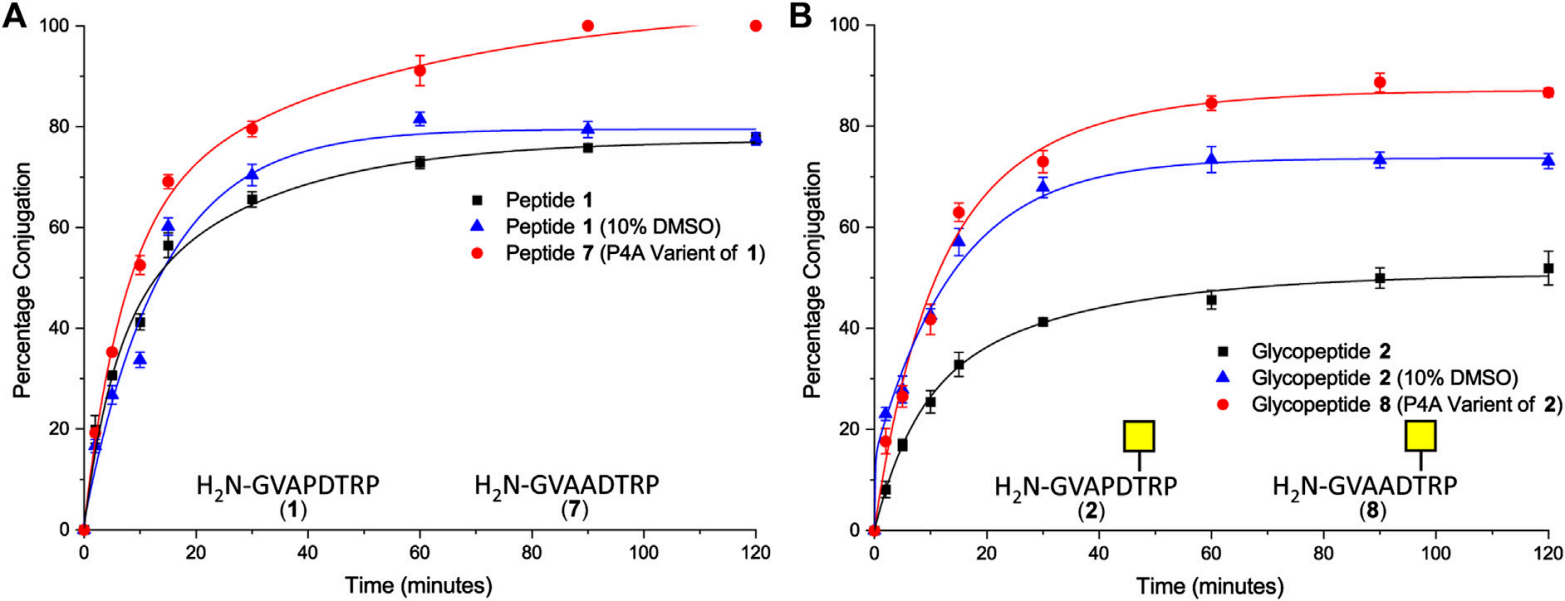
**(A)** Time-course of Sortase 7M–mediated ligation of CTB–LPETGA and peptide **1** based on the SM3 recognition sequence or peptide **7** (P4A variant of peptide **1**). **(B)** Time-course of Sortase 7M–mediated ligation of CTB–LPETGA and glycopeptide **2** based on the SM3 recognition sequence or glycopeptide **8** (P4A variant of glycopeptide **2**). Percentage conjugation an average of three densitometry measurements and error bars indicate 2 × standard deviation.

Peptide **1** and glycopeptide **2**, based on the SM3 sequence, were ligated to CTB–LPETGA using Sortase 7M (Wuethrich et al., 2014), and the reactions were followed by SDS-PAGE and quantified by densitometry. After 2 h conjugation with the SM3 peptide **1** had reached its maximum conversion of 78% (Figure 2A). Conjugation with the SM3 glycopeptide **2** reached a maximum conversion of 52%, approximately 25 percentage points lower than observed for the non-glycosylated peptide under the same conditions (Figure 2B). Considering that the GalNAc residue is attached to Thr-6 of the peptide substrate, it seemed unlikely that glycosylation was having a detrimental steric effect on the ligation reaction. We therefore postulated that the sugar residue might cause some conformational change in the peptide that reduced ligation efficiency. Therefore, each reaction was repeated in the presence of 10% DMSO to act as chaotropic agent and disrupt any putative hydrogen bonding within the peptide/glycopeptide (Jackson and Mantsch, 1991). No change in conjugation efficiency was observed for peptide **1** (Figure 2A); however, a significant increase in the level of conjugation was observed for glycopeptide **2**. After 2 h, 73% of the available sortase sites were converted to the product, which was an increase approximately 20% compared to the reaction without DMSO present (Figure 2B). The rate and total level of conjugation for peptide **1** and glycopeptide **2** were comparable in the presence of 10% DMSO. No significant reduction in rate of labeling was observed, indicating that this concentration of DMSO was sufficient to interrupt any unfavorable intramolecular interactions within the glycopeptide, but insufficient to impair Sortase 7M activity, and unlikely to perturb the highly stable CTB structure (Goins and Friere, 1988).

Next, the effect of the peptide sequence on conjugation efficiency was evaluated. The 20 amino acid MUC1 tandem repeat domain is known to be highly kinked, which in part is induced by its five proline residues (Brayman et al., 2004). The second set of experiments focused on the N-terminal motif of GVAPXS/T [where X is D (SM3) or G (H-2D^b^)] to evaluate if the proline residue closest to the nucleophilic glycine residue could be the cause of the poor conjugation efficiency for the glycopeptide. Variants of the SM3 peptide and glycopeptide substrates were synthesized in which Pro4 was changed to Ala and ligation was repeated in the absence of DMSO. After 1.5 h, quantitative ligation was achieved for the P4A peptide **7** (Figure 2A) and conjugation of P4A glycopeptide **8** reached a maximum level of 87% (Figure 2B). Ligation of both P4A substrates were a significant improvement over the natural substrates and confirmed the Pro4 had an influence on conjugation efficiency of both the peptide and glycopeptide substrates. It is possible that ligation of glycopeptide **8** could have improved further if the reaction were spiked with 10% DMSO; however, as this was not the native SM3 epitope, further experiments to confirm this hypothesis were not performed.

### Effect of N-terminal flexibility on glycopeptide ligation efficiency

**FIGURE 3:**
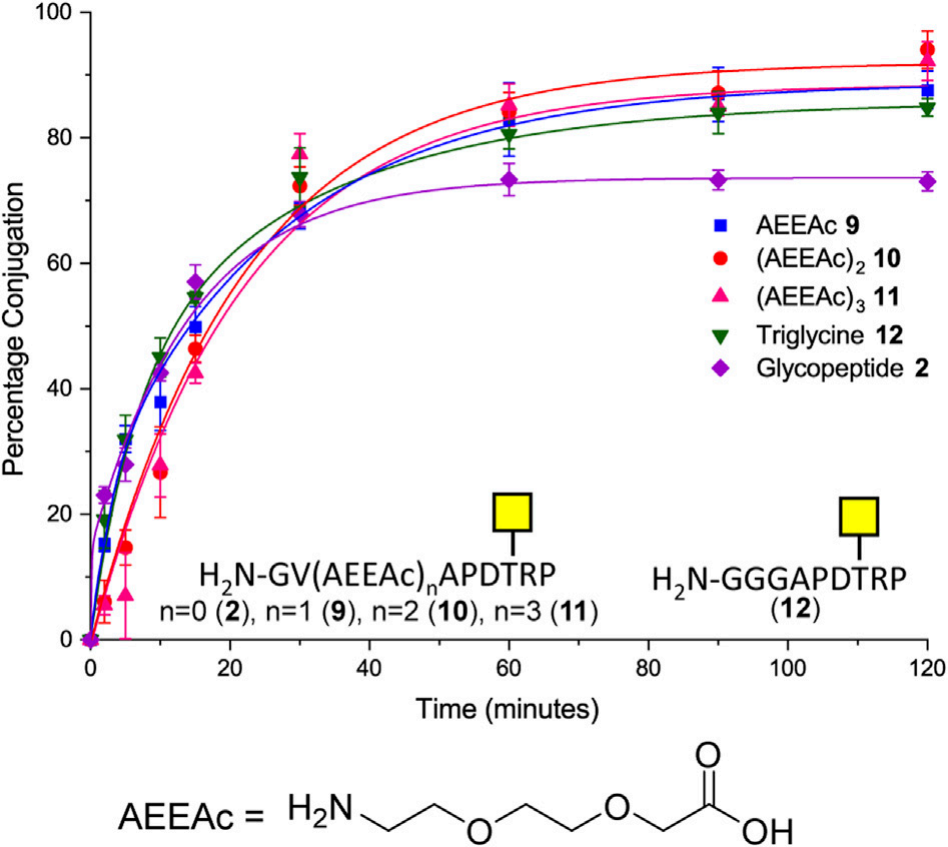
Time-course of Sortase 7M–mediated ligation of CTB–LPETGA and glycopeptide **2** and **9**–**12** based on the SM3 recognition sequence. The reaction performed in the presence of 10% DMSO. AEEAc, 8-amino-3,6-dioxaoctanoic acid. Percentage conjugation an average of three densitometry measurements and error bars indicate 2 × standard deviation.

Although removal of the proline residue resulted in near quantitative ligation, it is unclear if the proline residue may induce important secondary structure within the glycopeptide. It was considered important not to perturb the conformation of the (glyco) peptides if the methodology were to be used to develop glycoconjugates, which could be used as therapeutics or detection of novel carbohydrate binding proteins. Therefore, the effect of introducing various length linkers between the sortase handle and the MUC1 sequence was investigated to determine if conjugation efficiency could be improved whilst maintaining amino acids that could be important for secondary structure. One, two, or three copies of 8-amino-3,6-dioxaoctanoic acid (AEEAc-OH), each equivalent to insertion of PEG_3_, were introduced between the GV sortase handle and the SM3 sequence. Furthermore, the effect of increasing N-terminal flexibility was investigated by changing the Gly–Val sortase handle to a triglycine motif (**12**) in analogy to the longer [GGGCTSAPDT (GalNAc)RPAP] sequence previously investigated (Fang et al., 2017). All four extended peptide sequences (**9**, **10**, **11**, **12**) gave statistically significant improvements in conjugation (12–21 percentage points) relative to glycopeptide **2** under the same conditions (Figure 3). In the case of compound **12**, the improvement in conjugation is likely due to the increased flexibility of the N-terminus rather than the additional length provided by the addition of a single amino acid (GGG vs. GV) (Figure 3). Only peptide **10** with the PEG_6_ equivalent linker showed a significant improvement in the level of conjugation relative to the glycopeptide bearing the N-terminal triglycine (**12**).

**FIGURE 4:**
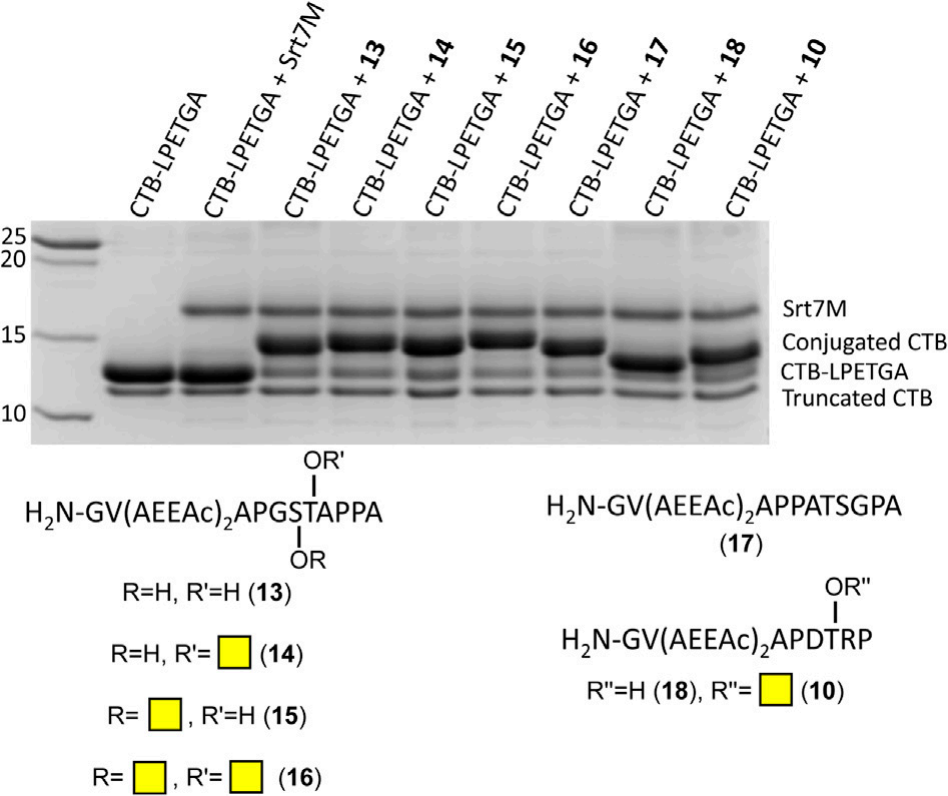
SDS-PAGE gel showing sortase ligation after 2 h using peptides (**13**, **17**, and **18**) and glycopeptides (**10**, **14**–**16**) containing a double length AEEAc linker, equivalent to PEG_6_. Levels of conjugation listed in Table 1.

As the double AEEAc linker showed the highest levels of conjugation in the preliminary experiments, the peptides and glycopeptides based on the SM3 (APDTRP) and H-2D^b^ (APGSTAPPA) motifs were resynthesized with the double AEEAc linker (*n* = 2). After 2 h the level of conjugation between CTB–LPETGA and the new peptides and glycopeptides containing a PEG_6_ equivalent linker (**13**–**18**) in the presence of 10% DMSO was between 80%–85% (by SDS-PAGE and densitometry) (Figure 4; Table 1). Compared to the original glycosylated SM3 substrate **2** in the absence of DMSO, the optimized linker length and conditions for glycopeptide **10** resulted in an increased conversion of 32 ± 4 percentage points.

**TABLE 1:** Percentage of conjugated and unlabeled sortase sites on CTB–LPETGA after 2 h.

Substrate	Conjugated (%)^a^
13	82 (±3)
14	80 (±2)
15	80 (±2)
16	81 (±2)
17	85 (±1)
18	85 (±2)
10	84 (±3)

a Densitometry performed by comparing intensity of unlabeled (CTB–LPETGA) and conjugated CTB bands. CTB without the LPETGA tag (CTB) accounts for ∼10% of each sample. Average of three technical repeats of the densitometry measurements. Errors indicate 2 × standard deviation.

### Development and testing of chitin-binding Sortase 7M variant

The ability to remove the sortase enzyme rapidly from the preparative scale ligation reactions efficiently would be vital as prolonged exposure to sortase will eventually lead to hydrolysis of the ligated product (Morgan et al., 2022). The standard methodology for removing sortase from ligation reactions by exploiting the affinity of its His-tag for Ni-NTA resin was not possible because of the natural affinity of CTB for Ni-NTA resin (Dertzbaugh and Cox, 1998). Attempts to exploit the difference in affinity of these two proteins for the Ni-NTA resin were unsuccessful, and further attempts to isolate the ligation product by size exclusion chromatography (SEC) led to increased levels of hydrolysis compared to the crude sample prior to being loaded on the SEC column. Although immobilization of sortase was a potential solution (Steinhagen et al., 2013; Witte et al., 2015), once immobilized the activity of the enzyme is reduced. As a result, we decided to investigate an alternative fusion construct of Sortase 7M which could be used in solution for the reaction and then selectively removed at the end of the reaction. An N-terminal chitin-binding domain (CBD) was selected because of its effectively irreversible binding to chitin resin and its small size which should not interfere with activity of the enzyme.

**FIGURE 5:**
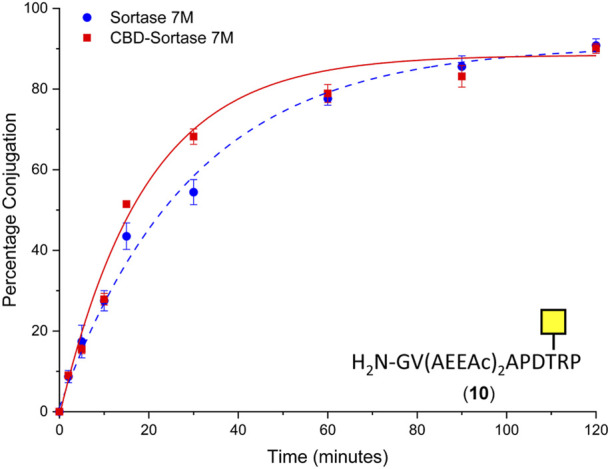
Time-course for ligation of CTB–LPETGA and glycopeptide **10** using Sortase 7M (■) and chitin-binding domain (CBD) Sortase 7M (•). Percentage conjugation an average of three densitometry measurements and error bars indicate 2 × standard deviation.

**FIGURE 6:**
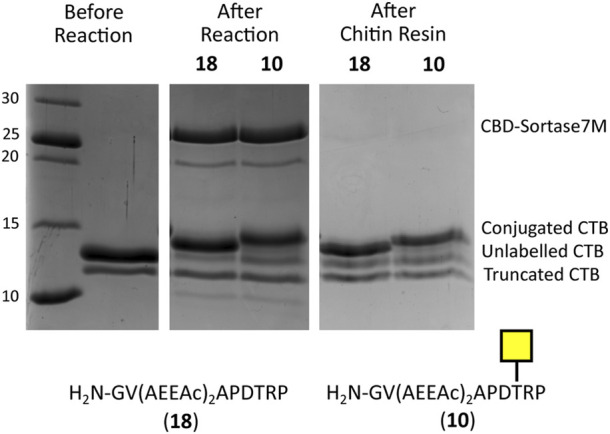
SDS-PAGE gel demonstrating removal of CBD-Sortase 7M from reaction mixture using 1.5 eq of chitin resin. No change in the level of conjugation is observed before and after removal of CBD-Sortase 7M.

To ensure the CBD domain did not interfere with the transpeptidase activity, the conjugation of CTB to the SM3 glycopeptide with the double length AEEAc linker (**10**) was repeated using both the Sortase 7M and CBD-Sortase 7M variant. Although the chitin-binding variant of Sortase 7M was found to ligate slightly faster over the first 30 min compared to the non–chitin-binding variant, at timepoints beyond 1 h the difference in conjugation between the two enzymes was negligible with conjugation reaching 90% for both enzymes after 2 h (Figure 5). The efficiency of removing CBD-Sortase 7M from ligation reactions was evaluated by repeating the experiments with SM3 peptide **18** and glycopeptide **10**. After 2 h reaction in the presence of CBD-Sortase 7M, chitin resin was added and the mixture was incubated on ice to minimize any further reaction past the deemed endpoint prior to removal of the resin by centrifugal filtration. An analysis of samples before and after use of the chitin resin showed complete removal of the CBD-Sortase 7M, and no significant difference in the level of conjugation before and after removal of chitin resin was observed (Figure 6).

### Combining orthogonal N- and C-terminal ligation methodologies

Orthogonal modification of the C- and N-terminus of a protein scaffold allows the opportunity to introduce more than one new functionality at specific sites on a protein scaffold. Oxime ligation at the N-terminus of CTB was first described for the construction of well-defined CTB-viral peptide immunogens (Chen et al., 2003). We have previously applied oxime ligation of CTB for the preparation of multivalent neoglycoprotein bacterial toxin inhibitors and for N-terminal biotinylation of CTB for phage display (Branson et al., 2014; Balmforth et al., 2021). To explore if oxime and C-terminal sortase-mediated ligation could be used orthogonally, the N-terminus of CTB–LPETGA was first biotinylated before the peptides and glycopeptides (**10**, **13**–**18**) were conjugation to the C-terminus using CBD-Sortase 7M. We chose to perform the ligation reactions in this order as periodate oxidation of a glycoprotein bearing terminal GalNAc residues risks oxidation of the sugar residues. An alternative strategy of N-terminal periodate oxidation followed by sortase ligation to attach the glycopeptides and then oxime ligation to introduce the biotin groups would not be practical as delays to the oxime step result in cyclisation of the N-terminal aldehyde group onto the peptide backbone (Rose et al., 1999).

**FIGURE 7:**
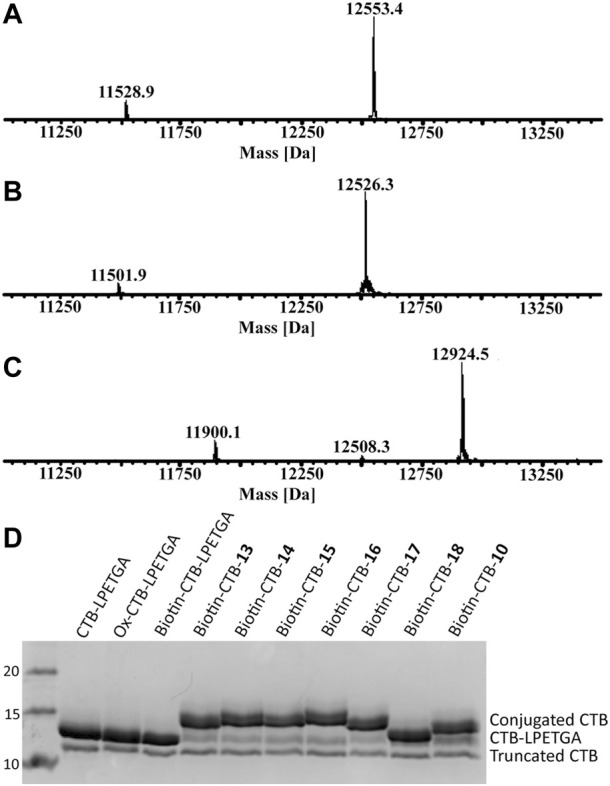
**(A)** ESI-MS of CTB–LPETGA (12,553.4 Da) and truncated CTB (11,528.9 Da) before treatment with NaIO_4_. **(B)** ESI-MS of CTB–LPETGA following oxidation with periodate. **(C)** ESI-MS following treatment of oxidised-CTB with alkoxyamine-PEG_4_-biotin and aniline, showing quantitative N-terminal biotinylation of both substrates. **(D)** SDS-PAGE gel of CTB–LPETGA, oxidized CTB–LPETGA, biotinylated CTB–LPETGA, and C-terminally conjugated and biotinylated CTB constructs. Levels of conjugation listed in Table 2.

The N-terminal threonine residue was oxidized using sodium periodate (5 eq) in sodium phosphate buffer. This reaction typically reaches completion within 5 min in sodium phosphate (Figures 7A,B), whereas the reaction does not reach completion in phosphate-buffered saline containing potassium ions, which are known to hinder periodate reactivity (Brabham et al., 2020). Previously oxime ligation of alkoxyamine–PEG_4_–biotin was used for the N-terminal biotinylation of wt CTB; this was sufficient to allow effective binding to streptavidin for phage display screening (Balmforth et al., 2021). Using the same alkoxyamine–PEG_4_–biotin (10 eq) in the presence of aniline (1% *v*/*v*) at 37°C, oxime biotinylation of the oxidized CTB reached completion within 16 h (Figure 7C). When the oxidized CTB was used below 200 µM, a mixture of labeled product (12,924.5 Da) and N-terminal cyclisation (12,508.3 Da) was observed (Rose et al., 1999). For optimal oxime ligation reactions, an oxidized CTB concentration above 400 µM was required to minimize N-terminal cyclisation and obtain quantitative N-terminal biotinylation of both CTB–LPETGA and the truncated CTB.

Having achieved quantitative N-terminal biotinylation, preparative scale sortase ligation of the biotin–CTB–LPETGA with the (AEEAc)_2_ peptides and glycopeptides (**10**, **13**–**18**) was performed using CBD-Sortase 7M in the presence of 10% DMSO. After 2 h, each reaction was placed on ice and CBD-Sortase 7M removed using chitin resin. The level of conjugation was evaluated by SDS-PAGE and densitometry following purification by size exclusion chromatography to remove the excess peptide/glycopeptide (Figure 7D; Table 2). No significant difference in sortase ligation efficiency was observed between CTB–LPETGA with and without the N-terminal biotin tag.

**TABLE 2:** Percentage of conjugated and unlabeled sortase sites on Biotin–CTB–LPETGA after 2 h.

Substrate	Conjugated (%)^a^
13	85 (±3)
14	84 (±1)
15	85 (±1)
16	85 (±5)
17	87 (±4)
18	84 (±2)
10	82 (±6)

a Densitometry performed by comparing intensity of unlabeled (Biotin–CTB–LPETGA) and conjugated Biotin-CTB bands. Biotin-CTB without the LPETGA tag (CTB) accounts for ∼10% of each sample. Average of three technical repeats of the densitometry measurements. Errors indicate 2 × standard deviation.

## Conclusion

In conclusion, we have described the optimization of C-terminal sortase ligation for a series of MUC1(Tn) glycopeptide epitopes and the orthogonal labeling of both the N- and C-terminus of the cholera toxin B subunit using two site-specific methods. The N-terminus of CTB–LPETGA was quantitatively labeled with an alkoxyamine-functionalized biotin *via* oxime ligation. Sortase-mediated ligation could then be used to ligate a series of MUC1(Tn) epitopes to the C-terminus with a conjugation efficiency of 82%–87%. Although demonstrated here with biotinylation, it should be straight forward to extend N-terminal functionalization with other peptides, glycopeptides, or immunogenic epitopes given the ease of incorporation of alkoxyamine functionalization to small molecules or to peptides during solid-phase peptide synthesis. Furthermore, we have demonstrated the addition of an N-terminal chitin-binding domain to Sortase 7M has no negative effect enzyme activity and can be used to remove the enzyme rapidly and efficiently once the deemed endpoint of the reaction has been reached.

## Data Availability

The original contributions presented in the study are included in the article/Supplementary Material; the raw data associated with this paper including SDS-PAGE images, NMR and mass spectra are openly available from the University of Leeds data repository. https://doi.org/10.5518/1194.

## References

[B1] Agard N. J. Prescher J. A. Bertozzi C. R. (2004). A strain-promoted [3 + 2] Azide−Alkyne cycloaddition for covalent modification of biomolecules in living systems. J. Am. Chem. Soc. 126, 15046–15047. 10.1021/ja044996f 15547999

[B2] Apostolopoulos V. Chelvanayagam G. Xing P.-X. McKenzie I. F. C. (1998). Anti-MUC1 antibodies react directly with MUC1 peptides presented by class I H2 and HLA molecules. J. Immunol. 161, 767–775. 9670953

[B3] Awaya H. Takeshima Y. Yamasaki M. Inai K. (2004). Expression of MUC1, MUC2, MUC5AC, and MUC6 in atypical adenomatous hyperplasia, bronchioloalveolar carcinoma, adenocarcinoma with mixed subtypes, and mucinous bronchioloalveolar carcinoma of the lung. Am. J. Clin. Pathol. 121, 644–653. 10.1309/U4WG-E9EB-FJN6-CM8R 15151204

[B4] Baldus S. E. Engelmann K. Hanisch F.-G. (2004). MUC1 and the MUCs: a family of human mucins with impact in cancer biology. Crit. Rev. Clin. Lab. Sci. 41, 189–231. 10.1080/10408360490452040 15270554

[B5] Balmforth M. R. Haigh J. Kumar V. Dai W. Tiede C. Tomlinson D. C. (2021). Piggybacking on the cholera toxin: Identification of a CTB-binding protein as an approach for targeted delivery of proteins to motor neurons. Bioconjug. Chem. 32, 2205–2212. 10.1021/ACS.BIOCONJCHEM.1C00373 34565149

[B6] Brabham R. L. Keenan T. Husken A. Bilsborrow J. McBerney R. Kumar V. (2020). Rapid sodium periodate cleavage of an unnatural amino acid enables unmasking of a highly reactive α-oxo aldehyde for protein bioconjugation. Org. Biomol. Chem. 18, 4000–4003. 10.1039/D0OB00972E 32427272

[B7] Branson T. R. McAllister T. E. Garcia-Hartjes J. Fascione M. A. Ross J. F. Warriner S. L. (2014). A protein-based pentavalent inhibitor of the cholera toxin B-subunit. Angew. Chem. Int. Ed. 53, 8323–8327. 10.1002/anie.201404397 PMC4499251 24989497

[B8] Brayman M. Thathiah A. Carson D. D. (2004). MUC1: a multifunctional cell surface component of reproductive tissue epithelia. Reprod. Biol. Endocrinol. 2, 4. 10.1186/1477-7827-2-4 14711375 PMC320498

[B9] Bruins J. J. Blanco-Ania D. van der Doef V. van Delft F. L. Albada B. (2018). Orthogonal, dual protein labelling by tandem cycloaddition of strained alkenes and alkynes to *ortho* -quinones and azides. Chem. Commun. 54, 7338–7341. 10.1039/C8CC02638F 29911239

[B10] Burchell J. M. Mungul A. Taylor-Papadimitriou J. (2001). O-Linked glycosylation in the mammary gland: changes that occur during malignancy. J. Mammary Gland. Biol. Neoplasia 6, 355–364. 10.1023/a:1011331809881 11547903

[B11] Chen I. Dorr B. M. Liu D. R. (2011). A general strategy for the evolution of bond-forming enzymes using yeast display. Proc. Natl. Acad. Sci. U. S. A. 108, 11399–11404. 10.1073/pnas.1101046108 21697512 PMC3136257

[B12] Chen J. Zeng W. Offord R. Rose K. (2003). A novel method for the rational construction of well-defined immunogens: the use of oximation to conjugate cholera toxin B subunit to a Peptide−Polyoxime complex. Bioconjug. Chem. 14, 614–618. 10.1021/bc025651u 12757387

[B13] Chen L. Cohen J. Song X. Zhao A. Ye Z. Feulner C. J. (2016). Improved variants of SrtA for site-specific conjugation on antibodies and proteins with high efficiency. Sci. Rep. 6, 31899. 10.1038/srep31899 27534437 PMC4989145

[B14] Dadová J. Galan S. R. Davis B. G. (2018). Synthesis of modified proteins via functionalization of dehydroalanine. Curr. Opin. Chem. Biol. 46, 71–81. 10.1016/j.cbpa.2018.05.022 29913421

[B15] De Silva R. A. Wang Q. Chidley T. Appulage D. K. Andreana P. R. (2009). Immunological response from an entirely carbohydrate antigen: design of synthetic vaccines based on Tn-PS A1 conjugates. J. Am. Chem. Soc. 131, 9622–9623. 10.1021/ja902607a 19552408

[B16] Dertzbaugh M. T. Cox L. M. (1998). The affinity of cholera toxin for Ni^2+^ ion. Protein Eng. Des. Sel. 11, 577–581. 10.1093/protein/11.7.577 9740376

[B17] Dokurno P. Bates P. A. Band H. A. Stewart L. M. D. Lally J. M. Burchell J. M. (1998). Crystal structure at 1.95 å resolution of the breast tumour-specific antibody SM3 complexed with its peptide epitope reveals novel hypervariable loop recognition. J. Mol. Biol. 284, 713–728. 10.1006/jmbi.1998.2209 9826510

[B18] Doores K. J. Gamblin David. P. Davis B. G. (2006). Exploring and exploiting the therapeutic potential of glycoconjugates. Chem. Eur. J. 12, 656–665. 10.1002/chem.200500557 16187378

[B19] Fang T. Van Elssen C. H. M. J. Duarte J. N. Guzman J. S. Chahal J. S. Ling J. (2017). Targeted antigen delivery by an anti-class II MHC VHH elicits focused αMUC1(Tn) immunity. Chem. Sci. 8, 5591–5597. 10.1039/C7SC00446J 28970938 PMC5618788

[B20] Fottner M. Weyh M. Gaussmann S. Schwarz D. Sattler M. Lang K. (2021). A modular toolbox to generate complex polymeric ubiquitin architectures using orthogonal sortase enzymes. Nat. Commun. 12, 6515. 10.1038/s41467-021-26812-9 34764289 PMC8585875

[B21] Gendler S. J. (2001). MUC1, the renaissance molecule. J. Mammary Gland. Biol. Neoplasia 6, 339–353. 10.1023/A:1011379725811 11547902

[B22] Geoghegan K. F. Stroh J. G. (1992). Site-directed conjugation of nonpeptide groups to peptides and proteins via periodate oxidation of a 2-amino alcohol. application to modification at N-terminal serine. Bioconjug. Chem. 3, 138–146. 10.1021/bc00014a008 1325188

[B23] Godula K. Bertozzi C. R. (2012). Density variant glycan microarray for evaluating cross-linking of mucin-like glycoconjugates by lectins. J. Am. Chem. Soc. 134, 15732–15742. 10.1021/ja302193u 22967056 PMC3458438

[B24] Goins B. Friere E. (1988). Thermal stability and intersubunit interactions of cholera toxin in solution and in association with its cell-surface receptor ganglioside GM1. Biochemistry 27, 2046–2052. 10.1021/bi00406a035 3378043

[B25] Guo X. Wang Q. Swarts B. M. Guo Z. (2009). Sortase-catalyzed peptide−Glycosylphosphatidylinositol analogue ligation. J. Am. Chem. Soc. 131, 9878–9879. 10.1021/ja903231v 19583255

[B26] Haigh J. L. Williamson D. J. Poole E. Guo Y. Zhou D. Webb M. E. (2020). A versatile cholera toxin conjugate for neuronal targeting and tracing. Chem. Commun. 56, 6098–6101. 10.1039/D0CC01085E 32355935

[B27] Harmand T. J. Bousbaine D. Chan A. Zhang X. Liu D. R. Tam J. P. (2018). One-pot dual labeling of IgG 1 and preparation of C-to-C fusion proteins through a combination of sortase A and butelase 1. Bioconjug. Chem. 29, 3245–3249. 10.1021/acs.bioconjchem.8b00563 30231608 PMC6429940

[B28] Hattrup C. L. Gendler S. J. (2008). Structure and function of the cell surface (tethered) mucins. Annu. Rev. Physiol. 70, 431–457. 10.1146/annurev.physiol.70.113006.100659 17850209

[B29] Hirakawa H. Ishikawa S. Nagamune T. (2012). Design of Ca ^2+^ -independent *Staphylococcus aureus* sortase a mutants. Biotechnol. Bioeng. 109, 2955–2961. 10.1002/bit.24585 22729808

[B30] Hollingsworth M. A. Swanson B. J. (2004). Mucins in cancer: protection and control of the cell surface. Nat. Rev. Cancer 4, 45–60. 10.1038/nrc1251 14681689

[B31] Holmgren J. Adamsson J. Anjuère F. Clemens J. Czerkinsky C. Eriksson K. (2005). Mucosal adjuvants and anti-infection and anti-immunopathology vaccines based on cholera toxin, cholera toxin B subunit and CpG DNA. Immunol. Lett. 97, 181–188. 10.1016/j.imlet.2004.11.009 15752556

[B32] Holmgren J. Lycke N. Czerkinsky C. (1993). Cholera toxin and cholera B subunit as oral-mucosal adjuvant and antigen vector systems. Vaccine 11, 1179–1184. 10.1016/0264-410x(93)90039-z 8256498

[B33] Jackson M. Mantsch H. H. (1991). Beware of proteins in DMSO. Biochimica Biophysica Acta - Protein Struct. Mol. Enzym. 1078, 231–235. 10.1016/0167-4838(91)90563-F 2065090

[B34] Krishn S. R. Kaur S. Smith L. M. Johansson S. L. Jain M. Patel A. (2016). Mucins and associated glycan signatures in colon adenoma–carcinoma sequence: prospective pathological implication(s) for early diagnosis of colon cancer. Cancer Lett. 374, 304–314. 10.1016/j.canlet.2016.02.016 26898938 PMC4881851

[B35] Machin D. C. Williamson D. J. Fisher P. Miller V. J. Wildsmith G. C. Ross J. F. (2019). Sortase-modified cholera toxoids show specific golgi localization. 10.26434/CHEMRXIV.9125201.V1 PMC11054894 38668619

[B36] Martínez‐Sáez N. Castro-López J. Valero-González J. Madariaga D. Compañón I. Somovilla V. J. (2015). Deciphering the non-equivalence of serine and threonine O -glycosylation points: implications for molecular recognition of the Tn antigen by an anti‐MUC1 antibody. Angew. Chem. Int. Ed. 54, 9830–9834. 10.1002/anie.201502813 PMC4552995 26118689

[B37] Matos M. J. Brown L. Bernardim B. Guerreiro A. Jiménez-Osés G. Bernardes G. J. L. (2020). Sequential dual site-selective protein labelling enabled by lysine modification. Bioorg. Med. Chem. 28, 115783. 10.1016/j.bmc.2020.115783 33007561

[B38] Matsushita T. Sadamoto R. Ohyabu N. Nakata H. Fumoto M. Fujitani N. (2009). Functional neoglycopeptides: synthesis and characterization of a new class of MUC1 glycoprotein models having core 2-based O -glycan and complex-type N -glycan chains. Biochemistry 48, 11117–11133. 10.1021/bi901557a 19852465

[B39] Mazmanian S. K. Liu G. Ton-That H. Schneewind O. (1999). *Staphylococcus aureus* sortase, an enzyme that anchors surface proteins to the cell wall. Science 285, 760–763. 10.1126/science.285.5428.760 10427003

[B40] Mitchell S. A. Pratt M. R. Hruby V. J. Polt R. (2001). Solid-phase synthesis of O-linked glycopeptide analogues of enkephalin. J. Org. Chem. 66, 2327–2342. 10.1021/jo005712m 11281773

[B41] Morgan H. E. Turnbull W. B. Webb M. E. (2022). Challenges in the use of sortase and other peptide ligases for site-specific protein modification. Chem. Soc. Rev. 51, 4121–4145. 10.1039/D0CS01148G 35510539 PMC9126251

[B42] Munkley J. (2016). The role of sialyl-Tn in cancer. Int. J. Mol. Sci. 17, 275–283. 10.3390/ijms17030275 26927062 PMC4813139

[B43] Nath S. Mukherjee P. (2014). MUC1: a multifaceted oncoprotein with a key role in cancer progression. Trends Mol. Med. 20, 332–342. 10.1016/j.molmed.2014.02.007 24667139 PMC5500204

[B44] Pifferi C. Ruiz-de-Angulo A. Goyard D. Tiertant C. Sacristán N. Barriales D. (2020). Chemical synthesis and immunological evaluation of new generation multivalent anticancer vaccines based on a Tn antigen analogue. Chem. Sci. 11, 4488–4498. 10.1039/D0SC00544D 34122907 PMC8159477

[B45] Pizza M. Giuliani M. M. Fontana M. R. Monaci E. Douce G. Dougan G. (2001). Mucosal vaccines: non toxic derivatives of LT and CT as mucosal adjuvants. Vaccine 19, 2534–2541. 10.1016/s0264-410x(00)00553-3 11257389

[B46] Popp M. W. Antos J. M. Grotenbreg G. M. Spooner E. Ploegh H. L. (2007). Sortagging: a versatile method for protein labeling. Nat. Chem. Biol. 3, 707–708. 10.1038/nchembio.2007.31 17891153

[B47] Rashidian M. Kumarapperuma S. C. Gabrielse K. Fegan A. Wagner C. R. Distefano M. D. (2013). Simultaneous dual protein labeling using a triorthogonal reagent. J. Am. Chem. Soc. 135, 16388–16396. 10.1021/ja403813b 24134212 PMC3873327

[B48] Rose K. Chen J. Dragovic M. Zeng W. Jeannerat D. Kamalaprija P. (1999). New cyclization reaction at the amino terminus of peptides and proteins. Bioconjug. Chem. 10, 1038–1043. 10.1021/bc9900587 10563773

[B49] Saleeba C. Dempsey B. Le S. Goodchild A. McMullan S. (2019). A student’s guide to neural circuit tracing. Front. Neurosci. 13, 897. 10.3389/fnins.2019.00897 31507369 PMC6718611

[B50] Sánchez J. Holmgren J. (2008). Cholera toxin structure, gene regulation and pathophysiological and immunological aspects. Cell. Mol. Life Sci. 65, 1347–1360. 10.1007/s00018-008-7496-5 18278577 PMC11131847

[B51] Singh A. P. Chauhan S. C. Bafna S. Johansson S. L. Smith L. M. Moniaux N. (2006). Aberrant expression of transmembrane mucins, MUC1 and MUC4, in human prostate carcinomas. Prostate 66, 421–429. 10.1002/pros.20372 16302265

[B52] Smith J. J. Conrad D. W. Cuneo M. J. Hellinga H. W. (2009). Orthogonal site-specific protein modification by engineering reversible thiol protection mechanisms. Protein Sci. 14, 64–73. 10.1110/ps.04965405 PMC2253321 15576565

[B53] Spirig T. Weiner E. M. Clubb R. T. (2011). Sortase enzymes in Gram-positive bacteria. Mol. Microbiol. 82, 1044–1059. 10.1111/j.1365-2958.2011.07887.x 22026821 PMC3590066

[B54] Steinhagen M. Zunker K. Nordsieck K. Beck-Sickinger A. G. (2013). Large scale modification of biomolecules using immobilized sortase a from *Staphylococcus aureus* . Bioorg. Med. Chem. 21, 3504–3510. 10.1016/j.bmc.2013.03.039 23598248

[B55] Tessier R. Ceballos J. Guidotti N. Simonet-Davin R. Fierz B. Waser J. (2019). Doubly orthogonal” labeling of peptides and proteins. Chem 5, 2243–2263. 10.1016/j.chempr.2019.06.022

[B56] Ton-That H. Mazmanian S. K. Faull K. F. Schneewind O. (2000). Anchoring of surface proteins to the cell wall of *Staphylococcus aureus* . J. Biol. Chem. 275, 9876–9881. 10.1074/jbc.275.13.9876 10734144

[B57] Ulrich S. Boturyn D. Marra A. Renaudet O. Dumy P. (2014). Oxime ligation: a chemoselective click-type reaction for accessing multifunctional biomolecular constructs. Chem. Eur. J. 20, 34–41. 10.1002/chem.201302426 24302514

[B58] Vercelli A. Repici M. Garbossa D. Grimaldi A. (2000). Recent techniques for tracing pathways in the central nervous system of developing and adult mammals. Brain Res. Bull. 51, 11–28. 10.1016/S0361-9230(99)00229-4 10654576

[B59] Wang Q. Chan T. R. Hilgraf R. Fokin V. v. Sharpless K. B. Finn M. G. (2003). Bioconjugation by copper(I)-Catalyzed azide-alkyne [3 + 2] cycloaddition. J. Am. Chem. Soc. 125, 3192–3193. 10.1021/ja021381e 12630856

[B60] Witte M. D. Wu T. Guimaraes C. P. Theile C. S. Blom A. E. M. Ingram J. R. (2015). Site-specific protein modification using immobilized sortase in batch and continuous-flow systems. Nat. Protoc. 10, 508–516. 10.1038/nprot.2015.026 25719269 PMC4757899

[B61] Wu Z. Guo X. Guo Z. (2011). Sortase a-catalyzed peptide cyclization for the synthesis of macrocyclic peptides and glycopeptides. Chem. Commun. 47, 9218. 10.1039/c1cc13322e PMC3174090 21738926

[B62] Wuethrich I. Peeters J. G. C. Blom A. E. M. Theile C. S. Li Z. Spooner E. (2014). Site-specific chemoenzymatic labeling of aerolysin enables the identification of new aerolysin receptors. PLoS ONE 9, e109883. 10.1371/journal.pone.0109883 25275512 PMC4183550

[B63] Xu R. Hanson S. R. Zhang Z. Yang Y.-Y. Schultz P. G. Wong C.-H. (2004). Site-specific incorporation of the mucin-type N -acetylgalactosamine-α-O-threonine into protein in escherichia c oli. J. Am. Chem. Soc. 126, 15654–15655. 10.1021/ja044711z 15571382

[B64] Yi L. Sun H. Itzen A. Triola G. Waldmann H. Goody R. S. (2011). One-pot dual-labeling of a protein by two chemoselective reactions. Angew. Chem. Int. Ed. 50, 8287–8290. 10.1002/anie.201100840 21761514

[B65] Zhang D. Wang Z. Hu S. Balamkundu S. To J. Zhang X. (2021). pH-controlled protein orthogonal ligation using asparaginyl peptide ligases. J. Am. Chem. Soc. 143, 8704–8712. 10.1021/jacs.1c02638 34096285

