# Supplementary material for: Synthesis of cholera toxin B subunit glycoconjugates using site-specific orthogonal oxime and sortase ligation reactions
Source: Front Chem. 2022 Sep 14;10:958272. doi: 10.3389/fchem.2022.958272 (PMC9515619; doi:10.3389/fchem.2022.958272)

Supporting Information

Orthogonal site-specific N-terminal biotinylation and C-terminal glycopeptide labelling of Cholera toxin B-subunit

Jonathan P. Dolan[1†], Darren C. Machin[1], Simone Dedola[2], Robert A. Field[2,3], Michael E. Webb[1*], W. Bruce Turnbull[1*]

[1]School of Chemistry and Astbury Centre of Structural Biology, University of Leeds, Leeds, LS2 9JT, UK

[2]Iceni Glycoscience Ltd, Norwich Research Park, Norwich, NR4 7GJ, U.K.

[3]Department of Chemistry and Manchester Institute of Biotechnology, University of Manchester, Manchester, M1 7DN, U.K

[†]Present Address: School of Chemical and Physical Sciences & Centre for Glycoscience Research and Training, Keele University, Keele, Staffordshire, ST5 5BG, UK.

*** Correspondence:**

W. Bruce Turnbull	Michael E. Webb
w.b.turnbull@leeds.ac.uk	m.e.webb@leeds.ac.uk

Table of Contents

General Reagents & Methods 2

Synthesis of *N*-Fmoc-α-*O*-((AcO)$_3$GalNAc)-L-Serine and Threonine 3

Solid Phase Peptide Synthesis (SPPS) 9

General Biochemical Methods and Equipment 17

SDS-PAGE Gels of C-terminal Sortase Ligation Optimisation 22

SDS-PAGE Gels of CBD-Sortase7M Comparison and Optimisation 26

Protein HRMS Spectra of Oxime Ligation 28

References 30

NMR Spectra 31

HPLC Traces 48

General Reagents & Methods

Unless stated otherwise, all starting materials and reagents were purchased from commercial suppliers and used without further purification. All solvents used were dried prior to use, according to standard methods, unless otherwise stated. Reactions were performed under an N_2 atmosphere and within glassware which was oven dried. Completion of reactions was initially determined by TLC and visualized using shortwave ultraviolet light (254 nm) and/or charring with 5% H_2SO_4/MeOH. TLC plates used were Merck Silica-Gel 60 F^{254} Aluminium backed. Silica chromatography columns prepared using Fisher 60Å 43-60 micron silica gel. Lyophilisation carried out using Virtis Benchtop K freeze dryer. Centrifugation was performed using an Eppendorf Centrifuge 5810.

NMR spectroscopy was recorded using Bruker AV3HD-400 (400 MHz, BBO Probe), Bruker AV4 NEO (500 MHz, BBO, TXI & TBO Probe) and Bruker AV4 NEO (500 MHz, C/H cryoprobe) spectrometers. NMR data is reported in parts per million (ppm) referenced to residual solvent signal at room temperature (Fulmer et al., 2010). The following abbreviations are used in ^{1}H NMR analysis: Ar = aromatic, s = singlet, d = doublet, t = triplet, q = quartet, m= multiplet, dd = double doublet, dt = doublet of triplets, td = triplet of doublets and ddd = double double doublet.

HRMS was performed using Bruker Daltonics MicroTOF mass spectrometer employing electrospray (ES+) ionisation. LC-MS analysis performed on Brucker AmaZon X series LC-MS spectrometer. IR spectroscopy was recorded using a Bruker Platinum ATR spectrometer. Optical rotations were measured with a Schmidt and Haensch Polartronic H-532 at the sodium D-line with the $[\alpha]_D^{20}$ values given in the units 10^{-1} deg cm^2 g^{-1}.

Standard numbering conventions for carbohydrates in pyranoside and furanoside systems is followed (i.e numbering starts from the anomeric centre). Numbering schemes of more complex structures will be demonstrated on the structure provided.

Synthesis of *N*-Fmoc-α-*O*-((AcO)$_3$GalNAc)-L-Serine and Threonine

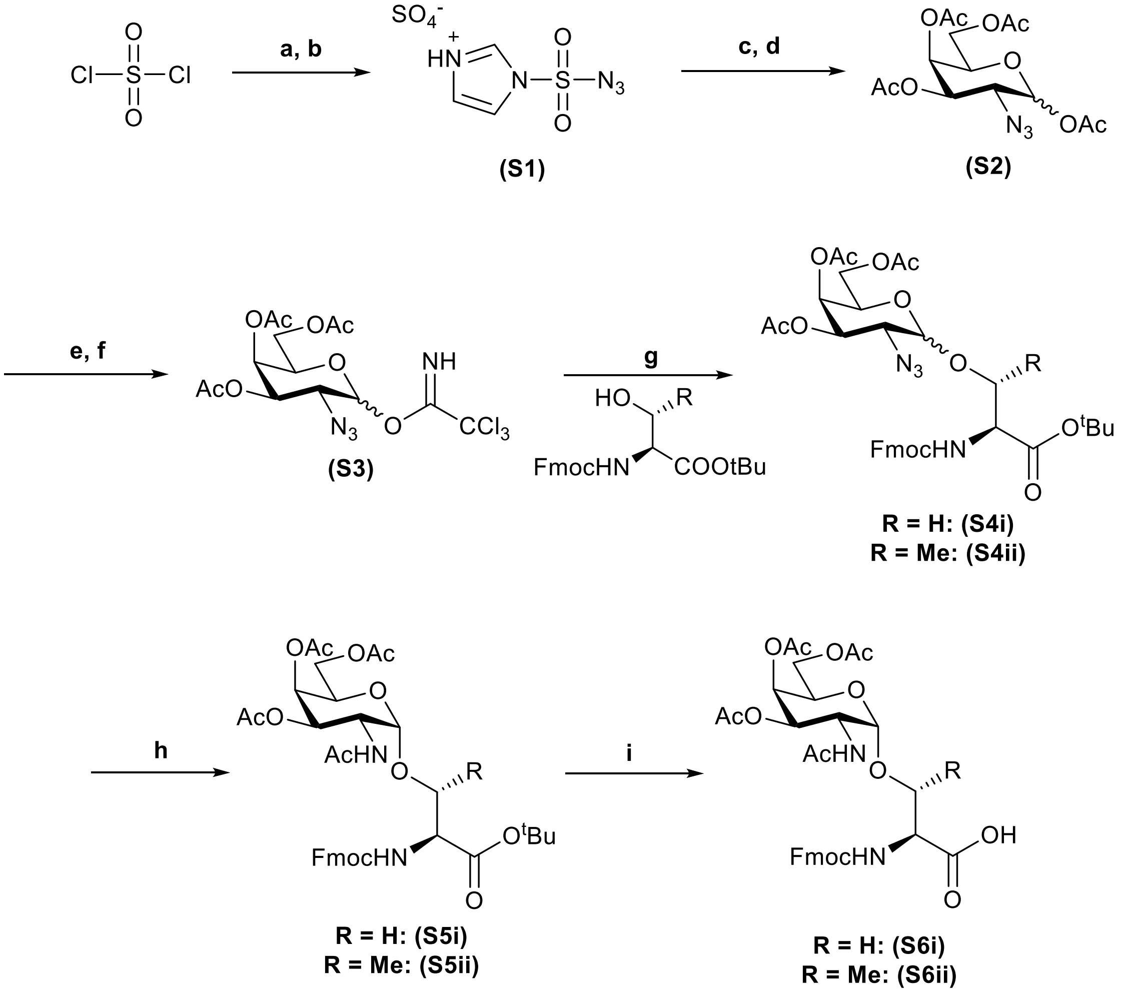

Scheme S1: *Synthesis of N-Fmoc-α-O-((AcO)$_3$GalNAc)-L-Serine and Threonine for use in solid phase peptide synthesis. a) NaN_3, EtOAc; b) Imidazole, H_2SO_4 (61%, 2 steps); c) D-Galactosamine, $CuSO_4$, K_2CO_3, MeOH, H_2O; d) Ac_2O, Pyridine, α/β=1/10 (62%, 2 steps); e) H_4N_2·AcOH, DMF; f) Cl_3CCN, DBU, DCM, α/β=10/1 (56%, 2 steps); g) TMSOTf, DCM, Et_2O, −30°C, α/β=1/1; h) Zn, $CuSO_4$, Ac_2O, AcOH, THF (**S5i**: 39%, **S5ii**: 40%, 2 steps); i) 95% TFA in H2O (**S6i**: 74%, **S6ii**: 92%).*

*Imidazole-sulfonyl-azide hydrogen sulfate salt (**S1**)*

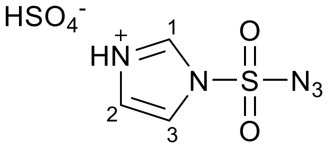

Imidazole-1-sulfonyl azide is highly explosive in its neutral form. Reaction should only be performed behind Perspex blast shield and no attempt to concentrate solutions of imidazole-1-sulfonyl azide should be made. Sodium azide and imidazole were dried overnight (12-14 hrs) in a vacuum desiccator over phosphorous pentoxide prior to use. Extra dry EtOAc over molecular sieves was purchased from Acros Organics; a fresh bottle was used for each reaction.

Sodium azide (5.00 g, 77 mmol, 1.0 eq) was placed in a dry 500 mL three neck RBF with a dry stirrer bead. Extra dry EtOAc (77 mL) was added to the flask and the resulting suspension cooled to 0 °C. Sulfuryl chloride (6.2 mL, 77 mmol, 1.0 eq) was added dropwise over 5 minutes and the mixture allowed to warm to room temperature and stirred for 24 hrs. The suspension was recooled to 0 °C and imidazole (10.00 g, 146 mmol, 1.9 eq) was added continuously over 5 minutes. The suspension was stirred at RT for 3 hrs. The mixture was basified by the addition of sat. aq. $NaHCO_3$ solution (150 mL). Once bubbling had ceased, the mixture was separated, the organic portion washed with water (150 mL), dried over $MgSO_4$ and filtered. Filtrate was recooled to 0 °C and placed under N_2 atmosphere. Conc. H_2SO_4 (4.1 mL, 77 mmol, 1.0 eq) was added dropwise over the course of 5 minutes and gradually warmed to room temperature with vigorous stirring. Over the course of 30 minutes, a colourless precipitate formed and was collected by vacuum filtration. Precipitate was washed with a small amount of ice-cold EtOAc and the crystals were dried under high vacuum to yield imidazole-1-sulfonyl azide hydrogen sulfate salt, **S1** (12.71 g, 46.8 mmol, 61%).

NMR data is in agreement with reported data (Potter et al., 2016).

^{1}H NMR (400 MHz, DMSO): δ 14.29 (1 H, br S, N**H**$^+$), 12.09 (1 H, br S, **H**SO_4^-), 9.07 (1 H, s, ***H$_1$***), 7.67 (2 H, s, ***H$_2$***, ***H$_3$***); **^{13}C NMR** (100 MHz, DMSO): δ 134.5 (C_1), 119.4 (C_2, C_3); **IR** (ν_{max}/cm^{-1}); 3083 (C-H stretch), 2860 (C-H stretch), 2177 (N=N=N stretch), 1586 (N-H bend), 1460 (S=O stretch); **HRMS** [ES+] found $[M-HSO_4^-]^+$ 174.0077, $C_3H_6N_5O_6S$ requires 174.0080.

1,3,4,6-Tetra-O-acetyl-2-azido-2-deoxy-D-galactopyranose (S2)

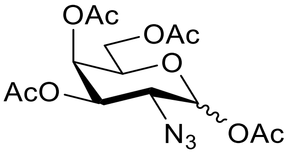

D-Galactosamine hydrochloride (1.00 g, 5.58 mmol, 1.0 eq). K_2CO_3 (2.16 g, 15.62 mmol, 2.8 eq) and $CuSO_4 \cdot 5H_2O$ (14 mg, 56 µmol, 1 mol%) were dissolved MeOH/H_2O (5:3, 29 mL). To this mixture imidazole-1-sulfonyl azide hydrogen sulfate, **S1**, (1.817 g, 6.70 mmol, 1.2 eq) was slowly added and left to stir at room temperature. After 3.5 hrs, the mixture was filtered through a pad of celite and co-evaporated with toluene. The water bath was not allowed to exceed 20 °C. Acetic anhydride (2.7 mL, 28.56 mmol, 5.1 eq) was added dropwise to the crude azido-galactose dissolved in pyridine (30 mL) and stirred overnight. Pyridine was removed *in vacuo*, co-evaporating with toluene twice. The residue dissolved in EtOAc (50 mL) and washed with sat. aq. $NaHCO_3$ solution (3 × 25 mL) and brine (1 × 25 mL). The organic extract was dried over $MgSO_4$, filtered, and concentrated to dryness. Crude product was purified using flash column chromatography (2:1 Hexane–EtOAc), to yield 1,3,4,6-tetra-*O*-acetyl-2-azido-2-deoxy-D-galactose, **S2** (1.30 g, 3.48 mmol, 62%, 1:10 α/β) as a colourless oil.

NMR data is in agreement with reported data (Alper et al., 1996; De Silva et al., 2009).

R$_f$ 0.41 (2:1 Hexane–EtOAc); **^{1}H NMR** (300 MHz, $CDCl_3$): δ 6.29 (1 H, d, *J* = 3.5 Hz, ***H$_{1\alpha}$***), 5.54 (1 H, d, *J* = 8.5 Hz, ***H$_{1\beta}$***), 5.44 (1 H, d, *J* = 2.4 Hz, ***H$_{4\alpha}$***), 5.35 (1 H, d, *J* = 3.1 Hz, ***H$_{4\beta}$***), 5.29 (1 H, dd, *J* = 11.0, 2.9 Hz, ***H$_{3\alpha}$***), 4.89 (1 H, dd, *J* = 10.8, 3.3 Hz, ***H$_{3\beta}$***), 4.13–3.95 (3 H, m, ***H$_5$***, ***H$_{6a}$***, ***H$_{6b}$***), 3.91 (1 H, dd, *J* = 11.0, 3.6 Hz, ***H$_{2\alpha}$***), 3.81 (1 H, dd, *J* = 10.3, 9.0 Hz, ***H$_{2\beta}$***), 2.17

(3 H, s, Me), 2.13 (3 H, s, Me), 2.03 (3 H, s, Me), 2.00 (3 H, s, Me); **^{13}C NMR** (75 MHz, $CDCl_3$): δ 170.2 (C=O), 169.8 (C=O), 169.5 (C=O), 168.4 (C=O), 92.7 ($C_{1\beta}$), 90.3 ($C_{1\alpha}$), 71.6 (C_6), 71.2 ($C_{3\beta}$), 68.6 ($C_{3\alpha}$), 66.8 ($C_{4\alpha}$), 66.1 ($C_{4\beta}$), 61.0 ($C_{5\alpha}$), 60.9 ($C_{5\beta}$), 59.6 ($C_{2\beta}$), 56.7 ($C_{2\alpha}$), 20.9 (Me), 20.7 (Me), 20.5 (Me), 20.5 (Me); **IR** (ν_{max}/cm^{-1}): 2939 (C-H stretch), 2112 (N=N=N stretch) and 1743 (C=O stretch); **HRMS** [ES+] found $[M+NH_4]^+$ 391.1458, $C_{14}H_{23}N_4O_9$ requires 391.1460.

3,4,6-Tri-O-acetyl-2-azido-2-deoxy-D-galactopyranosyl trichloroacetimidate (S3)

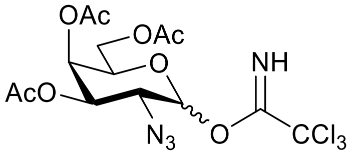

1,3,4,6-Tetra-*O*-acetyl-2-azido-2-deoxy-galactopyranose, **S2** (1.299 g, 3.48 mmol, 1.0 eq) and hydrazine acetate (385 mg, 4.18 mmol, 1.2 eq) were stirred in dry DMF (6 mL) and heated to 60 °C. After 1 hour, TLC showed reaction had reached completion and reaction was cooled to room temperature. Reaction was diluted with EtOAc (50 mL) and washed with water (2 × 50 mL) and brine (1 × 50 mL). Organic portion was dried over $MgSO_4$ and concentrated to dryness to yield the crude hemiacetal. Crude hemiacetal was dissolved in dry DCM (12 mL) and cooled to 0 °C. Trichloroacetonitrile (7.00 mL, 69.81 mmol, 20.0 eq) and DBU (260 µL, 1.74 mmol, 0.5 eq) were added and mixture stirred at 0 °C. After 2 hours, TLC showed no further reaction and reaction was diluted with DCM (40 mL) and washed with sat. aq. NH_4Cl solution (3 × 50 mL) and brine (1 × 50 mL), dried over $MgSO_4$, filtered and concentrated *in vacuo*. Crude trichloroacetimidate was purified by flash column chromatography (2:3 EtOAc–Hexane) to yield pure 3,4,6-tri-*O*-acetyl-2-azido-2-deoxy-D-galactopyranosyl trichloroacetimidate, **S3** (927 mg, 1.95 mmol, 56%, 10:1 α/β) as a yellow foam.

NMR data is in agreement with reported data for the α-anomer (Koeller et al., 2000).

R_f 0.38 (2:3 EtOAc–Hexane); **^{1}H NMR** (400 MHz, $CDCl_3$): δ 8.79 (1 H, br. s, N**H**), 6.78 (1 H, d, *J* = 3.6 Hz, ***H_1***), 5.50 (1 H, d, *J* = 2.3 Hz, ***H_4***), 5.34 (1 H, dd, *J* = 3.2, 11.1 Hz, ***H_3***), 4.38 (1 H, t, *J* = 6.7, 6.7 Hz, ***H_5***), 4.15–3.98 (3 H, m, ***H_2, H_{6a}, H_{6b}***), 2.13 (3 H, s, Me), 2.04 (3 H, s, Me), 1.97 (3 H, s, Me); **^{13}C NMR** (100 MHz, $CDCl_3$): δ 170.3 (C=O), 170.0 (C=O), 170.0 (C=O), 160.7 (C=N), 94.5 (C_1), 90.7 (CCl_3), 69.2 (C_5), 68.7 (C_3), 67.0 (C_4), 61.2 (C_6), 57.1 (C_2), 20.7 (Me), 20.6 (Me), 20.6 (Me); **IR** (ν_{max}/cm^{-1}): 3337 (C-H stretch), 3136 (C-H stretch), 2968 (N-H stretch), 2112 (N=N=N stretch), 1744 (C=O stretch) and 1676 (C=N stretch); **HRMS** [ES+] found $[M+Na]^+$ 497.0012, $C_{14}H_{17}Cl_3N_4O_8Na$ requires 497.0004.

*N-Fmoc-O-(3,4,6-tri-O-acetyl-2-azido-2-deoxy-α/β-D-galactopyranosyl)-L-serine (**S4i**) and threonine tert-butyl ester (**S4ii**)*

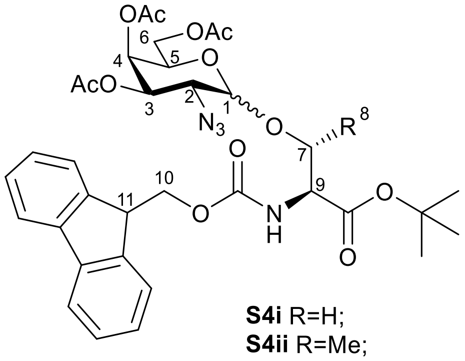

S4i R=H;
S4ii R=Me;

Trichloroacetimidate, **S3**, (927 mg, 1.95 mmol, 1.1 eq) and Fmoc-Ser-O^{t}Bu (679 mg, 1.77 mmol, 1.0 eq) or Fmoc-Thr-O^{t}Bu threonine derivative (704 mg, 1.77 mmol, 1.0 eq) were combined and dried overnight under vacuum, then dissolved in a mixture of dry DCM:Et_2O (1:1, 10 mL). After cooling to –30 °C, TMSOTf (42 µL, 0.23 mmol, 0.13 eq) was added dropwise. The mixture was stirred at –30 °C for 3 hours. The reaction mixture was quenched with DIPEA (53 µL, 0.30 mmol, 0.17 eq). The reaction mixture was diluted with DCM (40 mL) and washed with water (2 × 50 mL) and brine (1 × 50 mL), the organic portion was dried over $MgSO_4$, filtered and concentrated *in vacuo* to yield *N*-Fmoc-*O*-(3,4,6-tri-*O*-acetyl-2-azido-2-deoxy-α-D-galactopyranosyl)-L-serine tert-butyl ester, **S4i** (1.564 g, 1:1 α/β) or threonine derivative, **S4ii** (1.537 g, 1:1 α/β), both as yellow oils. Both **S4i** and **S4ii** were carried through to the next step without further purification.

NMR data is in agreement with reported data (Koeller et al., 2000).

S4i: ***R_f***: 0.26 (3:7 EtOAc–Hexane); **^{1}H NMR** (400 MHz, $CDCl_3$): δ 7.70 (4 H, d, *J* = 7.6 Hz, Ar**H**), 7.52 (4 H, d, *J* = 7.2 Hz, Ar**H**), 7.34 (4 H, t, *J* = 7.5 Hz, Ar**H**), 7.25 (4 H, t, *J* = 7.5 Hz, Ar**H**), 5.60 (2 H, d, *J* = 6.3 Hz, N**H**(Thr)), 5.47 (1 H, d, *J* = 8.5 Hz, ***H_1***), 5.31 (1 H, d, *J* = 3.4 Hz, ***H_4***), 4.82 (1 H, dd, *J* = 10.7, 4.1 Hz, ***H_3***), 4.35 (4 H, d, *J* =7.3 Hz, ***H_6***), 4.29–4.22 (2 H, m, ***H_9***), 4.16 (2 H, t, *J* = 7.3 Hz, ***H_5***), 4.11–4.00 (4 H, m, ***H_{10}***), 3.93 (1 H, t, *J* = 6.6 Hz, ***H_{11}***), 3.90–3.82 (4 H, m, ***H_7***), 3.77 (1 H, dd, *J* = 11.6, 8.1 Hz, ***H_2***), 2.29 (6 H, s, Me), 2.12 (6 H, s, Me), 1.99 (6 H, s, Me), 1.42 (18 H, s, Me); **HRMS** [ES+] found $[M+H]^+$ 697.2712, $C_{35}H_{43}N_4O_{12}$ requires 697.2715.

S4ii: ***R_f*** 0.51 (2:3 EtOAc–Hexane); **^{1}H NMR** (400 MHz, $CDCl_3$): δ 7.69 (4 H, dd, *J* = 7.7, 3.9 Hz, Ar**H**), 7.55 (4 H, dt, *J* = 8.0, 2.7 Hz, Ar**H**), 7.31 (4 H, dt, *J* = 7.2, 2.4 Hz, Ar**H**), 7.10 (4 H, dd, *J* = 7.7, 3.2 Hz, Ar**H**), 5.61 (1 H, d, *J* = 9.8 Hz, N**H**$(Thr)_α$), 5.50 (1 H, d, *J* = 9.8 Hz, N**H**$(Thr)_β$), 5.40 (1 H, d, *J* = 3.0 Hz, ***$H_{4α}$***), 5.27 (1 H, dd, *J* = 10.4, 3.3 Hz, ***$H_{3α}$***), 5.24 (1 H, d, *J* = 3.0, ***$H_{4β}$***), 5.04 (1 H, d, *J* = 3.8 Hz, ***$H_{1α}$***), 4.69 (1 H, dd, *J* = 10.7, 3.3 Hz, ***$H_{3β}$***), 4.51 (1 H, qd, *J* = 12.4, 5.9, 2.1 Hz, ***$H_{7α}$***), 4.41 (1 H, d, *J* = 8.0, ***$H_{1β}$***), 4.38 (1 H, dd, *J* = 6.8, 1.4 Hz, ***$H_{7β}$***), 4.34 (1 H, dd, *J* = 7.7, 2.7 Hz, ***$H_{9β}$***), 4.34–4.27 (3 H, m, ***$H_{5α}$, $H_{5β}$***), 4.27–4.13 (5 H, m, ***$H_{9α}$, $H_{6α}$, $H_{6β}$***), 4.04 (4 H, dd, *J* = 6.6, 2.7 Hz, ***$H_{10α}$, $H_{10β}$***), 3.78 (1 H, t, *J* = 6.8 Hz, ***H_{11}***), 3.57 (2 H, dd, *J* = 11.1, 3.4 Hz, ***$H_{2α}$, $H_{2β}$***), 2.28 (6 H, s, Me), 2.08 (6 H, s, Me), 2.00 (6 H, s, Me), 1.43 (18 H, s, t-Bu(Me)), 1.27 (6 H, dd, *J* = 12.8, 6.2 Hz, Me-8); **IR** ($ν_{max}/cm^{-1}$): 3354 (C-H stretch), 2979 (N-H stretch), 2111 (N=N=N stretch), 1721 (C=O stretch); **HRMS** [ES+] found $[M+Na]^+$ 733.2704, $C_{35}H_{42}N_4O_{12}Na$ requires 733.2691.

*N-Fmoc-O-(2-acetimido-2-deoxy-3,4,6-Tri-O-acetyl-α-D-galactopyranosyl)-L-serine (**S5i**) and threonine tert-butyl ester (**S5ii**)*

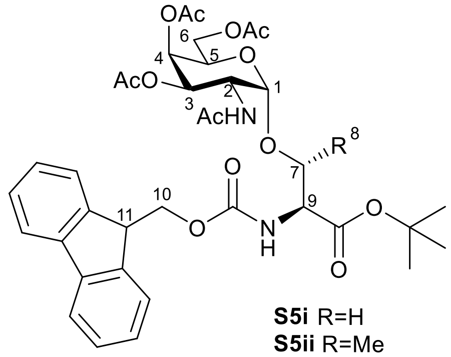

S5i R=H
S5ii R=Me

Zinc dust (15.3 g, 234 mmol, 17.5 eq) was activated in 2% aq. $CuSO_4$ (120 mL). After 10 minutes, the zinc/copper mixture was added to crude **S4i** (9.34 g, 13.4 mmol, 1.0 eq) or crude **S4ii** (9.51 g, 13.4 mmol, 1.0 eq) dissolved in a mixture of THF/AcOH/Ac_2O (3:2:1, 180 ml). The mixture was stirred at room temperature for 4 hours. Once evolution of gas had ceased and TLC showed the reaction had reached completion; the mixture was filtered through celite and the pad was washed with THF (60 mL). The filtrate was concentrated in vacuo and residue dissolved in DCM (300 mL) and washed with 0.1 M HCl (2 × 400 mL). The organic portion was dried over $MgSO_4$, filtered and concentrated *in vacuo*. Crude acetamide was purified by flash column chromatography (1% MeOH in DCM) to yield pure **S5i** (2.626 g, 3.68 mmol, 39%) or **S5ii** (3.113 g, 4.28 mmol, 40%) as glassy white foams.

NMR data is in agreement with reported data (Koeller et al., 2000).

S5i: **$[\alpha]_D^{25}$** +59 (c 1, chloroform); **R_f** 0.65 (15:1 DCM–MeOH); **^{1}H NMR** (400 MHz, DMSO-*d_6*): δ 7.76 (2 H, d, *J* = 7.5 Hz, Ar**H**), 7.61 (2 H, d, *J* = 7.1 Hz, Ar**H**), 7.40 (2 H, t, *J* = 7.4 Hz, Ar**H**), 7.32 (2 H, t, *J* = 7.4 Hz, Ar**H**), 5.80 (2 H, d, *J* = 7.9 Hz, N**H**Ac, N**H**(Ser)), 5.37 (1 H, d, *J* = 2.8 Hz, $\boldsymbol{H_4}$), 5.12 (1 H, dd, *J* = 11.2, 2.1 Hz, $\boldsymbol{H_3}$), 4.83 (1 H, d, *J* = 3.3 Hz, $\boldsymbol{H_1}$), 4.59 (1 H, td, *J* = 11.1, 3.3 Hz, $\boldsymbol{H_2}$), 4.46–4.39 (3 H, m, $\boldsymbol{H_9}$, $\boldsymbol{H_{10}}$), 4.24 (1 H, t, *J* = 6.9 Hz, $\boldsymbol{H_{11}}$), 4.15–3.99 (3 H, m, $\boldsymbol{H_5}$, $\boldsymbol{H_6}$), 3.96 (1 H, d, *J* = 9.5 Hz, $\boldsymbol{H_{7a}}$), 3.84 (1 H, d, *J* = 8.5 Hz, $\boldsymbol{H_{7b}}$), 2.15 (3 H, s, Me), 1.99 (3 H, s, Me), 1.99 (3 H, s, Me), 1.93 (3 H, s, Me), 1.48 (9 H, s, t-Bu(Me)); **^{13}C NMR** (100 MHz, DMSO-*d_6*): δ 170.9 (C=O),170.4 (C=O), 170.3 (C=O), 170.1 (C=O), 169.0 (C=O), 155.9 (C=O), 143.8 (Ar), 141.3 (Ar), 127.8 (Ar), 127.1 (Ar), 125.1 (Ar), 120.1 (Ar), 98.8 (C_1), 83.1 (tBu(quat. C)), 69.3 (C_7), 68.4 (C_3), 67.3 (C_4), 67.3 (C_5), 62.0 (C_6), 54.8 (C_9), 53.5 (C_{10}), 47.6 (C_2), 47.1 (C_{11}), 28.1 (tBu(Me)), 23.2 (Me), 20.8 (Me), 20.8 (Me), 20.6 (Me); **HRMS** [ES+] found $[M+H]^+$ 713.2925, $C_{36}H_{45}N_2O_{13}$ requires 713.2916.

S5ii: **$[\alpha]_D^{25}$** +62 (*c* 1, chloroform); ***R_f*** 0.83 (15:1 DCM–MeOH); **^{1}H NMR** (400 MHz, $CDCl_3$): δ 7.70 (2 H, d, *J* = 7.1 Hz, Ar**H**), 7.56 (2 H, d, *J* = 7.2 Hz, Ar**H**), 7.33 (2 H, t, *J* = 6.5, 6.5 Hz, Ar**H**), 7.25 (2 H, t, *J* = 7.2, 7.2 Hz, Ar**H**), 6.01 (1 H, d, *J* = 9.9 Hz, N**H**(Thr)), 5.62 (1 H, d, *J* = 9.4 Hz, N**H**Ac), 5.31 (1 H, d, *J* = 2.5 Hz, $\boldsymbol{H_4}$), 5.02 (1 H, dd, *J* = 11.3, 2.5 Hz, $\boldsymbol{H_3}$), 4.82 (1 H, d, *J* = 3.1 Hz, $\boldsymbol{H_1}$), 4.55 (1 H, td, *J* = 10.5, 10.5, 3.1 Hz, $\boldsymbol{H_2}$), 4.45–4.31 (2 H, m, $\boldsymbol{H_{6a}}$, $\boldsymbol{H_{6b}}$), 4.22–4.12 (4 H, m, $\boldsymbol{H_{11}}$, $\boldsymbol{H_9}$, $\boldsymbol{H_7}$, $\boldsymbol{H_5}$), 4.04-3.97 (2 H, m, $\boldsymbol{H_{10}}$), 2.08 (3 H, s, Me), 1.96 (3 H, s, Me), 1.92 (6 H, s, Me), 1.32 (9 H, s, OtBu), 1.24 (3 H, d, *J* = 6.1 Hz, $\boldsymbol{H_8}$); **^{13}C NMR** (100 MHz, $CDCl_3$): 170.8 (C=O), 170.3 (C=O), 170.2 (C=O), 169.9 (C=O), 156.4 (C=O), 143.6 (Ar), 141.2 (Ar), 127.7 (Ar), 127.0 (Ar), 125.0 (Ar), 119.9 (Ar), 99.8 (C_1), 83.1 (tBu(quat. C)), 76.8 (C_{11}), 68.6 (C_3), 67.3 (C_4), 67.2 (C_5), 67.1 (C_6), 62.1 (C_{10}), 58.9 (C_9), 47.2 (C_2), 47.1 (C_7), 28.0 (tBu(Me)), 23.1 (Me), 20.6 (Me), 20.5 (Me), 18.5 (C_8); **IR** (ν_{max}/cm^{-1}): 3256 (s, C-H stretch),

2980 (s, C-H stretch), 1748 (s, C=O (ester) stretch), 1651 (s, C=O (amide) stretch); **HRMS** [ES+] found [M+H]$^+$ 727.3072, $C_{37}H_{47}N_2O_{13}$ requires 727.3073).

*N-Fmoc-O-(2-acetimido-2-deoxy-3,4,6-Tri-O-acetyl-α-D-galactopyranosyl)-L-serine (**S6i**) and threonine (**S6ii**)*

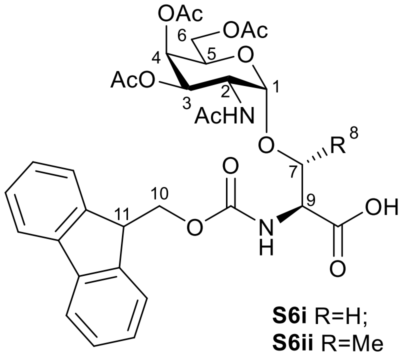

S5i (1.00 g, 1.38 mmol, 1.0 eq) or **S5ii** (1.00 g, 1.40 mmol, 1.0 eq) dissolved in 95% TFA (3.0 mL) and stirred at room temperature for 1 hour. Solvent was removed *in vacuo* and co-evaporated with toluene. The crude product purified by flash column chromatography (5:1 Toluene–EtOH) to yield **S6i** (671 mg, 1.02 mmol, 74%) or **S6ii** (854 mg, 1.27 mmol, 92%) as a glassy off-white foams.

NMR data is in agreement with reported data (Koeller et al., 2000).

S6i: [α]$_D$^{25} +88 (*c* 1, chloroform); **R*f*** 0.47 (5:1 Toluene–EtOH); **^{1}H NMR** (500 MHz, DMSO-*d*$_6$): δ 7.90 (2 H, d, J = 7.5 Hz, Ar**H**), 7.81 (1 H, d, J = 8.4 Hz, N**H**Ac), 7.73 (1 H, d, J = 8.4 Hz, N**H**(Ser)), 7.72 (2 H, d, J = 7.1, Ar**H**), 7.42 (2 H, t, J = 7.7 Hz, Ar**H**), 7.33 (2 H, t, J = 7.8 Hz, Ar**H**), 5.31 (1 H, d, J = 2.6 Hz, ***H$_4$***), 5.07 (1 H, dd, J = 11.6, 3.2 Hz, ***H$_3$***), 4.86 (1 H, d, J = 3.6 Hz, ***H$_1$***), 4.38 (2 H, dd, J = 7.4, 3.6 Hz, ***H$_6$***), 4.31–4.23 (3 H, m, ***H$_5$***, ***H$_9$***, ***H$_{11}$***), 4.20 (1 H, ddd, J = 11.7, 8.4, 3.6 Hz, ***H$_2$***), 4.05 (1 H, dd, J = 11.3, 5.8 Hz, ***H$_{10a}$***), 3.98 (1 H, dd, J = 10.6, 7.0 Hz, ***H$_{10b}$***), 3.83 (1 H, dd, J = 10.9, 4.1 Hz, ***H$_{7a}$***), 3.77 (1 H, dd, J = 10.9, 4.8 Hz, ***H$_{7b}$***), 2.10 (3H, s, Me), 1.95 (3H, s, Me), 1.90 (3H, s, Me), 1.81 (3H, s, Me); **^{13}C NMR** (125 MHz, DMSO-*d*$_6$): δ 171.5 (C=O), 170.1 (C=O), 169.9 (C=O), 169.8 (C=O), 169.7 (C=O), 156.2 (C=O), 143.9 (Ar), 143.8 (Ar), 140.8 (Ar), 137.4 (Ar), 128.9 (Ar), 128.2 (Ar), 125.3 (Ar), 125.2 , 120.2 (Ar), 97.9 (C_1), 67.6 (C_7), 67.5 (C_3), 67.1 (C_4), 66.3 (C_5), 65.7 (C_6), 61.6 (C_{10}), 54.3 (C_9), 47.1 (C_2), 46.7 (C_{11}), 22.5 (Me), 21.1 (Me), 20.6 (Me), 20.5 (Me); **HRMS** [ES+] found [M+H]$^+$ 657.2291, $C_{32}H_{37}N_2O_{13}$ requires 657.2290.

S6ii: [α]$_D$^{25} +72 (*c* 1, chloroform); **R*f*** 0.31 (5:1 Toluene–EtOH); **^{1}H NMR** (400 MHz, DMSO-*d*$_6$): δ 7.30 (2 H, d, *J* = 7.5 Hz, Ar**H**), 7.74 (2 H, dd, *J* = 7.2, 3.6 Hz, Ar**H**), 7.67 (1 H, d, *J* = 9.4 Hz, N**H**Ac), 7.59 (1 H, d, *J* = 9.8 Hz, N**H**(Thr)), 7.42 (2 H, t, *J* = 7.4 Hz, Ar**H**), 7.33 (2 H, t, *J* = 7.2 Hz, Ar**H**), 5.32 (1 H, d, *J* = 2.8 Hz, ***H$_4$***), 5.05 (1 H, dd, *J* = 11.6, 3.2 Hz, ***H$_3$***), 4.81 (1 H, d, *J* = 3.8 Hz, ***H$_1$***), 4.45 (2 H, ddd, *J* = 25.8, 10.8, 7.0 Hz, ***H$_6$***), 4.34–4.27 (2 H, m, ***H$_7$***, ***H$_5$***), 4.26–4.19 (2 H, m, ***H$_{11}$***, ***H$_2$***), 4.15 (1 H, dd, *J* = 9.9, 1.2 Hz, ***H$_9$***), 4.03 (2 H, d, *J* = 6.1 Hz, ***H$_{10}$***), 2.11 (3H, s, Me), 1.99 (3H, s, Me), 1.91 (3H, s, Me), 1.84 (3H, s, Me), 1.18 (3 H, d, *J* = 6.4 Hz, ***H$_8$***); **^{13}C NMR** (100 MHz, DMSO-*d*$_6$): δ 170.7 (C=O), 170.1 (C=O), 170.0 (C=O), 169.9 (C=O), 169.5 (C=O), 156.9 (C=O), 143.8 (Ar), 140.8 (Ar), 127.7 (Ar), 127.1 (Ar), 125.2 (Ar), 120.2 (Ar), 98.8 (C_1), 74.9 (C_7), 67.6 (C_3), 67.2 (C_4), 66.4 (C_{11}), 65.6 (C_6), 62.0 (C_{10}), 58.4

(C_9), 46.8 (C_5), 46.6 (C_2), 22.7 (Me), 20.5 (Me), 18.6 (C_8); **IR** (ν_{max}/cm^{-1}): 3329 (s, C-H stretch), 2941 (s, C-H stretch), 1744 (s, C=O (ester) stretch), 1657 (s, C=O (amide) stretch); **HRMS** [ES+] found $[M+H]^+$ 671.2450, $C_{33}H_{39}N_2O_{13}$ requires 671.2447.

Solid Phase Peptide Synthesis (SPPS)

General Reagents & Equipment for SPPS

All amino acids, resins and coupling reagents were purchased from Novabiochem or Fluorochem and used without further purification. Fritted polypropylene tubes (2.5 mL) were purchased from Supelco (Merck) and were used for all solid phase reactions. Agitation of solid phase reaction mixture was achieved by rotation on a Stuart blood rotator at room temperature. Peptides and glycopeptides were synthesised as C-terminal amides using Rink Amide MBHA LL resin (50 µM, loading 0.35 mmol/g).

Peptides were analysed by HPLC using an Agilent 1290 affinity LC system equipped with an Ascentis Express 10 cm × 2.1 mm, 2.7 µm ES-C18 peptide column (0.5 ml min^{-1}) and ultraviolet (UV) detection at 220-280 nm. The peptide column was run with a gradient from 0.1% TFA/5% MeCN (*v/v*) in H_2O to 0.1% TFA/95% MeCN (*v/v*) in H_2O over 10 minutes.

General procedure for Solid Phase Glycopeptide Synthesis

Low loading Rink Amide MBHA resin (loading: 0.33-0.35 mmol/g, 100 µmol) was shaken in DMF (5 mL) for 2 hours, filtered and washed with DMF. **Fmoc deprotection** – resin bound peptide intermediates were shaken in 20% piperidine in DMF (3 × 5 mL × 3 min) and washed with DMF, CH_2Cl_2 then DMF. **Couplings** – the resin was treated with a 5-fold excess of the Fmoc-amino acid (except for the Fmoc-Ser/Thr(GalNAc)-OH which was employed in a 2-fold excess) in 2 mL DMF containing HCTU (5 equiv.) and DIPEA (10 equiv). The mixture was shaken and coupling times were 40 mins (except for the Fmoc-Ser/Thr(GalNAc)-OH and the subsequent coupling each of which were 12 hours in duration). The excess reagents removed by filtration and the resin washed with DMF, CH_2Cl_2 then DMF. ***O*-Acetyl Deprotection** – Following coupling of the final amino acid, resin was washed with methanol, shaken in 70% hydrazine hydrate in methanol (3 × 5 mL × 5 min) and washed with methanol. **Glycopeptide Cleavage** – Following Fmoc and O-Acetyl deprotection, the resin was washed with CH_2Cl_2 and then methanol, before drying under vacuum. The resin was suspended in TFA:H_2O:TIS (95:2.5:2.5, 5 mL) and shaken for a maximum of 2 hours. The mixture was filtered and washed with TFA. The solution was concentrated under a stream of N_2 to <1 mL. Glycopeptide obtained by ether precipitation, isolated by centrifugation and pellet further washed with ether. The white precipitate was dissolved in H_2O and lyophilized to give white foams.

Synthesised Peptides & Glycopeptides

*H$_2$N-GVAPDTRP-CONH$_2$ (**1**)*

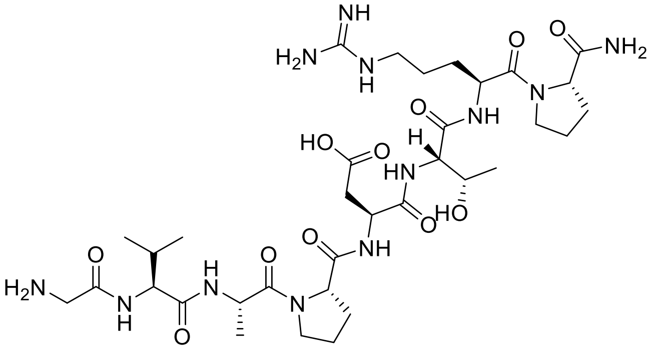

Yield: 30 mg, 58%.

HRMS [ES+] found $[M+H]^+$ 811.4433, $C_{34}H_{59}N_{12}O_{11}$ requires 811.4421; **LCMS** [ES+] found $[M+H]^+$ 811.55, $C_{34}H_{59}N_{12}O_{11}$ requires 811.44.

*H$_2$N-GVAPDT(GalNAc)RP-CONH$_2$ (**2**)*

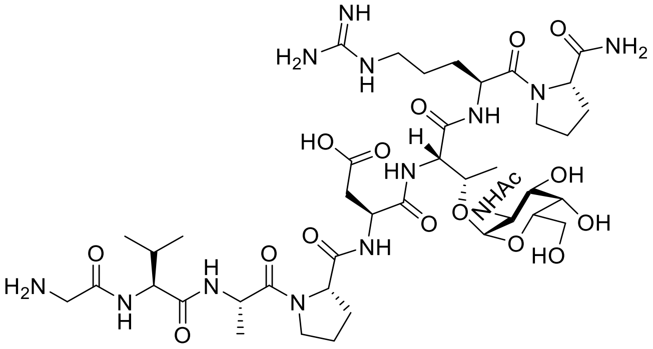

Yield: 28 mg, 41%.

HRMS [ES+] found $[M+H]^+$ 1014.5224, $C_{40}H_{71}N_{13}O_{16}$ requires 1014.5214; **LCMS** [ES+] found $[M+H]^+$ 1014.63, $C_{40}H_{71}N_{13}O_{16}$ requires 1014.52.

*H$_2$N-GVAPGSTAPPA-CONH$_2$ (**3**)*

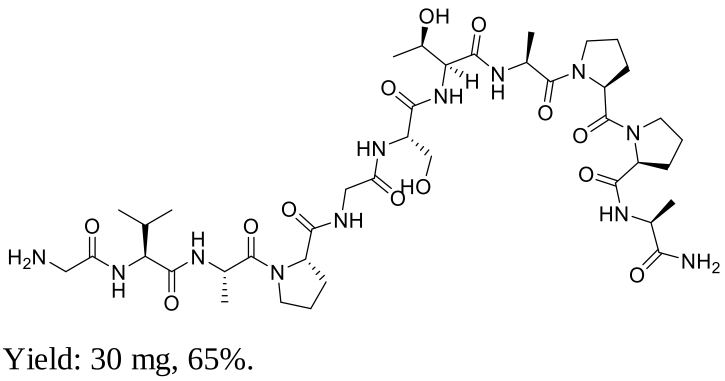

Yield: 30 mg, 65%.

HRMS [ES+] found $[M+H]^+$ 923.4960, $C_{40}H_{66}N_{12}O_{13}$ requires 923.4945; **LCMS** [ES+] found $[M+H]^+$ 923.61, $C_{40}H_{66}N_{12}O_{13}$ requires 923.49.

H₂N-GVAPGST(GalNAc)APPA-CONH₂ ***(4)***

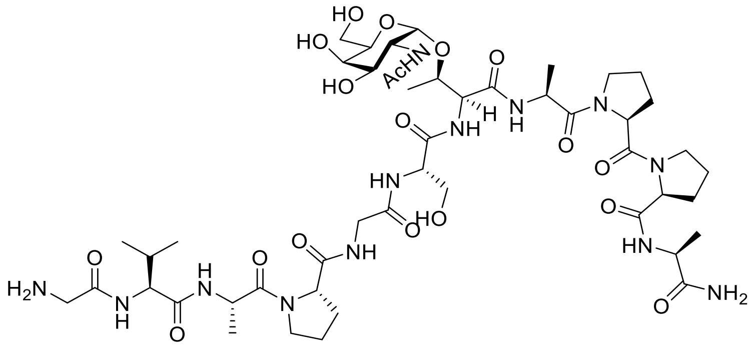

Yield: 45 mg, 80%.

HRMS [ES+] found $[M+H]^+$ 1126.5759, $C_{48}H_{79}N_{13}O_{18}$ requires 1126.5739; **LCMS** [ES+] found $[M+H]^+$ 1126.69, $C_{48}H_{79}N_{13}O_{18}$ requires 1126.57.

H₂N-GVAPGS(GalNAc)TAPPA-CONH₂ ***(5)***

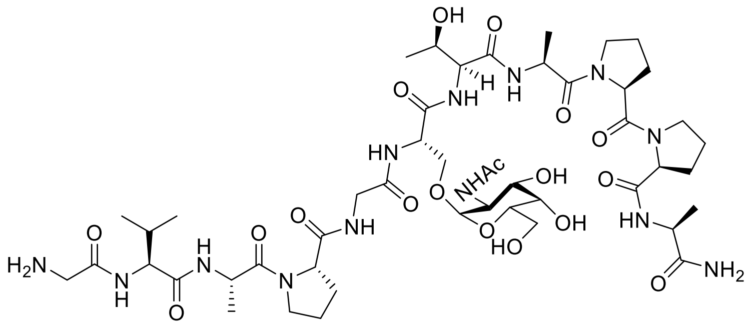

Yield: 47 mg, 75%.

HRMS [ES+] found $[M+H]^+$ 1126.5765, $C_{48}H_{79}N_{13}O_{18}$ requires 1126.5739; **LCMS** [ES+] found $[M+H]^+$ 1126.70, $C_{48}H_{79}N_{13}O_{18}$ requires 1126.57.

H₂N-GVAPGS(GalNAc)T(GalNAc)APPA-CONH₂ ***(6)***

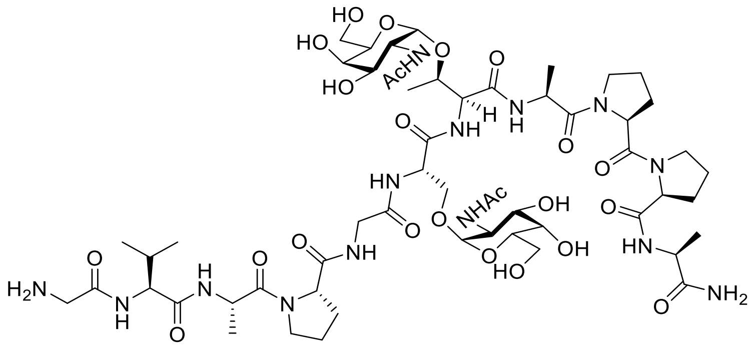

Yield: 40 mg, 55%.

HRMS [ES+] found $[M+H]^+$ 1329.6536, $C_{56}H_{92}N_{14}O_{23}$ requires 1329.6533; **LCMS** [ES+] found $[M+H]^+$ 1329.78, $C_{56}H_{92}N_{14}O_{23}$ requires 1329.65.

*H_2N-GVAADTRP-$CONH_2$ (**7**)*

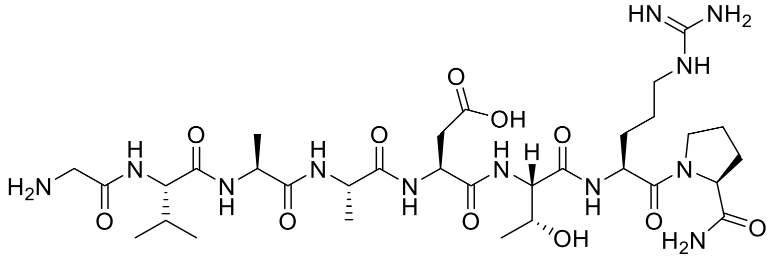

Yield: 32 mg, 82%.

HRMS [ES+] found $[M+H]^+$ 785.4274, $C_{32}H_{57}N_{12}O_{11}$ requires 785.4264; **LCMS** [ES+] found $[M+H]^+$ 785.41, $C_{32}H_{57}N_{12}O_{11}$ requires 785.43.

*H_2N-GVAADT(GalNAc)RP-$CONH_2$ (**8**)*

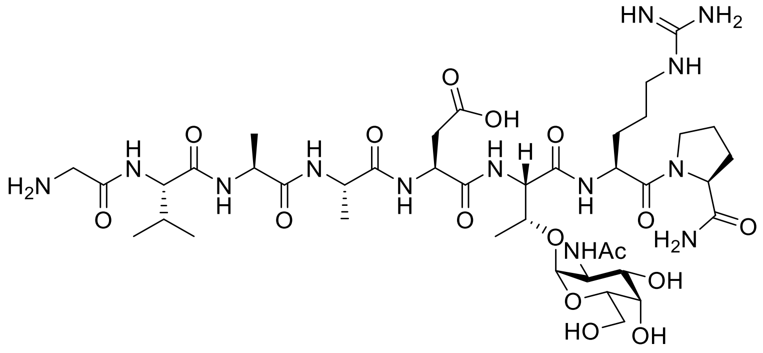

Yield: 58 mg, 77%.

HRMS [ES+] found $[M+H]^+$ 988.5061, $C_{40}H_{70}N_{13}O_{16}$ requires 988.5058; **LCMS** [ES+] found $[M+H]^+$ 988.50, $C_{40}H_{70}N_{13}O_{16}$ requires 988.51.

*H_2N-GV(AEEAc)APDT(GalNAc)RP-$CONH_2$ (**9**)*

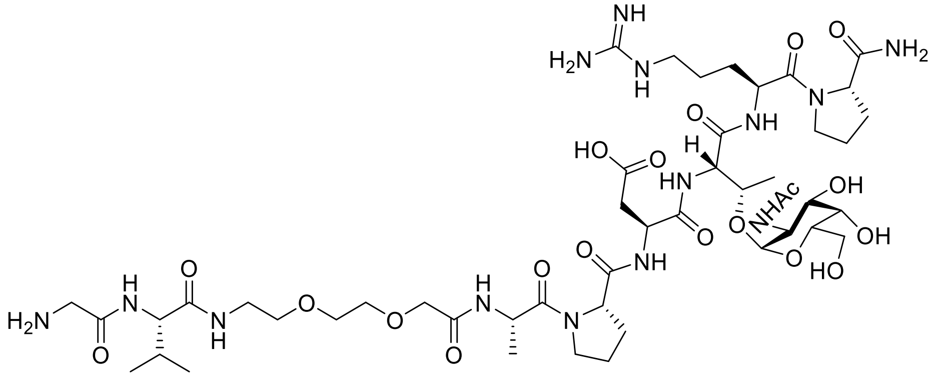

Yield: 41 mg, 71%.

HRMS [ES+] found $[M+H]^+$ 1159.5946, $C_{48}H_{83}N_{14}O_{19}$ requires 1159.5953; **LCMS** [ES+] found $[M+H]^+$ 1160.12, $C_{48}H_{83}N_{14}O_{19}$ requires 1159.60.

*H_2N-GV(AEEAc)$_2$APDT(GalNAc)RP-CONH$_2$ (**10**)*

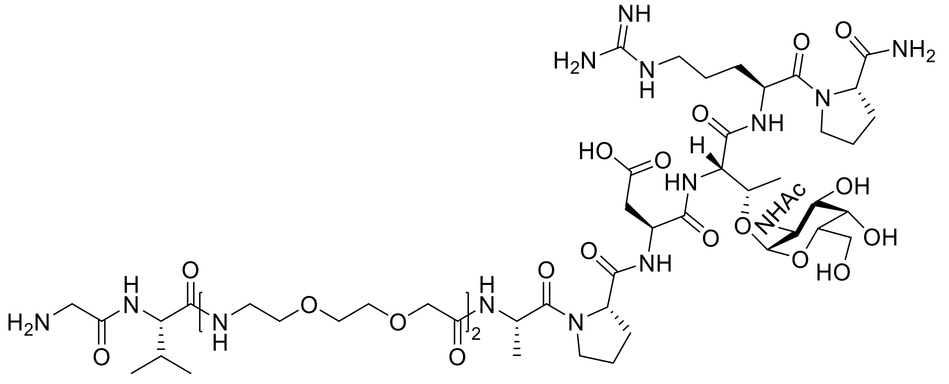

Yield: 42 mg, 64%.

HRMS [ES+] found $[M+H]^+$ 1304.6702, $C_{54}H_{94}N_{15}O_{22}$ requires 1304.6692; **LCMS** [ES+] found $[M+H]^+$ 1305.24, $C_{54}H_{94}N_{15}O_{22}$ requires 1304.67.

*H_2N-GV(AEEAc)$_3$APDT(GalNAc)RP-CONH$_2$ (**11**)*

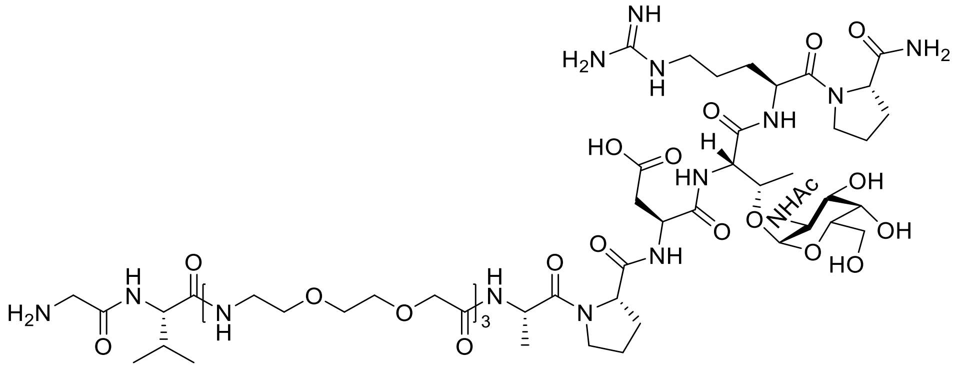

Yield: 50 mg, 69%.

HRMS [ES+] found $[M+2H]^{2+}$ 725.3766, $C_{60}H_{106}N_{16}O_{25}$ requires 1450.7504; **LCMS** [ES+] found $[M+2H]^{2+}$ 725.76, $C_{60}H_{106}N_{16}O_{25}$ requires 1450.75.

*H_2N-GGGAPDT(GalNAc)RP-CONH$_2$ (**12**)*

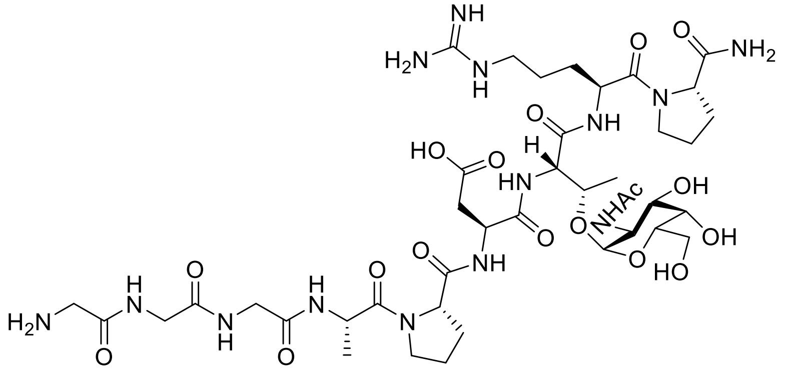

Yield: 48 mg, 93%.

HRMS [ES+] found $[M+H]^+$ 1029.4956, $C_{41}H_{69}N_{14}O_{17}$ requires 1029.4960; **LCMS** [ES+] found $[M+H]^+$ 1030.04, $C_{41}H_{69}N_{14}O_{17}$ requires 1029.50.

*H$_2$N-GV(PEG)$_6$APGSTAPPA-CONH$_2$ (**13**)*

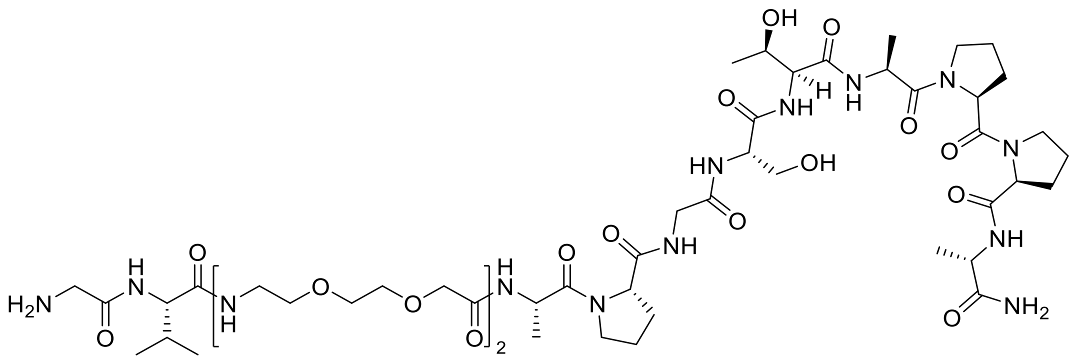

Yield: 42 mg, 84%.

HRMS [ES+] found $[M+H]^+$ 1213.6423, $C_{52}H_{89}N_{14}O_{19}$ requires 1213.6423; **LCMS** [ES+] found $[M+H]^+$ 1214.29, $C_{52}H_{89}N_{14}O_{19}$ requires 1213.64.

*H$_2$N-GV(PEG)$_6$APGST(GalNAc)APPA-CONH$_2$ (**14**)*

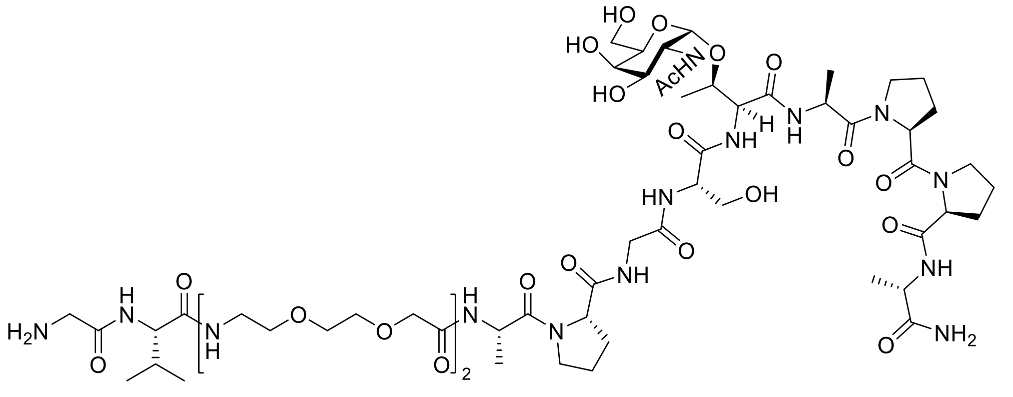

Yield: 48 mg, 76%.

HRMS [ES+] found $[M+H]^+$ 1416.7214, $C_{60}H_{102}N_{15}O_{24}$ requires 1416.7217; **LCMS** [ES+] found $[M+H]^+$ 1417.40, $C_{60}H_{102}N_{15}O_{24}$ requires 1416.72.

*H$_2$N-GV(PEG)$_6$APGS(GalNAc)TAPPA-CONH$_2$ (**15**)*

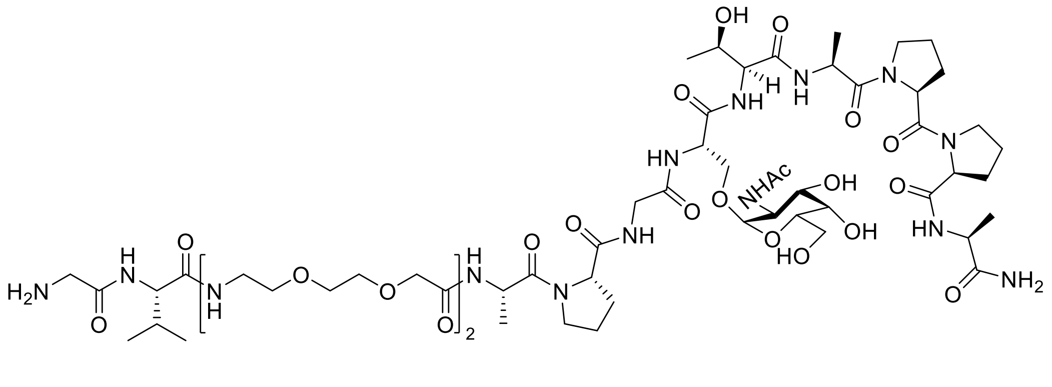

Yield: 43 mg, 68%.

HRMS [ES+] found $[M+H]^+$ 1416.7222, $C_{60}H_{102}N_{15}O_{24}$ requires 1416.7217; **LCMS** [ES+] found $[M+H]^+$ 1417.42, $C_{60}H_{102}N_{15}O_{24}$ requires 1416.72.

*H2N-GV(PEG)6APGS(GalNAc)T(GalNAc)APPA-CONH2 (**16**)*

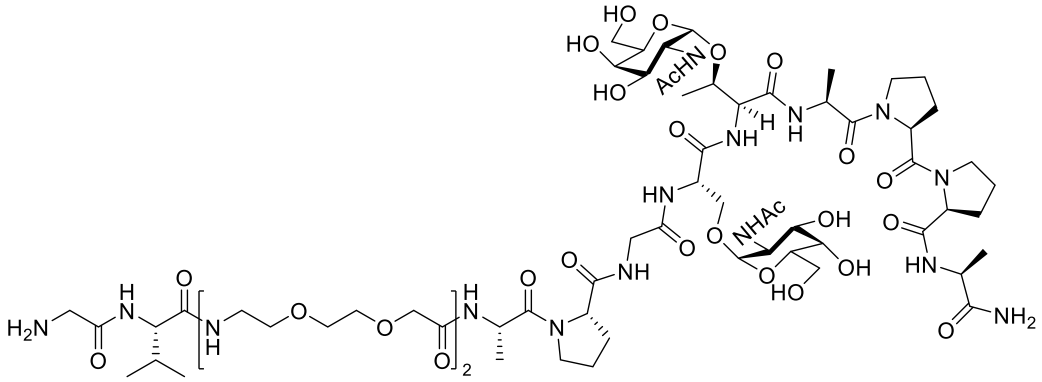

Yield: 55 mg, 75%.

HRMS [ES+] found $[M+2H]^{2+}$ 810.4036, $C_{68}H_{116}N_{16}O_{29}$ requires 1620.8094; **LCMS** [ES+] found $[M+H]^+$ 1620.57, $C_{68}H_{115}N_{16}O_{29}$ requires 1619.80.

*H2N-GV(PEG)6APPATSGPA-CONH2 (**17**)*

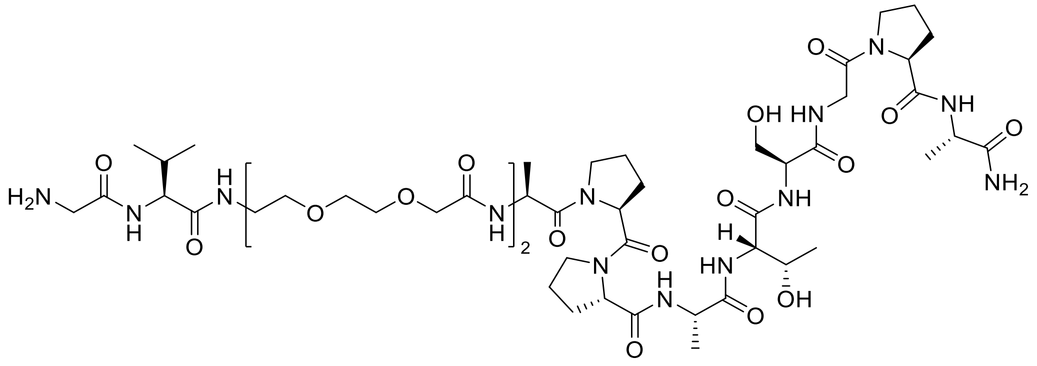

Yield: 41 mg, 77%.

HRMS [ES+] found $[M+H]^+$ 1213.6418, $C_{52}H_{89}N_{14}O_{19}$ requires 1213.6423; **LCMS** [ES+] found $[M+H]^+$ 1214.31, $C_{52}H_{89}N_{14}O_{19}$ requires 1213.64.

*H_2N-GV(PEG)$_6$APDTRP-CONH$_2$ (**18**)*

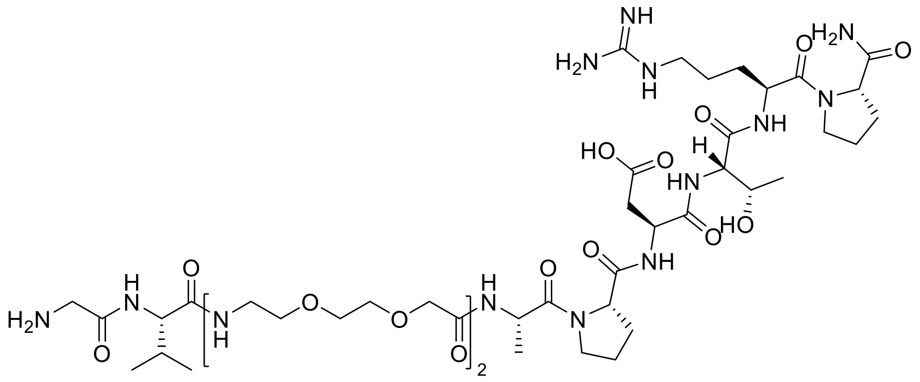

Yield: 37 mg, 77%.

HRMS [ES+] found $[M+H]^+$ 1101.5903, $C_{46}H_{81}N_{14}O_{17}$ requires 1101.5899; **LCMS** [ES+] found $[M+H]^+$ 1102.20, $C_{46}H_{81}N_{14}O_{17}$ requires 1101.59.

General Biochemical Methods and Equipment

Sterilisation of media and equipment was carried out in either a Prestige Medical bench top autoclave or an LTE Touchclave-R autoclave at 121 ºC for 15 minutes. Thermo Electron Corporation SAFE 2010 Class II laminar flow cabinet or bench top bunsen burner were using to maintain a sterile environment. Bacterial cultures were incubated in a Kuhner ShakerX ISF1-X or Stuart Orbital incubator. LB-agar plates and enzymatic reactions were incubated in a Binder BD23 incubator.

Centrifugation was performed using either a Beckman Coulter™ Avanti™ JXN-30 centrifuge, Heraeus mutifuge 3S-R centrifuge or Heraeus pico centrifuge. Gel filtration chromatography was achieved using a GE Pharmacia ÄKTA FPLC system or BioRad NGC FPLC system. Spectrophotometric readings were recorded using a Thermoscientific Nanodrop 2000. SDS-PAGE was carried out using Bio-Rad mini protean 3 apparatus, and a Bio-Rad imager Gel Doc™ XR was used to visualise polyacrylamide gels. Proteins were concentrated either using 10k or 30k MWCO Amicon® Ultra-15 centrifugal filter device or Amicon® Stirred Ultrafiltration Cell (10k MWCO membrane). High resolution mass spectrometry of protein samples was performed using a Bruker Daltonics MicroTOF mass spectrometer. Protein samples were loaded at a concentration of 20-40 µM (made up with H_2O) into the instrument before being automatically diluted into 0.1% TFA/50% MeCN (*v/v*) in H_2O prior to analysis. Analytical grade reagents were supplied by commercial suppliers.

Media & Buffers

Buffers and media were made in-house unless otherwise stated using analytical grade reagents supplied by commercial suppliers. All common buffers and media were prepared with 18.2 MΩ water to the required volume. The pH of the solutions was adjusted using 5 M NaOH or 5 M HCl. Gel filtration buffers were filtered through a 0.22 µm membrane under reduced pressure. Media was sterilised by autoclave at 121 °C for 20 minutes.

Millers lysogeny broth (LB) medium: 1.0% (*w/v*) Tryptone, 0.5% (*w/v*) Yeast Extract, 1.0% (*w/v*) NaCl.

Auto induction medium: 1.0% (*w/v*) Tryptone, 0.5% (*w/v*) Yeast Extract, 4% (*v/v*) 25 × salts, 0.1% (*v/v*) 1000 × metals, 4.3 mM $MgCl_2$. Solution autoclaved at 120 °C for 20 mins before addition of 20 mL L^{-1} 50 × Sugar Mix by filter sterilisation (0.2 µm Sartorius Minisart).

> *25 × salts:* 1.25 mM Na_2HPO_4, 1.25 mM KH_2PO_4, 2.5 mM NH_4Cl, 250 mM Na_2SO_4, pH 7.4.
>
> *1000 × metals:* 50 mM $FeCl_3{\cdot}6H_2O$, 20 mM $CaCl_2{\cdot}6H_2O$, 100 mM $MnCl_2{\cdot}4H_2O$, 100 mM $ZnSO_4{\cdot}7H_2O$, 1.7 mM $CoCl_2{\cdot}6H_2O$, 2 mM $CuCl_2{\cdot}2H_2O$, 4.1 mM $Na_2MoO_4{\cdot}2H_2O$, 2 mM Na_2SeO_3, 2 mM H_3BO_4, 2 mM $NiSO_4{\cdot}6H_2O$, 50% (*v/v*) 0.1 M HCl.
>
> *50 × Sugar Mix:* 20% (*v/v*) glycerol, 2.5% (*w/v*) glucose, 10% (*w/v*) lactose.

Sodium Phosphate Buffered Saline: 50 mM NaH_2PO_4, 100 mM NaCl, pH 7.4.

Tris Buffered Saline: 50 mM Tris, 150 mM NaCl, pH 7.2.

Expression of CTB-LPETGA

A glycerol stock of C41 cells containing the mutant plasmid pSAB2.2-LPETGA was used to inoculate 2 x 5 ml of LB media containing 100 µg/ml ampicillin. These cultures were incubated at 37 °C overnight, shaking at 200 rpm and then used to inoculate 4 x 1 L of LB media containing 100 µg/ml of ampicillin. The culture was incubated at 37 °C with shaking (200 rpm) and overexpression by the addition of IPTG (0.5 mM) when OD_{600} = 0.6-0.8 was reached. The cells were incubated overnight at a 25 °C before being collected by centrifugation (10,000 × g, 20 minutes, 4 °C). The *E. coli* cell pellet was discarded and media retained. Solid ammonium sulfate was added and dissolved to a final concentration of 57% (*w/v*) to precipitate the protein of interest. The saturated solution was stirred overnight at 4 °C. The solution was centrifuged (17,600 × g, 1 hour, 4 °C), and the supernatant discarded. The protein pellet was resuspended in sodium phosphate buffered saline (10 mL per 1 L LB media) and centrifuged (17,600 × g, 10 minutes, 4 °C) to remove insoluble material and to allow the suspension to be filtered through a 0.8 µm Sartorius Minisart filter.

The protein was purified by Ni-NTA affinity chromatography washing with sodium phosphate buffered saline (no imidazole). CTB has native surface histidine residue meaning the pentamer is able to bind to Ni-NTA resin without the presence of a His-tag. The protein was then eluted from the column by washing with sodium phosphate buffered saline containing 200 mM imidazole, before further purification by size exclusion chromatography on Superdex S75 in sodium phosphate buffered saline.

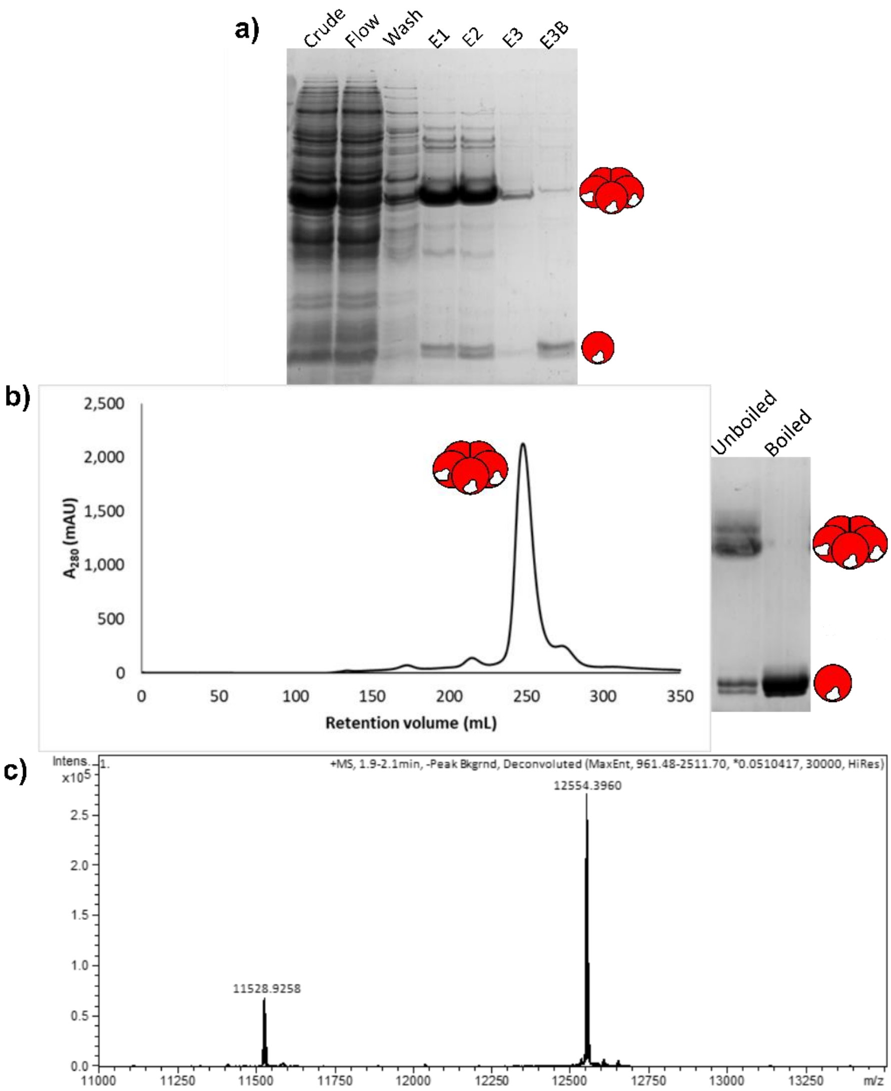

Figure S1: *Purification of CTB-LPETGA for use in labelling reactions. a) SDS-PAGE gel of Ni-NTA affinity chromatography flow through, wash and elution fractions (E1-3). b) SEC trace for CTB-LPETGA using Superdex® S200 16/60 with SDS-PAGE get showing formation of a stable pentamer which dissociates upon boiling. Two bands appear for protomeric CTB showing a mixture of full length and truncated material. c) Deconvoluted ES-MS of purified CTB-LPETGA protomer (12554.4 Da) and the truncated CTB protomer (11528.9 Da).*

Expression of Sortase 7M and CBD-Sortase 7M

The plasmid pet30b-7M SrtA was a gift from Hidde Ploegh (Addgene plasmid #51141; http://n2t.net/addgene:51141; RRID:Addgene_51141). The plasmid pET28a-CBD-Srt7M is a syntetic construct obtained from genscript encoding Sortase 7Mwith an N-terminal hexahistidine and chitin binding domain purification tags.

A glycerol stock of BL21(DE3) cells containing the mutant plasmid pET30b-Srt7M (Wuethrich et al., 2014) or pET28a-CBD-Srt7M was used to inoculate 5 ml of LB media containing 50 µg/ml kanamycin. These cultures were incubated at 37 °C overnight, shaking at 200 rpm and then 2 mL used to inoculate 1 L of autoinduction-LB medium (Studier, 2005) containing 100 µg/ml of kanamycin. The culture was incubated at 37 °C with shaking (200

rpm) for 5 hours then the incubator temperature was reduced to 25 °C. The cells were incubated overnight before being collected by centrifugation (13,000 × g, 20 minutes, 4 °C). Cells were either used immediately or stored at -80 °C for future lysis and purification.

E. coli cells were resuspended in Tris buffered saline at 4 °C and treated with DNase (10 µg/mL) and protease inhibitors. The cell mixture was lysed mechanically using a Constant Systems cell disruptor (20 kpsi). The cell debris was pelleted by centrifugation (30,000 × g, 45 mins, 4 °C) and the cell lysate was decanted off. Purification of the clarified lysate performed by Ni-NTA affinity chromatography washing with Tris buffered saline containing 50 mM imidazole. The protein was then eluted from the column by washing with Tris buffered saline containing 250 mM imidazole, before further purification by size exclusion chromatography on Superdex S75 in Tris buffered saline. Sortase 7M & CBD-Sortase 7M was stored at -80 °C containing 10% (*v/v*) glycerol.

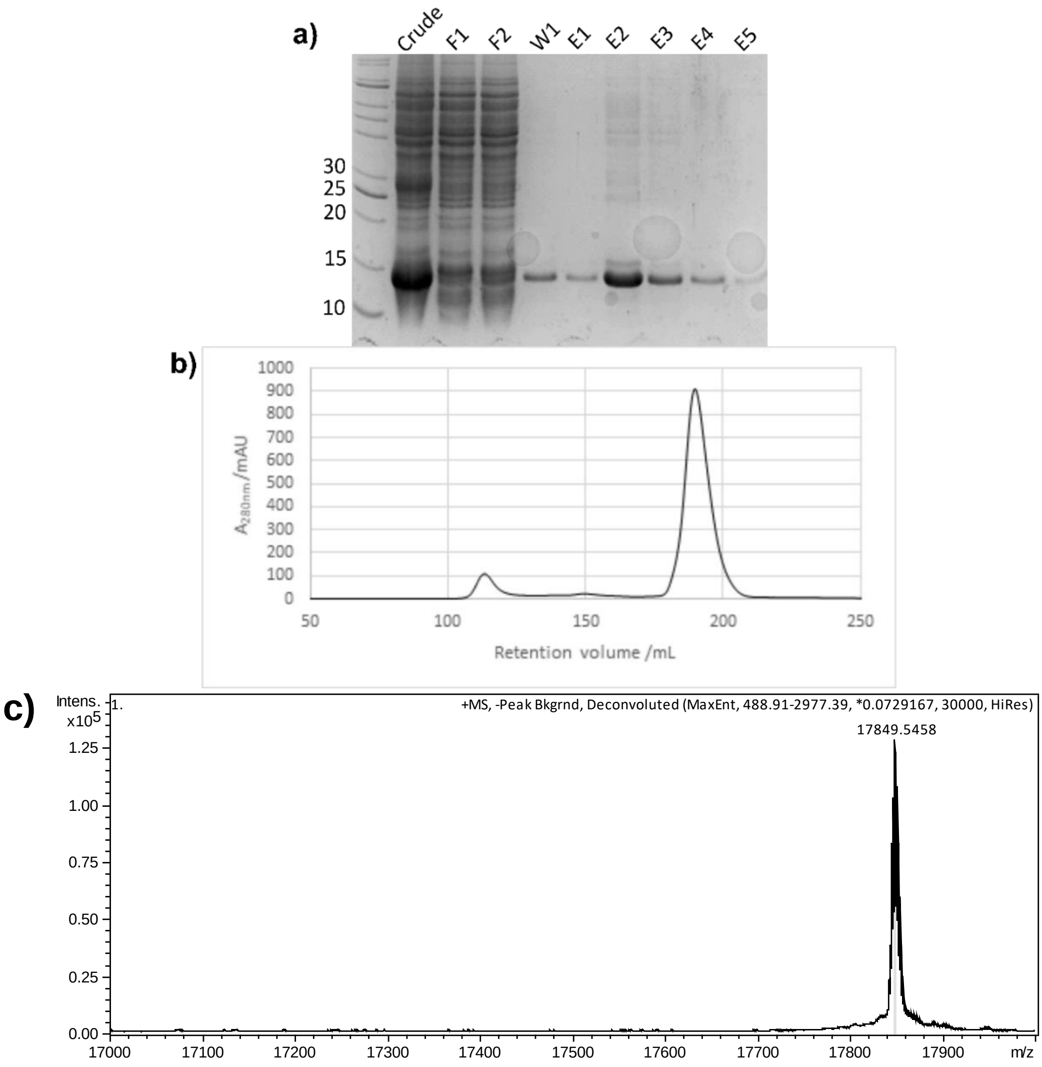

Figure S2: *Purification of Sortase 7M-His6 for use in labelling reactions. a) SDS-PAGE gel of Ni-NTA affinity chromatography column flow through (F1), wash (W1-2) and elution fractions (E1-3). b) SEC trace for Sortase 7M-His6 using Superdex® S75 26/60. c) Deconvoluted ES-MS of purified Sortase 7M-His6 (17849.5458 Da).*

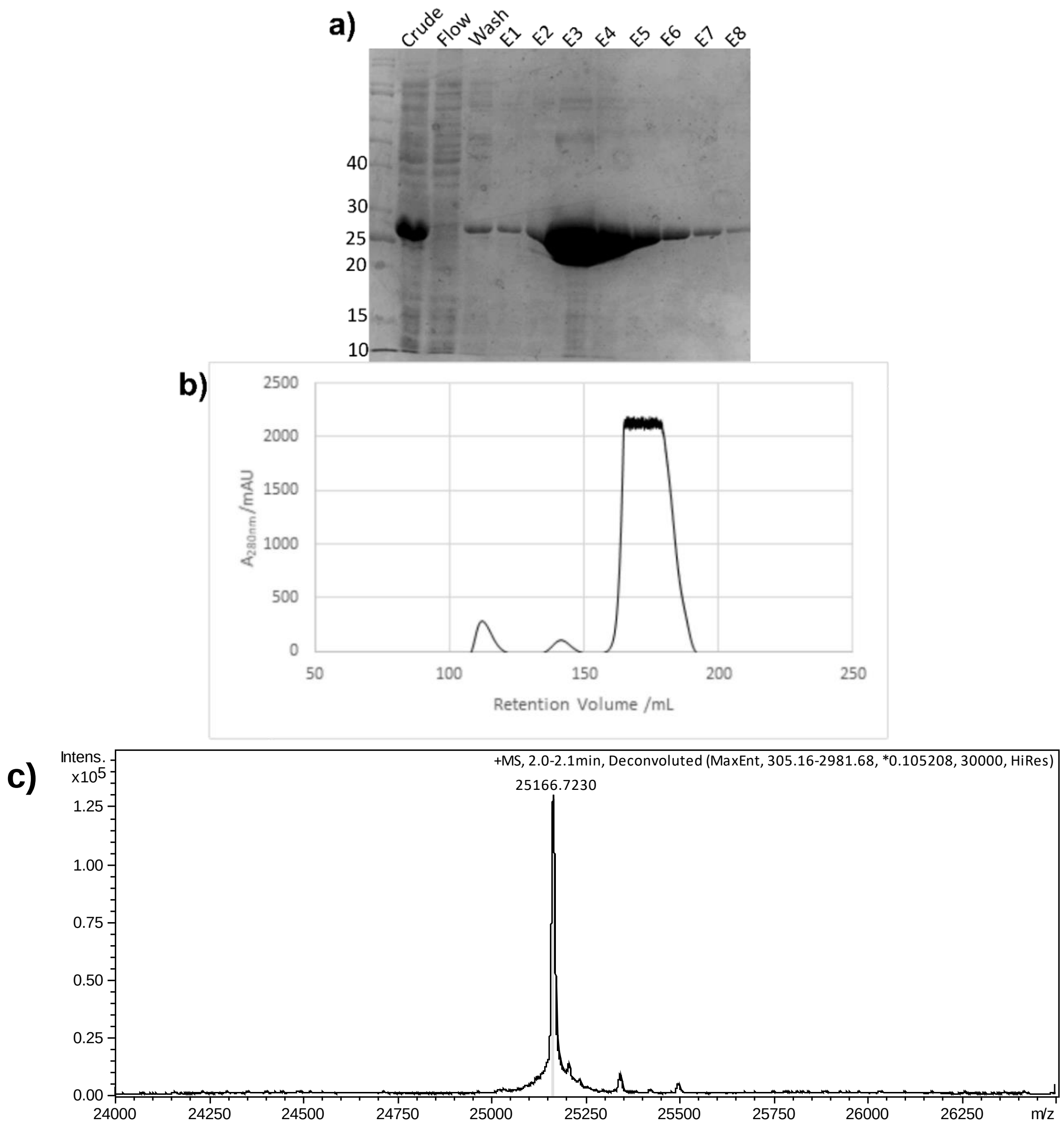

Figure S3: *Purification of Chitin-binding Sortase 7M for use in preparative scale ligation reactions. a) SDS-PAGE gel of Ni-NTA affinity chromatography column flow through, wash and elution fractions (E1-8). b) SEC trace for Chitin-binding Sortase 7M using Superdex® S75 26/60. c) Deconvoluted ES-MS of purified Chitin-binding Sortase 7M (25166.7230 Da).*

SDS-PAGE Gels of C-terminal Sortase Ligation Optimisation

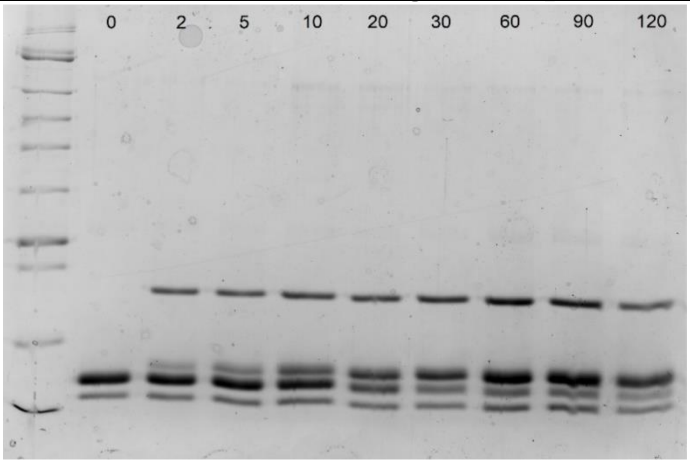

Figure S4: *SDS-PAGE gel showing sortase 7M ligation of CTB-LPETGA and Peptide* ***1*** *over 2 hours.*

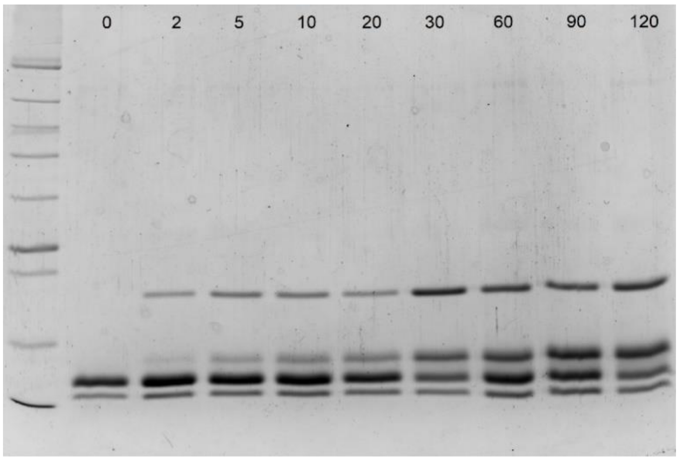

Figure S5: *SDS-PAGE gel showing sortase 7M ligation of CTB-LPETGA and Glycopeptide* ***2*** *over 2 hours.*

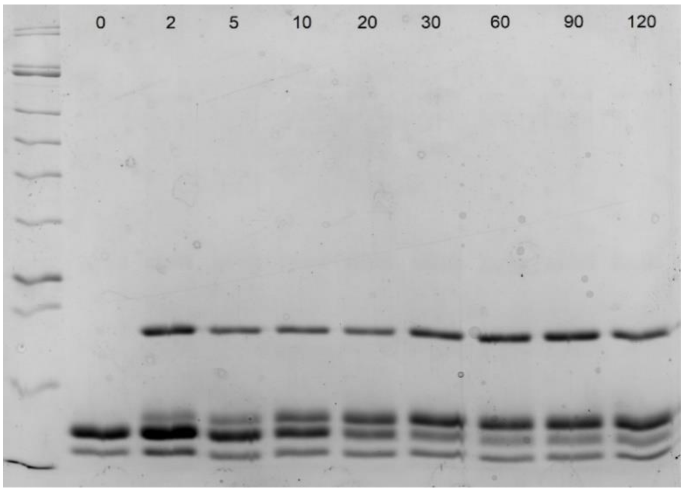

Figure S6: *SDS-PAGE gel showing sortase 7M ligation of CTB-LPETGA and Peptide **1** in the presence of 10% (v/v) DMSO over 2 hours.*

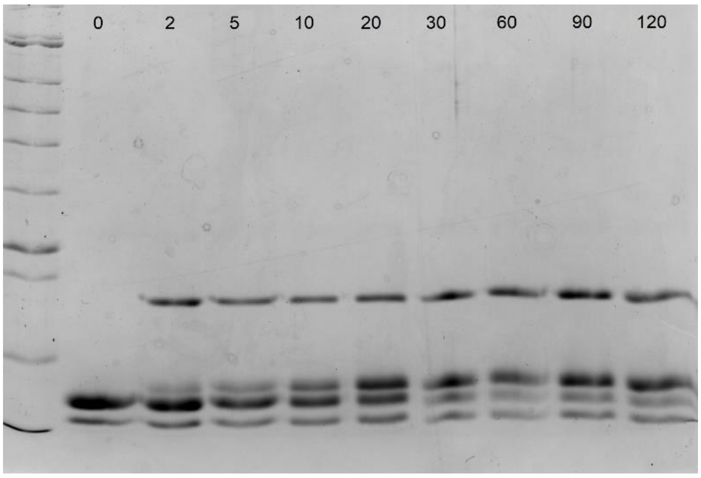

Figure S7: *SDS-PAGE gel showing sortase 7M ligation of CTB-LPETGA and Glycopeptide **2** in the presence of 10% (v/v) DMSO over 2 hours.*

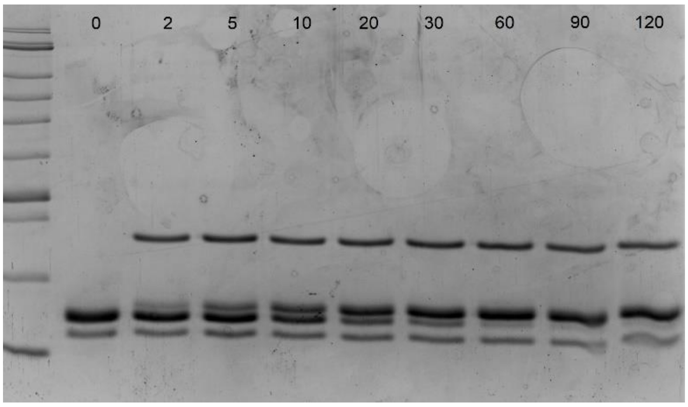

Figure S8: *SDS-PAGE gel showing sortase 7M ligation of CTB-LPETGA and Peptide **7** (P4A Variant of Peptide **1**) over 2 hours.*

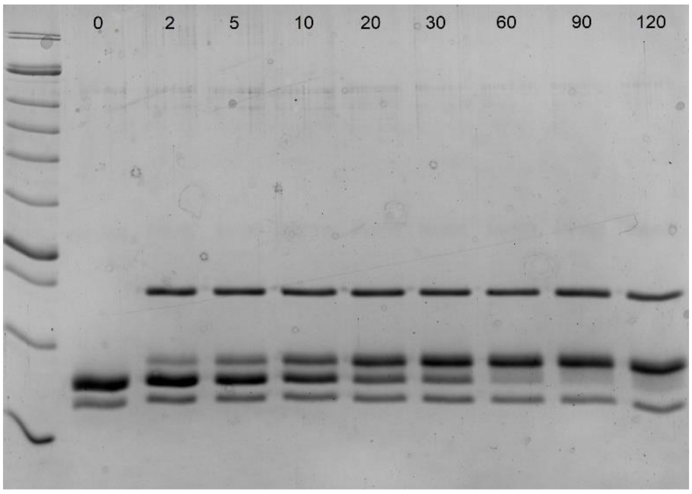

Figure S9: *SDS-PAGE gel showing sortase 7M ligation of CTB-LPETGA and Glycopeptide* ***8*** *(P4A Variant of Glycopeptide* ***2****) over 2 hours.*

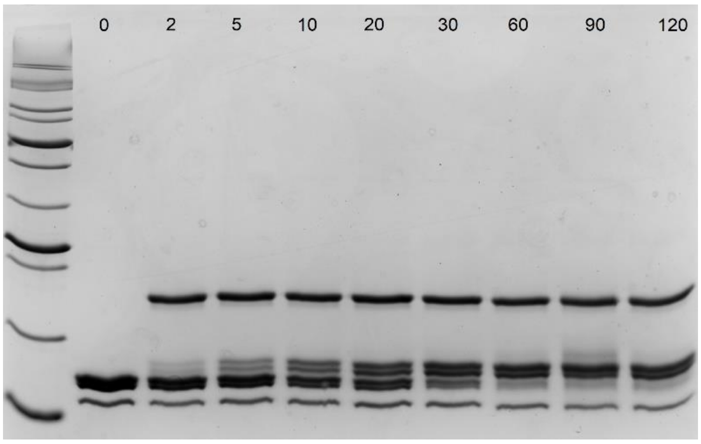

Figure S10: *SDS-PAGE gel showing sortase 7M ligation of CTB-LPETGA and Glycopeptide* ***9*** *in the presence of 10% (v/v) DMSO over 2 hours.*

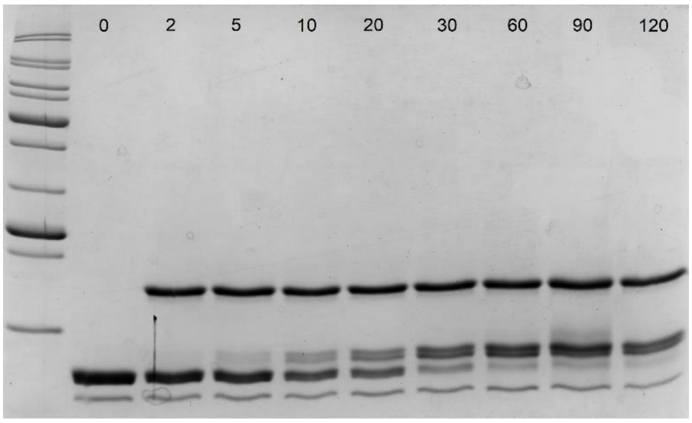

Figure S11: *SDS-PAGE gel showing sortase 7M ligation of CTB-LPETGA and Glycopeptide* ***10*** *in the presence of 10% (v/v) DMSO over 2 hours.*

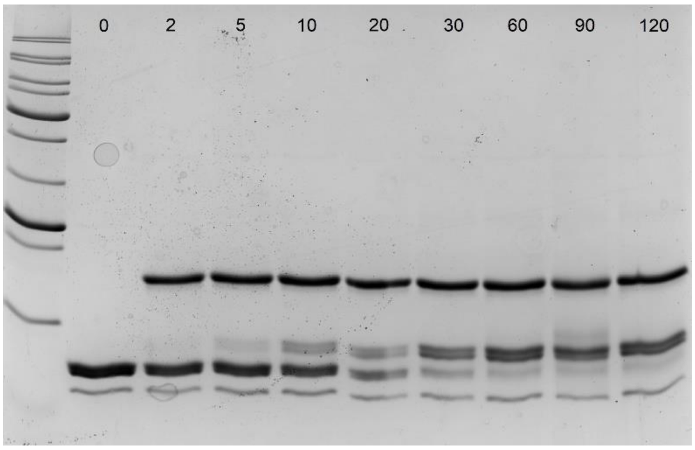

Figure S12: *SDS-PAGE gel showing sortase 7M ligation of CTB-LPETGA and Glycopeptide* ***11*** *in the presence of 10% (v/v) DMSO over 2 hours.*

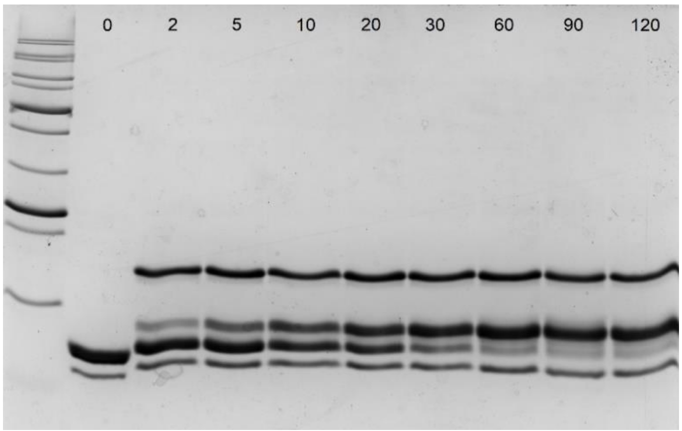

Figure S13: *SDS-PAGE gel showing sortase 7M ligation of CTB-LPETGA and Glycopeptide* ***12*** *in the presence of 10% (v/v) DMSO over 2 hours.*

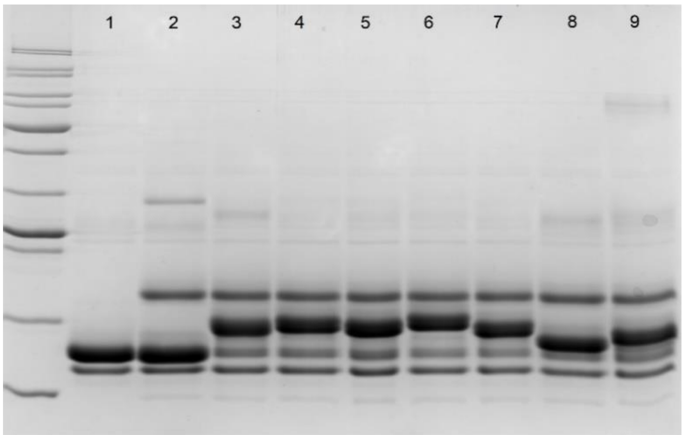

Figure S14: *SDS-PAGE gel showing sortase 7M ligation to CTB-LPETGA in the presence of 10% (v/v) DMSO after 2 hours. Lane 1: CTB-LPETGA (unlabelled); Lane 2: CTB-LPETGA (incubated with Sortase 7M); Lane 3: Glycopeptide* ***13****; Lane 4: Glycopeptide* ***14****; Lane 5:*

*Glycopeptide **15**; Lane 6: Glycopeptide **16**; Lane 7: Glycopeptide **17**; Lane 8: Glycopeptide **18**; Lane 9: Glycopeptide **10**.*

SDS-PAGE Gels of CBD-Sortase7M Comparison and Optimisation

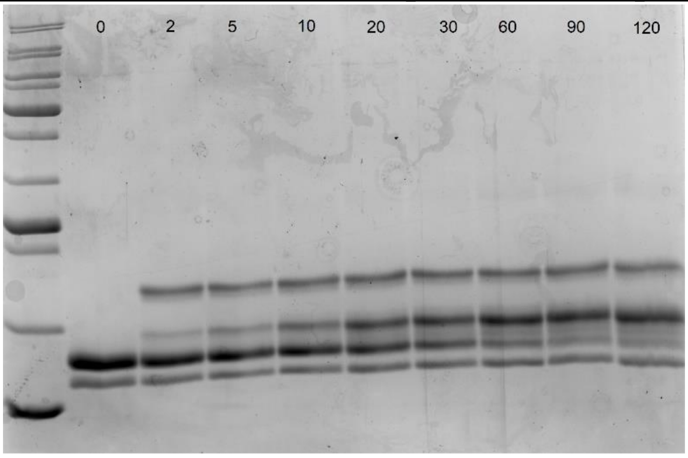

Figure S15: *SDS-PAGE gel showing Sortase 7M-His$_6$ ligation of CTB-LPETGA and Glycopeptide **10** in the presence of 10% (v/v) DMSO over 2 hours.*

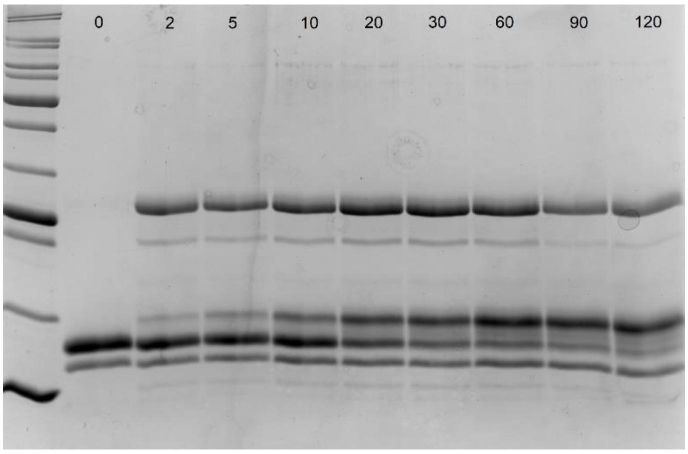

Figure S16: *SDS-PAGE gel showing His$_6$-CBD-Sortase 7M ligation of CTB-LPETGA and Glycopeptide **10** in the presence of 10% (v/v) DMSO over 2 hours.*

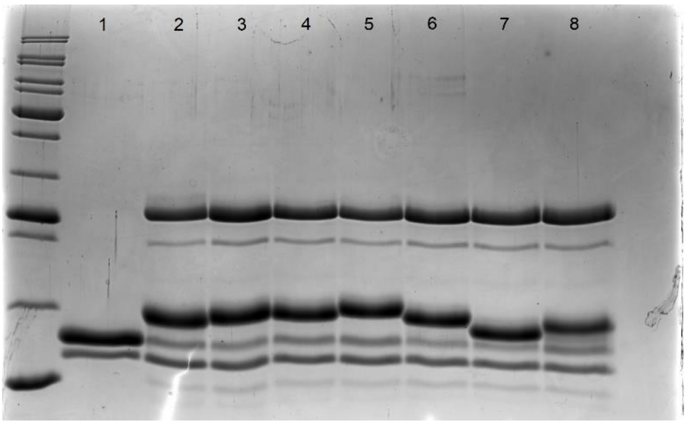

Figure S17: *SDS-PAGE gel showing His$_6$-CBD-Sortase 7M ligation to CTB-LPETGA in the presence of 10% (v/v) DMSO after 2 hours (Pre-purification of CBD-Sortase 7M with chitin resin and size-exclusion chromatography). Lane 1: CTB-LPETGA (unlabelled); Lane 2: CTB-Glycopeptide* ***13****; Lane 3: CTB-Glycopeptide* ***14****; Lane 4: CTB-Glycopeptide* ***15****; Lane 5: CTB-Glycopeptide* ***16****; Lane 6: CTB-Glycopeptide* ***17****; Lane 7: CTB-Glycopeptide* ***18****; Lane 8: CTB-Glycopeptide* ***10****.*

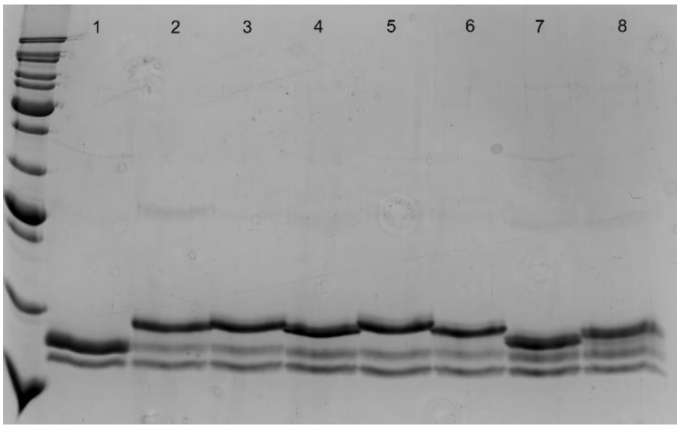

Figure S18: *SDS-PAGE gel showing His6-CBD-Sortase 7M ligation to CTB-LPETGA in the presence of 10% (v/v) DMSO after 2 hours (Post-purification of CBD-Sortase 7M with chitin resin and size-exclusion chromatography). Lane 1: CTB-LPETGA (unlabelled); Lane 2: CTB-Glycopeptide* ***13****; Lane 3: CTB-Glycopeptide* ***14****; Lane 4: CTB-Glycopeptide* ***15****; Lane 5: CTB-Glycopeptide* ***16****; Lane 6: CTB-Glycopeptide* ***17****; Lane 7: CTB-Glycopeptide* ***18****; Lane 8: CTB-Glycopeptide* ***10****.*

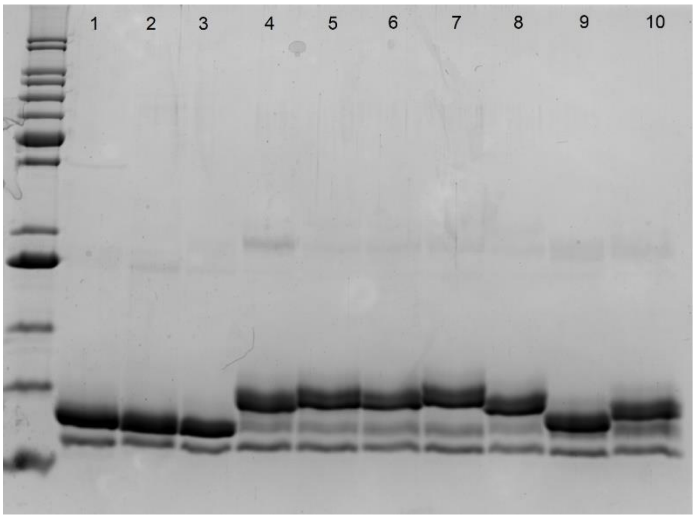

Figure S19: *SDS-PAGE gel showing His$_6$-CBD-Sortase 7M ligation to CTB-LPETGA in the presence of 10% (v/v) DMSO after 2 hours (Post-purification of CBD-Sortase 7M with chitin resin and size-exclusion chromatography). Lane 1: CTB-LPETGA (unlabelled); Lane 2: CTB-LPETGA (oxidised); Lane 3: Biotin-CTB-LPETGA; Lane 4: Biotin-CTB-Glycopeptide* ***13****; Lane 5: Biotin-CTB-Glycopeptide* ***14****; Lane 6: Biotin-CTB-Glycopeptide* ***15****; Lane 7: Biotin-CTB-Glycopeptide* ***16****; Lane 8: Biotin-CTB-Glycopeptide* ***17****; Lane 9: Biotin-CTB-Glycopeptide* ***18****; Lane 10: Biotin-CTB-Glycopeptide* ***10****.*

Protein HRMS Spectra of Oxime Ligation

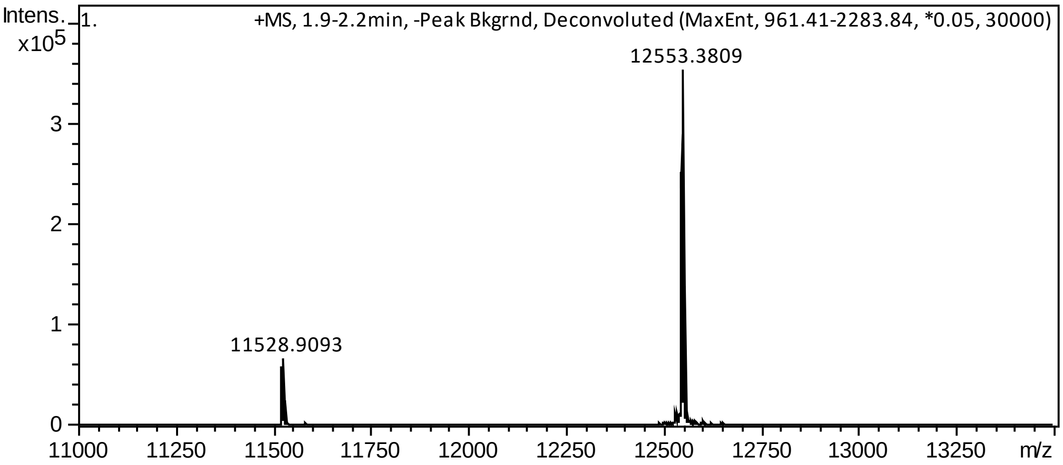

Figure S20: *Deconvoluted high resolution protein mass spectrometry of CTB-LPETGA (12553.3809 Da) and truncated CTB (11528.9093 Da) before oxidation with NaIO$_4$.*

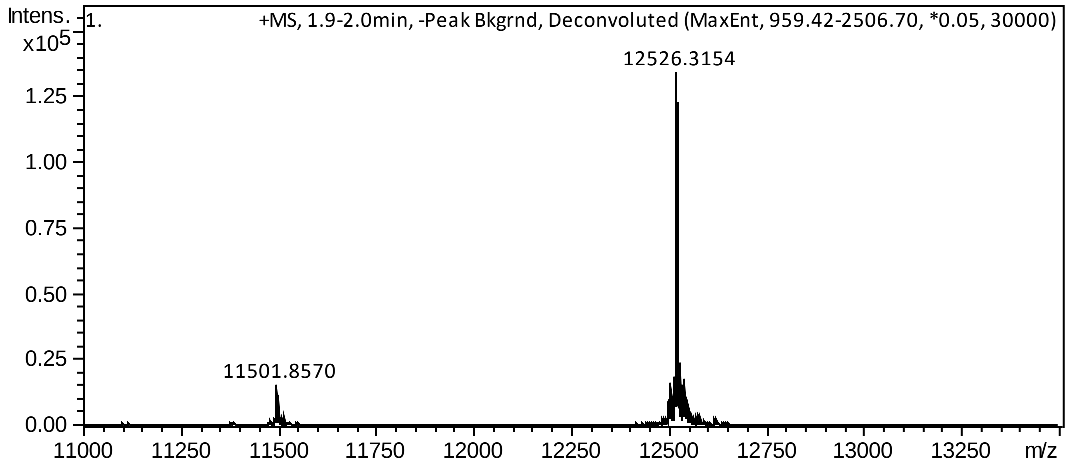

Figure S21: *Deconvoluted high resolution protein mass spectrometry of $NaIO_4$ oxidation reaction after 10 minutes showing quantitative oxidation of both substrates in their hydrate forms.*

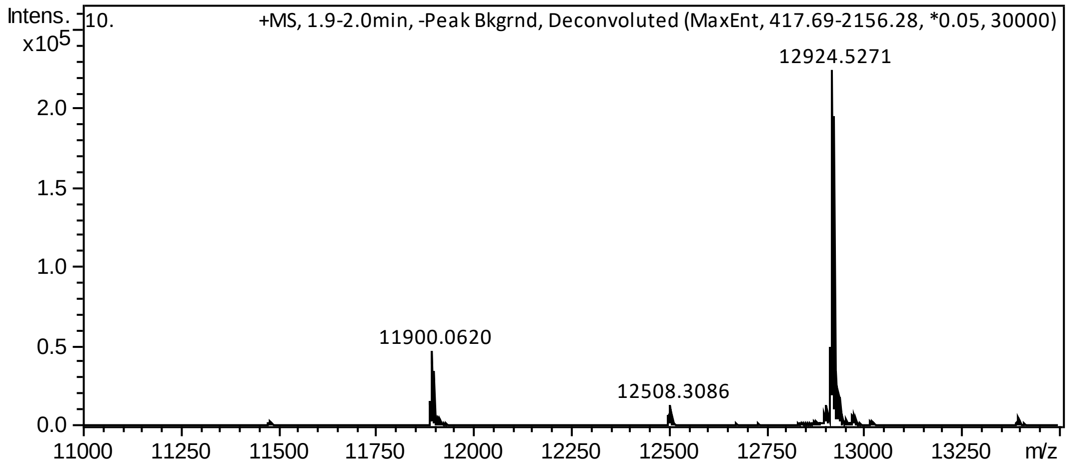

Figure S22: *Deconvoluted high resolution protein mass spectrometry of oxime ligation reaction with alkoxyamine-PEG_4-biotin, showing near quantitative N-terminal biotinylation of both truncated CTB (11900.0620 Da) and CTB-LPETGA (12924.5271 Da). A small amount of oxidized-CTB-LPETGA that has cyclized to a diketopiperidine is present at 12508.3086 Da.*

References

Alper, P. B., Hung, S.-C., and Wong, C.-H. (1996). Metal catalyzed diazo transfer for the synthesis of azides from amines. *Tetrahedron Lett.* 37, 6029–6032. doi:10.1016/0040-4039(96)01307-X.

De Silva, R. A., Wang, Q., Chidley, T., Appulage, D. K., and Andreana, P. R. (2009). Immunological response from an entirely carbohydrate antigen: Design of synthetic vaccines based on Tn-PS A1 conjugates. *J. Am. Chem. Soc.* 131, 9622–9623. doi:10.1021/ja902607a.

Fulmer, G. R., Miller, A. J. M., Sherden, N. H., Gottlieb, H. E., Nudelman, A., Stoltz, B. M., et al. (2010). NMR Chemical Shifts of Trace Impurities: Common Laboratory Solvents, Organics, and Gases in Deuterated Solvents Relevant to the Organometallic Chemist. *Organometallics* 29, 2176–2179. doi:10.1021/om100106e.

Koeller, K. M., Smith, M. E. B., and Wong, C. H. (2000). Chemoenzymatic synthesis of PSGL-1 glycopeptides: Sulfation on tyrosine affects glycosyltransferase-catalyzed synthesis of the O-glycan. *Bioorganic Med. Chem.* 8, 1017–1025. doi:10.1016/S0968-0896(00)00041-9.

Potter, G. T., Jayson, G. C., Miller, G. J., and Gardiner, J. M. (2016). An Updated Synthesis of the Diazo-Transfer Reagent Imidazole-1-sulfonyl Azide Hydrogen Sulfate. *J. Org. Chem.* 81, 3443–3446. doi:10.1021/acs.joc.6b00177.

Studier, F. W. (2005). Protein production by auto-induction in high-density shaking cultures. *Protein Expr. Purif.* 41, 207–234. doi:10.1016/j.pep.2005.01.016.

Wuethrich, I., Peeters, J. G. C., Blom, A. E. M., Theile, C. S., Li, Z., Spooner, E., et al. (2014). Site-Specific Chemoenzymatic Labeling of Aerolysin Enables the Identification of New Aerolysin Receptors. *PLoS One* 9, e109883. doi:10.1371/journal.pone.0109883.

NMR Spectra

*Imidazole-1-sulfonyl azide hydrogen sulphate salt (**S1**)*

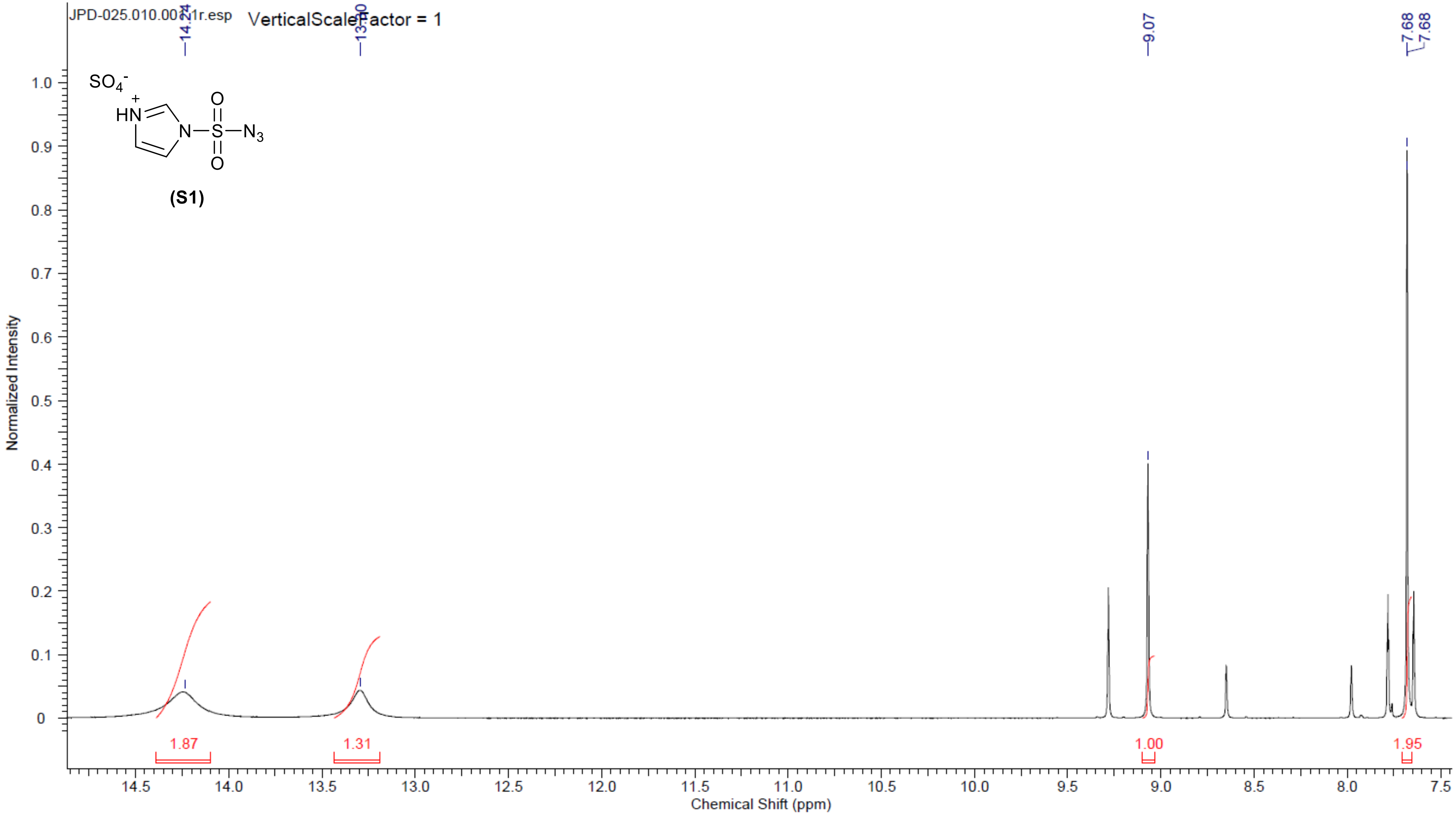

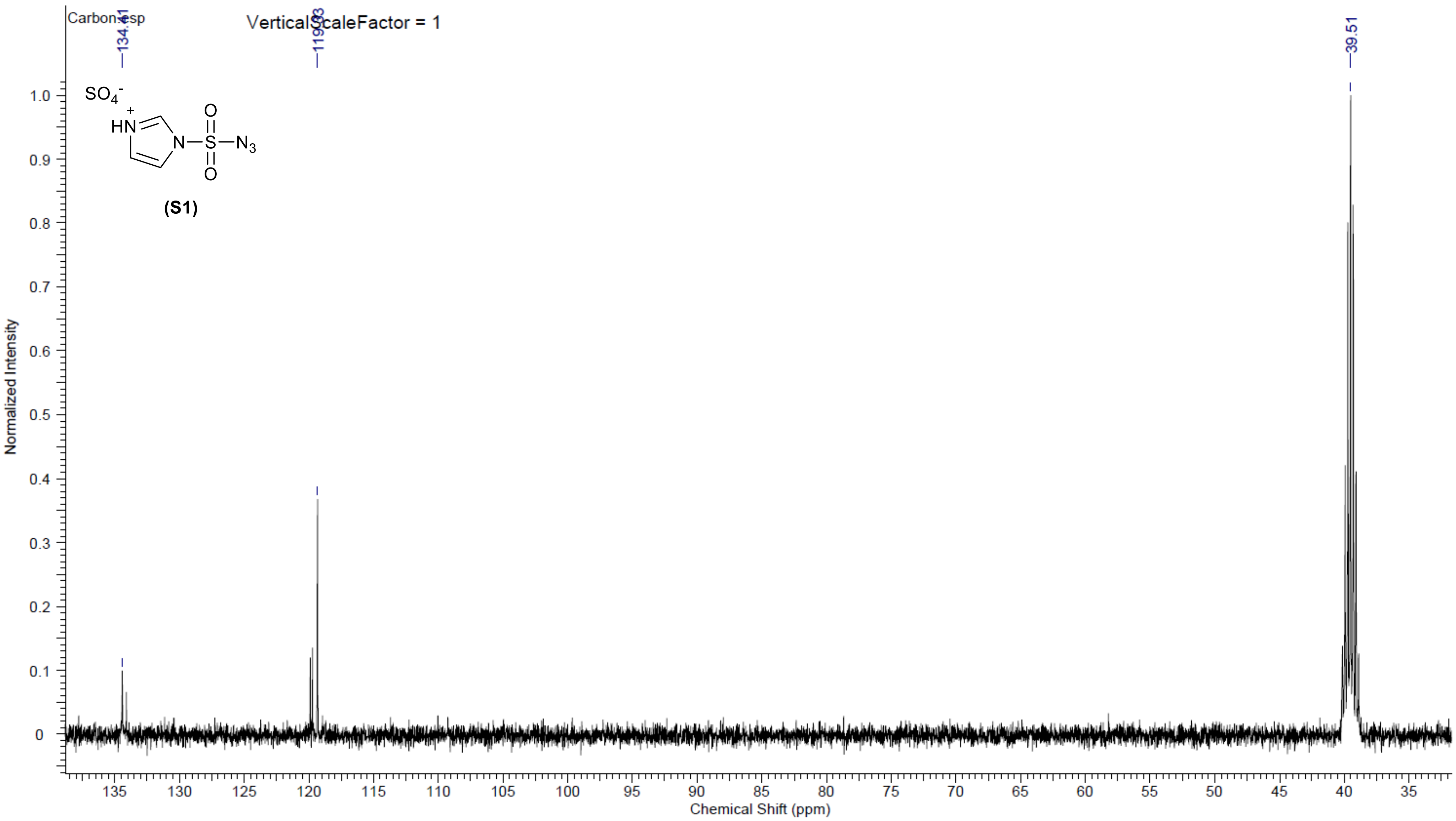

Carbon.esp
VerticalScaleFactor = 1
134.41
119.43
39.51
SO4-
HN+
N—S—N3
O
O
(S1)
Normalized Intensity
Chemical Shift (ppm)

*1,3,4,6-Tetra-O-acetyl-2-azido-2-deoxy-D-galactose (**S2**)*

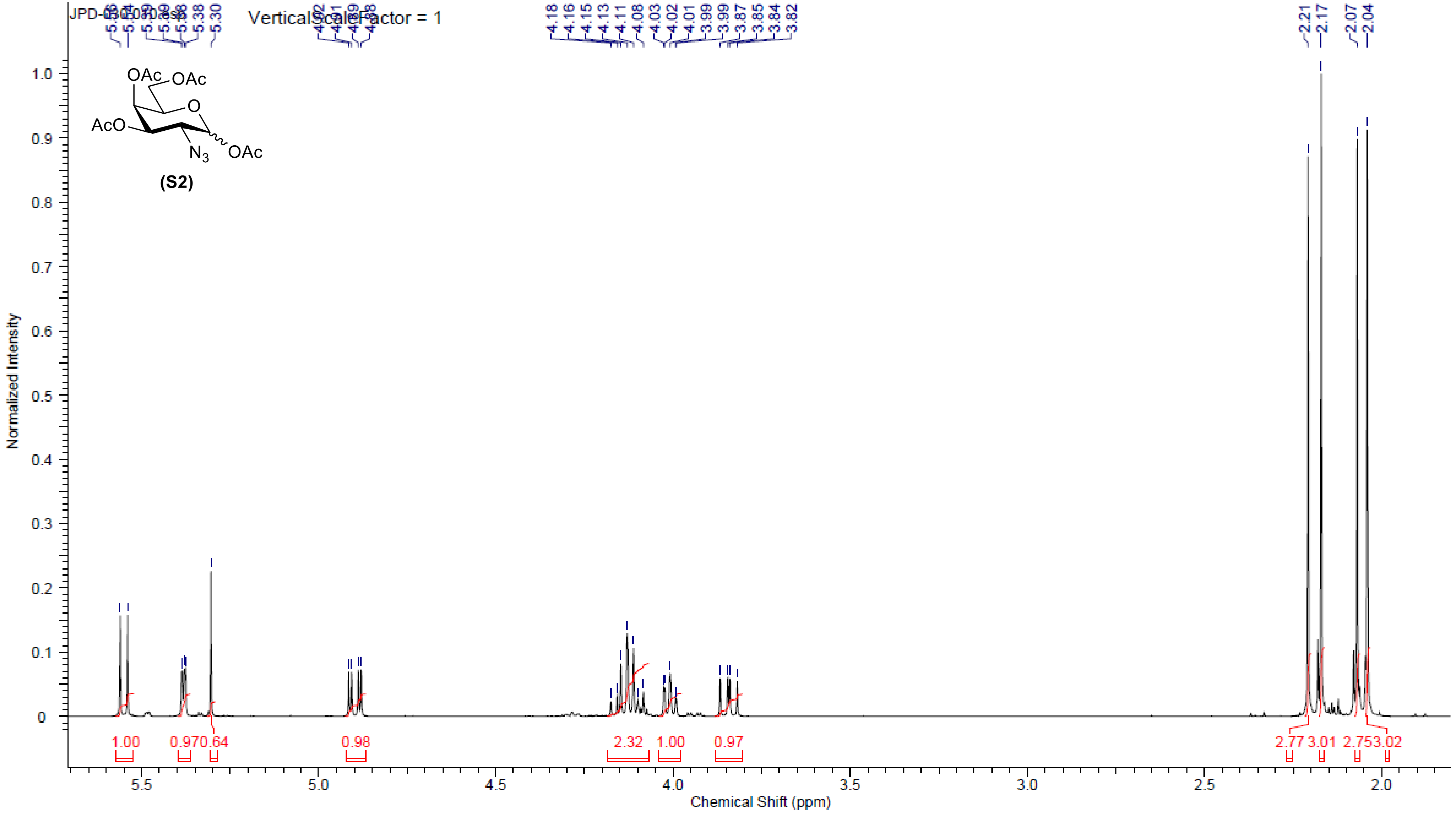

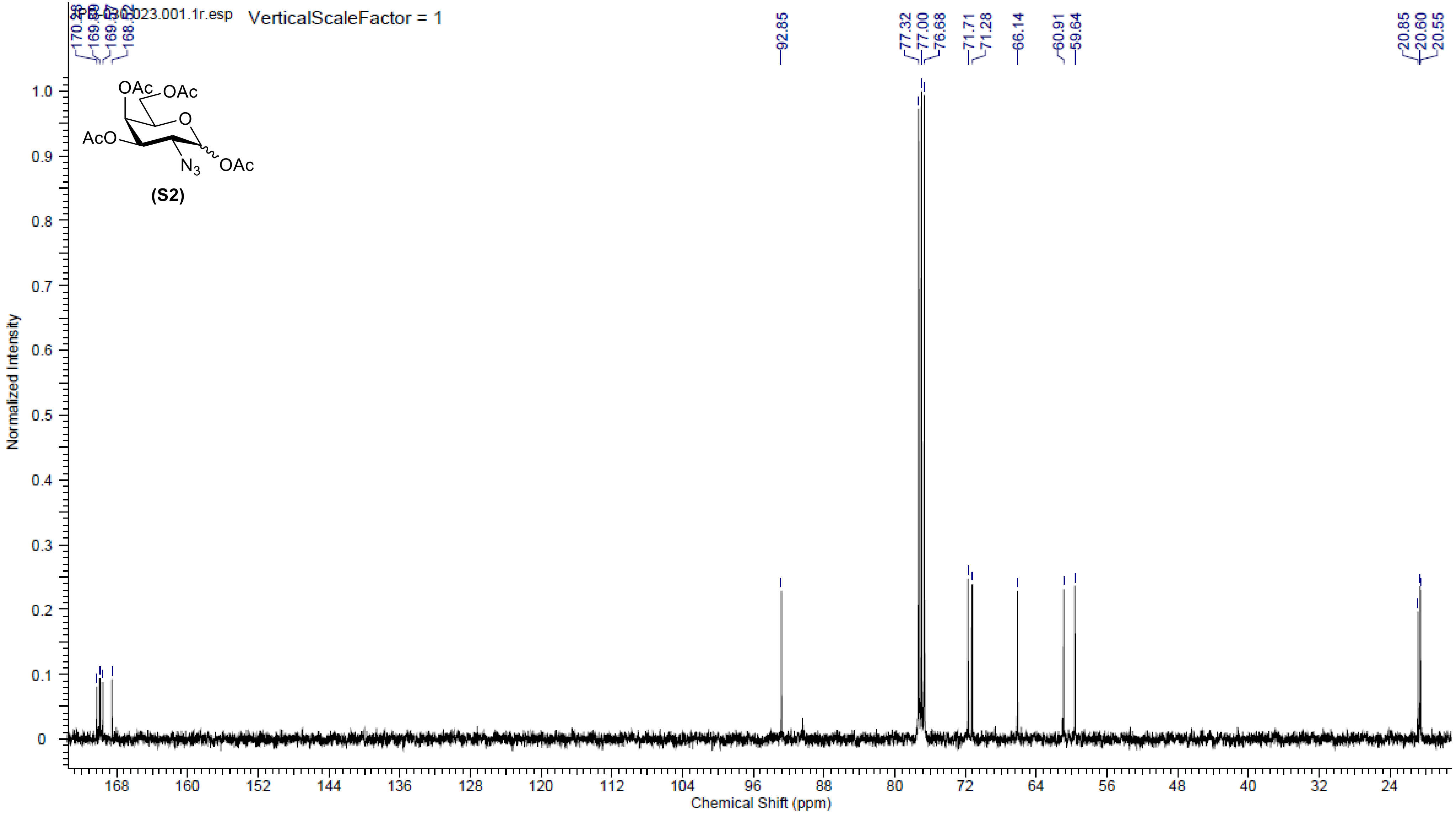
VerticalScaleFactor = 1
92.85
77.32
77.00
76.68
71.71
71.28
66.14
60.91
59.64
20.85
20.60
20.55
OAc
OAc
O
AcO
N3
OAc
(S2)
Normalized Intensity
Chemical Shift (ppm)

*3,4,6-Tri-O-acetyl-2-azido-2-deoxy-α-D-galactopyranosyl trichloroacetimidate (**S3**)*

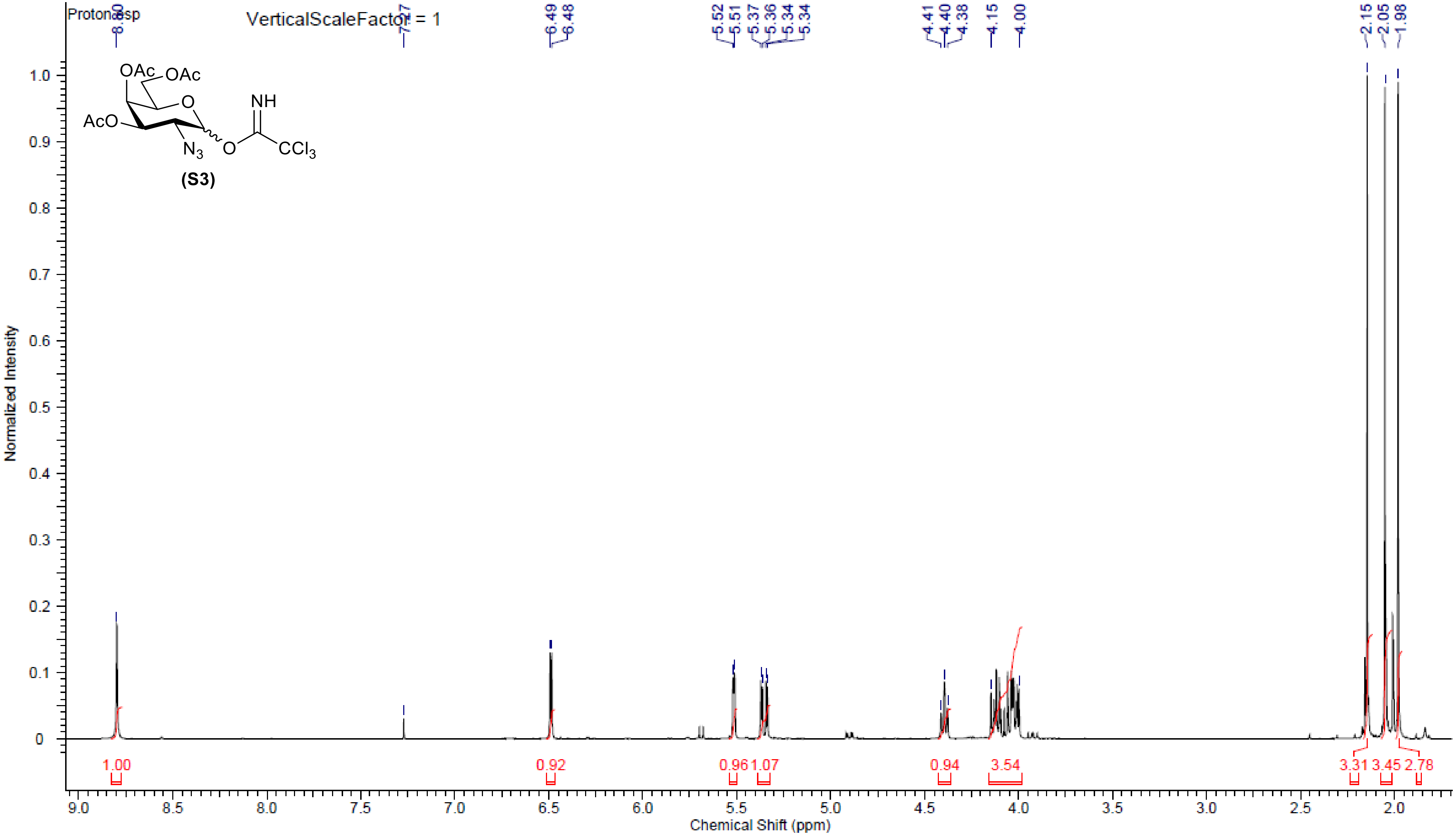

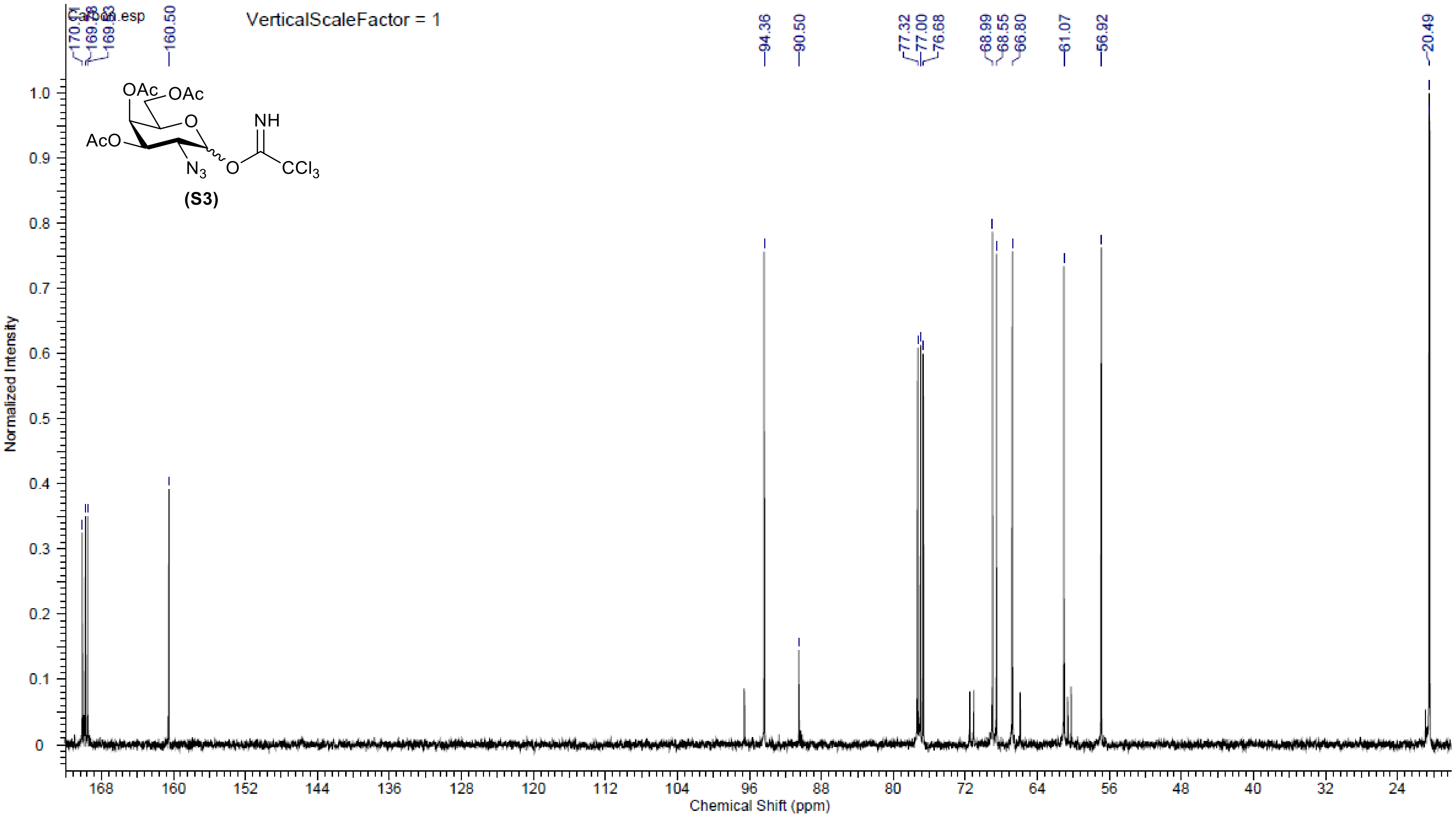
Carbon.esp
VerticalScaleFactor = 1
170.11
169.78
169.63
160.50
94.36
90.50
77.32
77.00
76.68
68.99
68.55
66.80
61.07
56.92
20.49
OAc
OAc
O
NH
AcO
N3
O
CCl3
(S3)
Normalized Intensity
Chemical Shift (ppm)

*N-Fmoc-O-(3,4,6-Tri-O-acetyl-2-azido-2-deoxy-α/β-D-galactopyranosyl)-L-serine tert-butyl ester (**S4i**)*

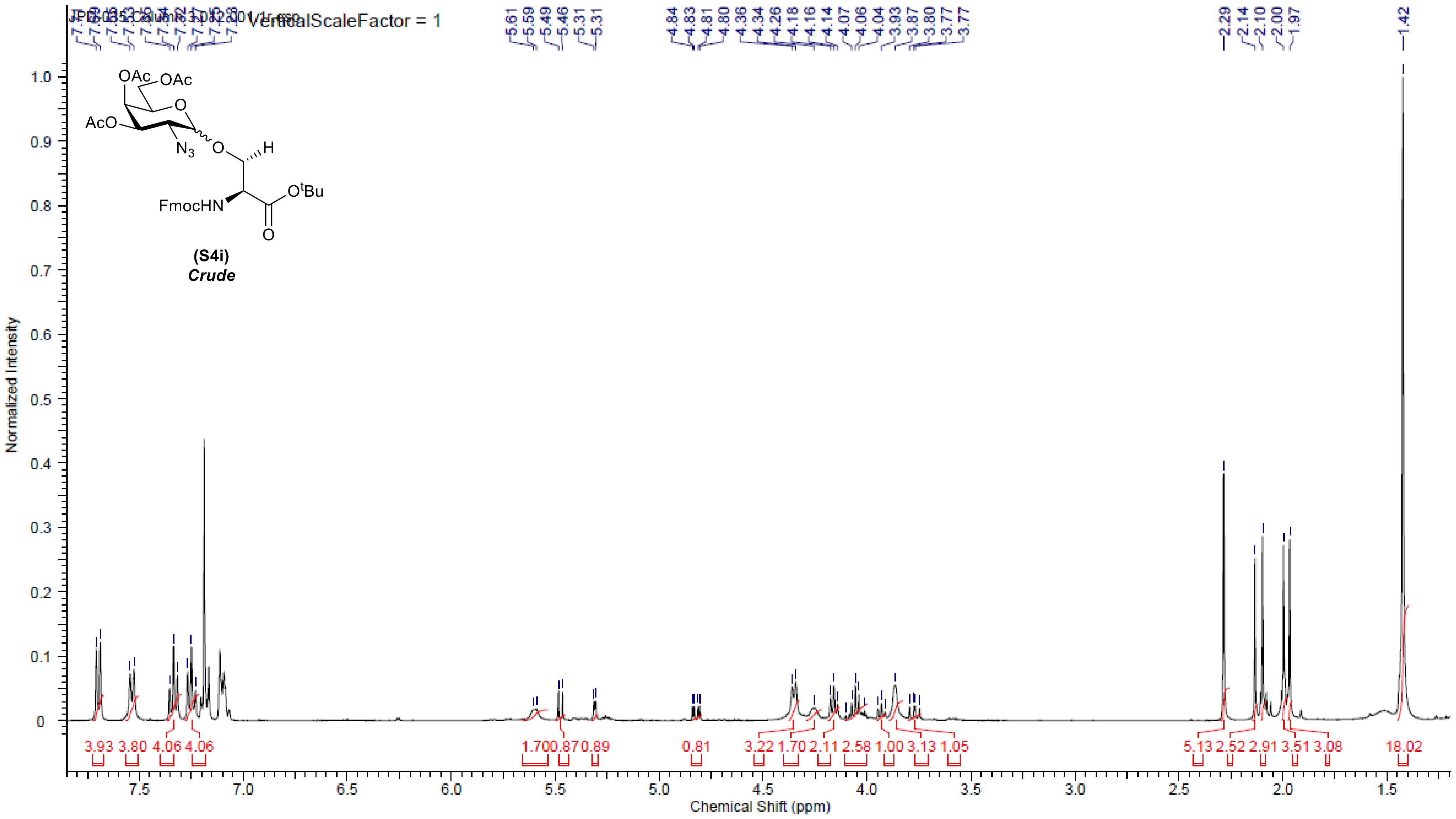

*N-Fmoc-O-(3,4,6-Tri-O-acetyl-2-azido-2-deoxy-α/β-D-galactopyranosyl)-L-threonine tert-butyl ester (**S4ii**)*

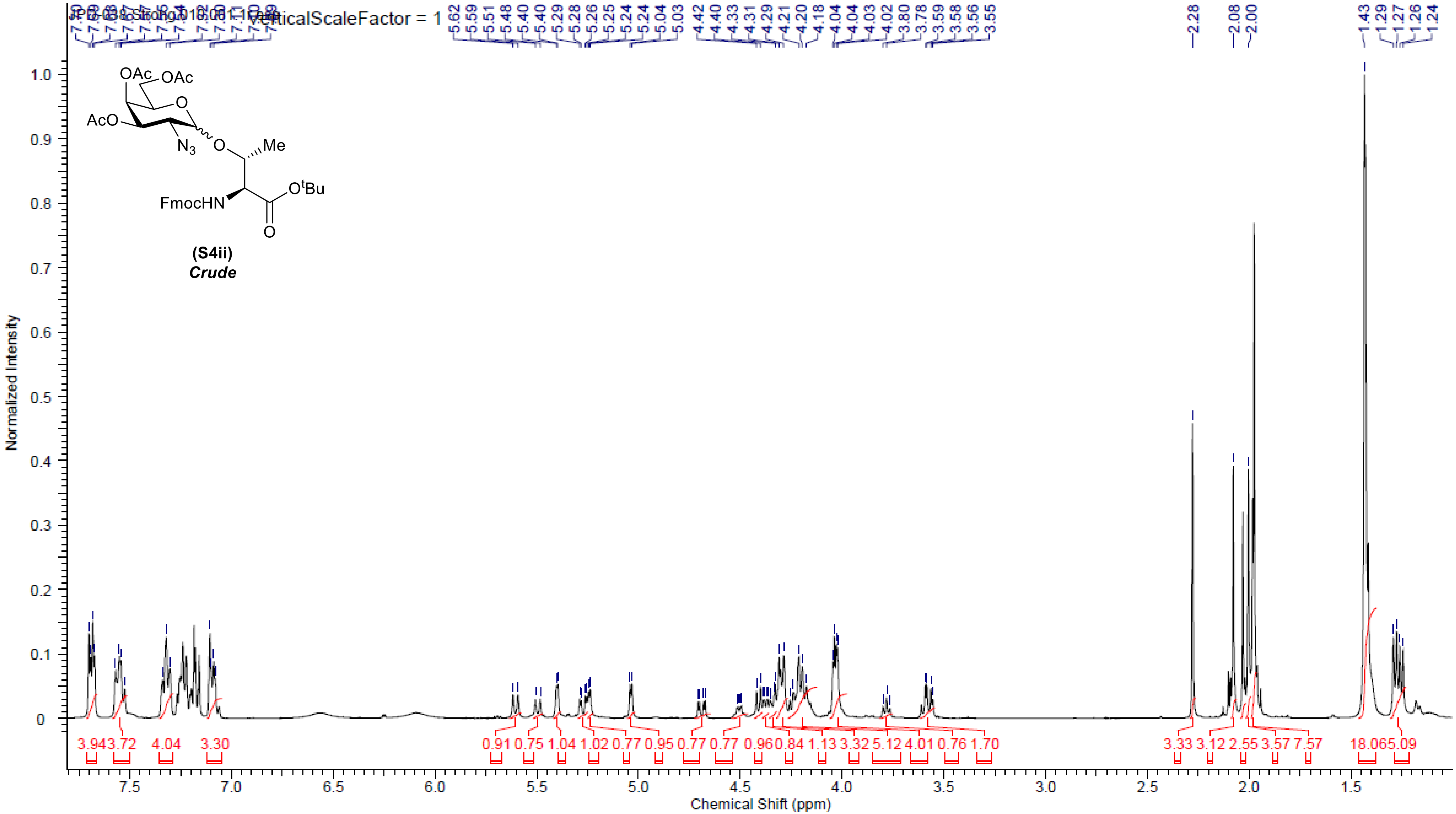

*N-Fmoc-O-(2-acetimido-2-deoxy-3,4,6-Tri-O-acetyl-α-D-galactopyranosyl)-L-serine tert-butyl ester (**S5i**)*

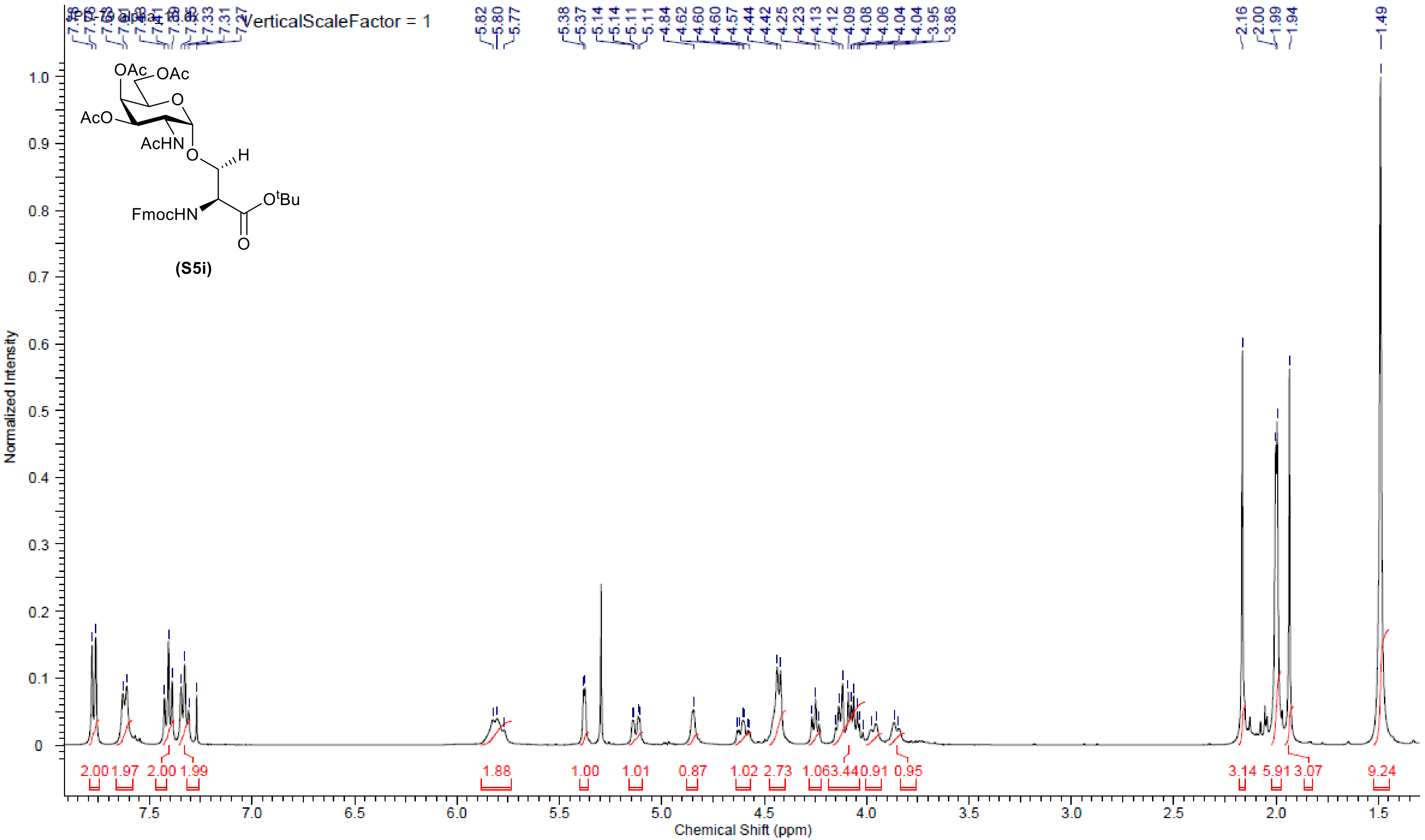

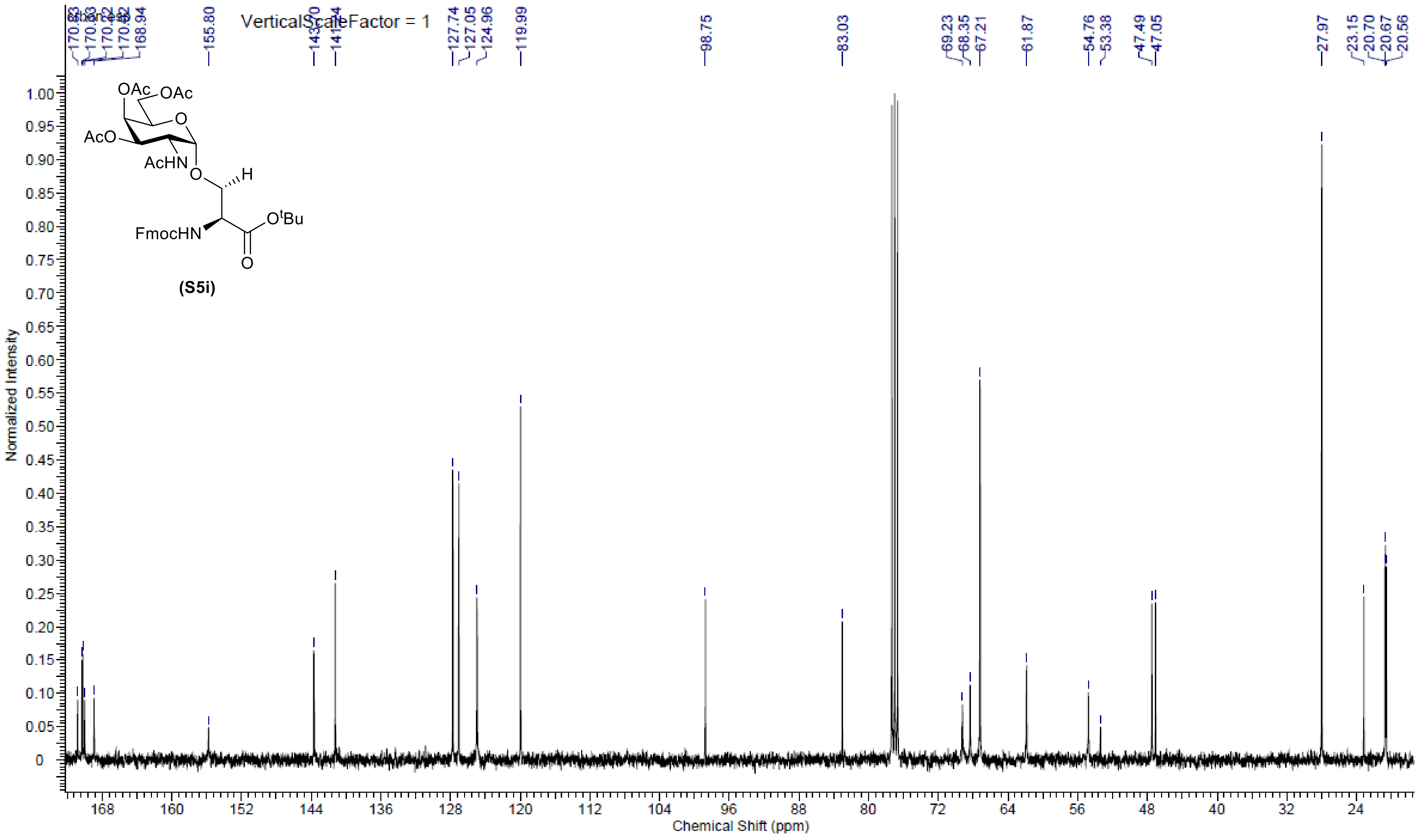
VerticalScaleFactor = 1
Normalized Intensity
Chemical Shift (ppm)
OAc
OAc
AcO
AcHN
O
H
FmocHN
O^tBu
(S5i)
168.94
155.80
127.74
127.05
124.96
119.99
98.75
83.03
69.23
68.35
67.21
61.87
54.76
53.38
47.49
47.05
27.97
23.15
20.70
20.67
20.56

*N-Fmoc-O-(2-acetimido-2-deoxy-3,4,6-Tri-O-acetyl-α-D-galactopyranosyl)-L-threonine tert-butyl ester (**S5ii**)*

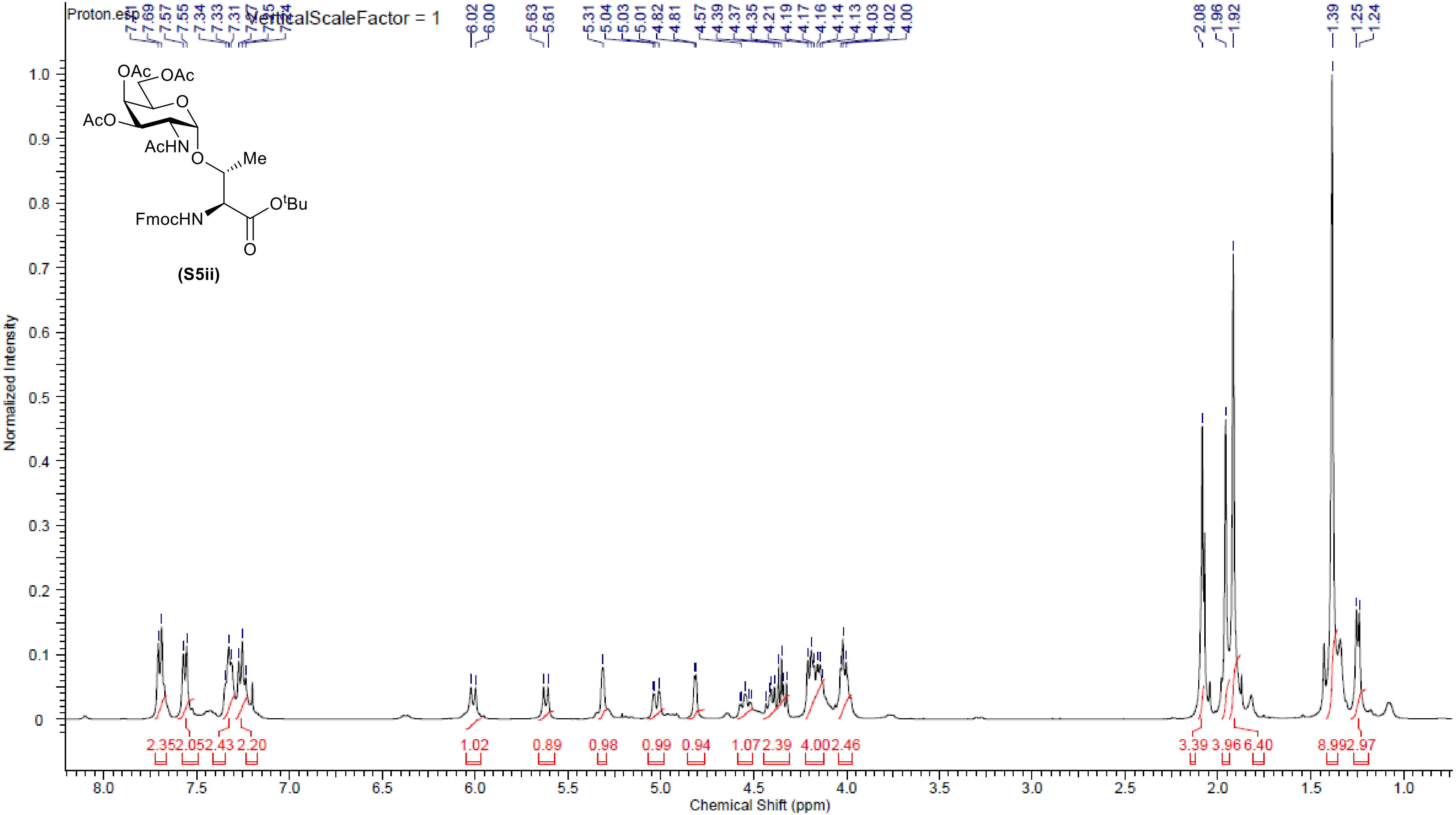

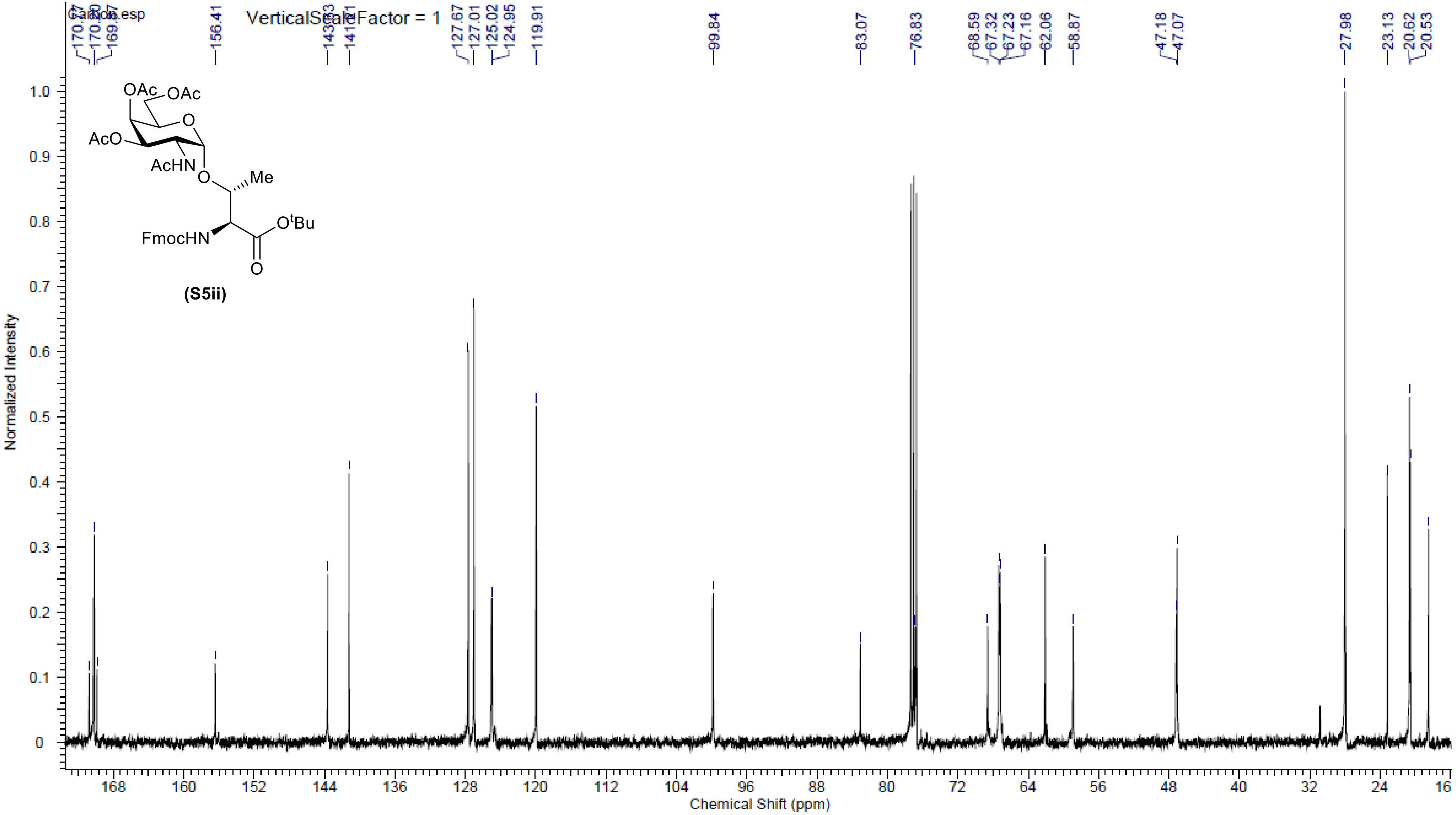

Carbon.esp
VerticalScaleFactor = 1
156.41
127.67
127.01
125.02
124.95
119.91
99.84
83.07
76.83
68.59
67.32
67.23
67.16
62.06
58.87
47.18
47.07
27.98
23.13
20.62
20.53
OAc
OAc
O
AcO
AcHN
O
Me
O^tBu
FmocHN
O
(S5ii)
Normalized Intensity
Chemical Shift (ppm)

*N-Fmoc-O-(2-acetimido-2-deoxy-3,4,6-Tri-O-acetyl-α-D-galactopyranosyl)-L-serine (**S6i**)*

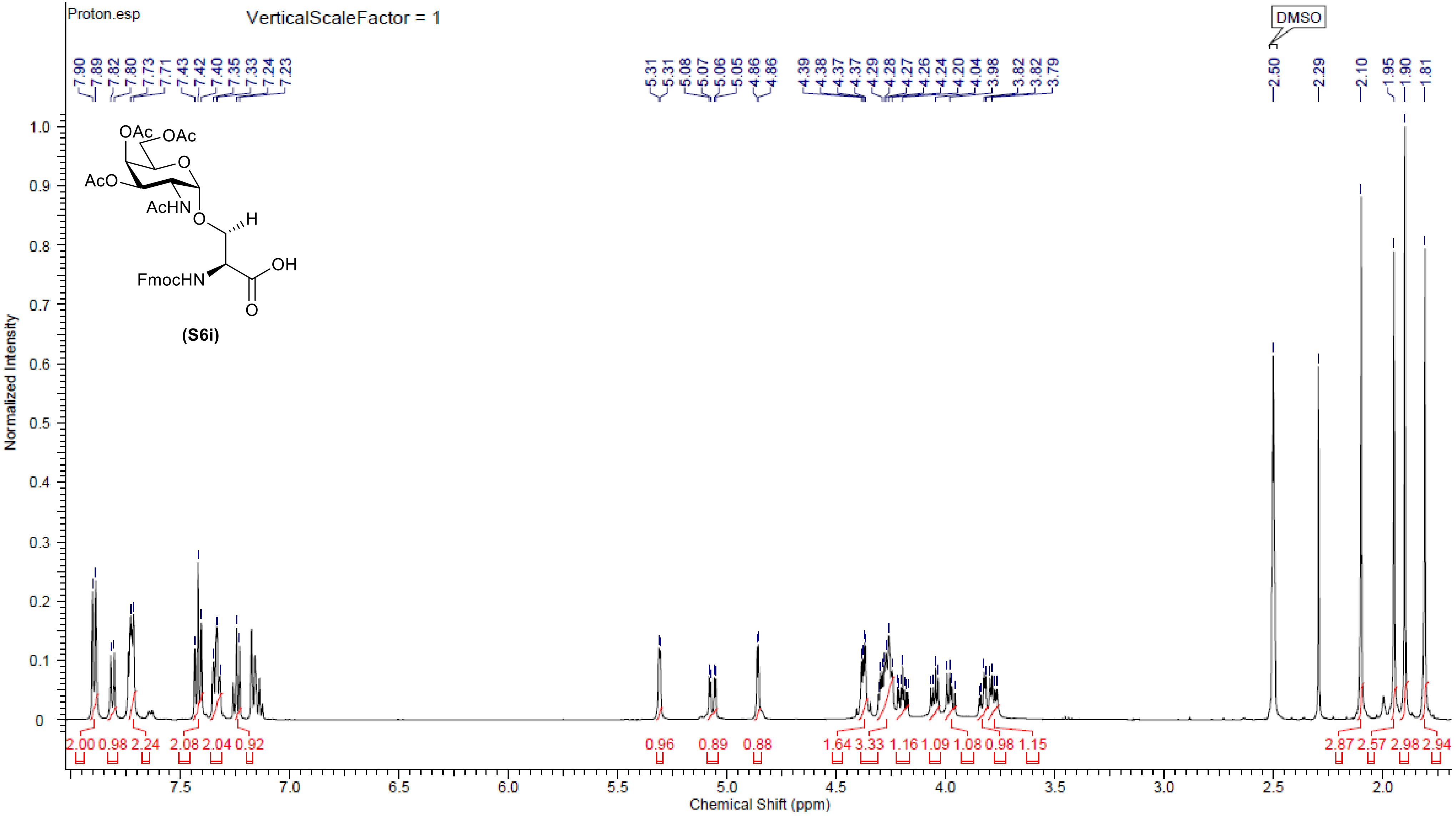

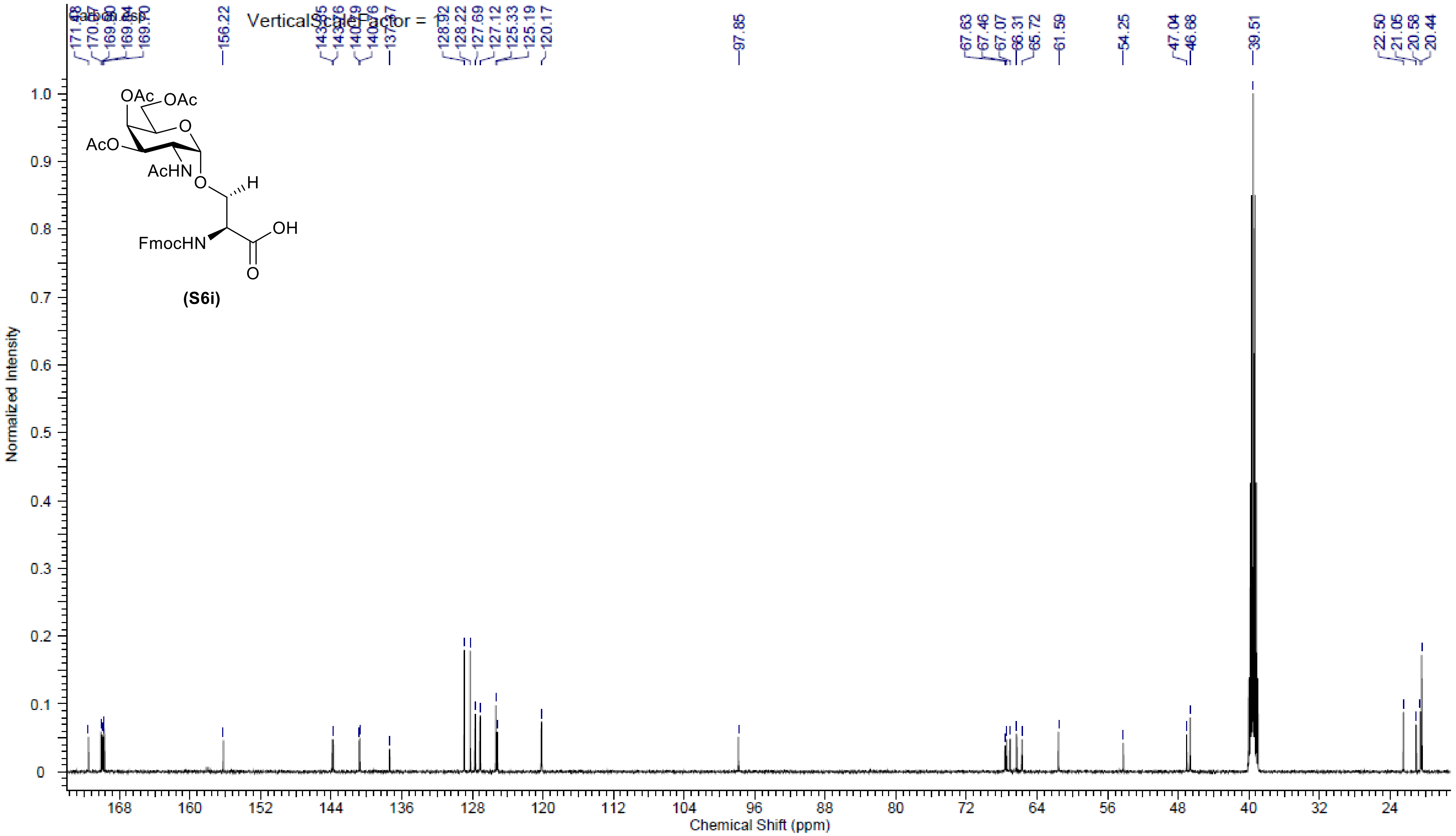
VerticalScaleFactor = 1
156.22
128.92
128.22
127.69
127.12
125.33
125.19
120.17
97.85
67.63
67.46
67.07
65.72
61.59
54.25
47.04
39.51
22.50
21.05
20.58
20.44
OAc
OAc
AcO
O
AcHN
O
H
FmocHN
OH
O
(S6i)
Normalized Intensity
Chemical Shift (ppm)

*N-Fmoc-O-(2-acetimido-2-deoxy-3,4,6-Tri-O-acetyl-α-D-galactopyranosyl)-L-threonine (**S6ii**)*

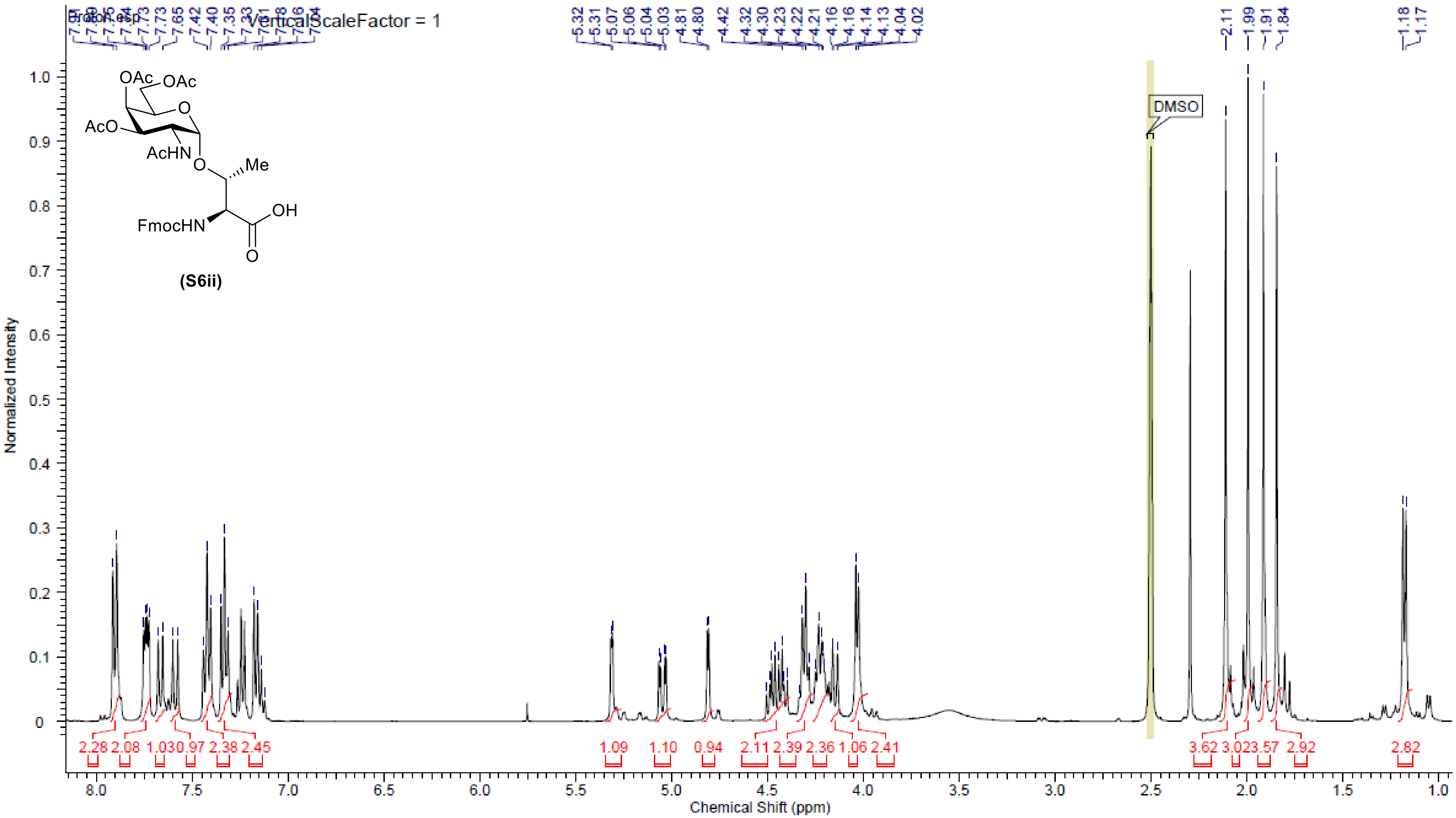
VerticalScaleFactor = 1
5.32 5.31 5.07 5.06 5.04 5.03 4.81 4.80 4.42 4.32 4.30 4.23 4.22 4.21 4.16 4.16 4.14 4.13 4.04 4.02
2.11 1.99 1.91 1.84
1.18 1.17
OAc OAc
AcO
AcHN
O
Me
FmocHN
OH
O
(S6ii)
DMSO
Normalized Intensity
2.28 2.08 1.03 0.97 2.38 2.45 1.09 1.10 0.94 2.11 2.39 2.36 1.06 2.41 3.62 3.02 3.57 2.92 2.82
Chemical Shift (ppm)

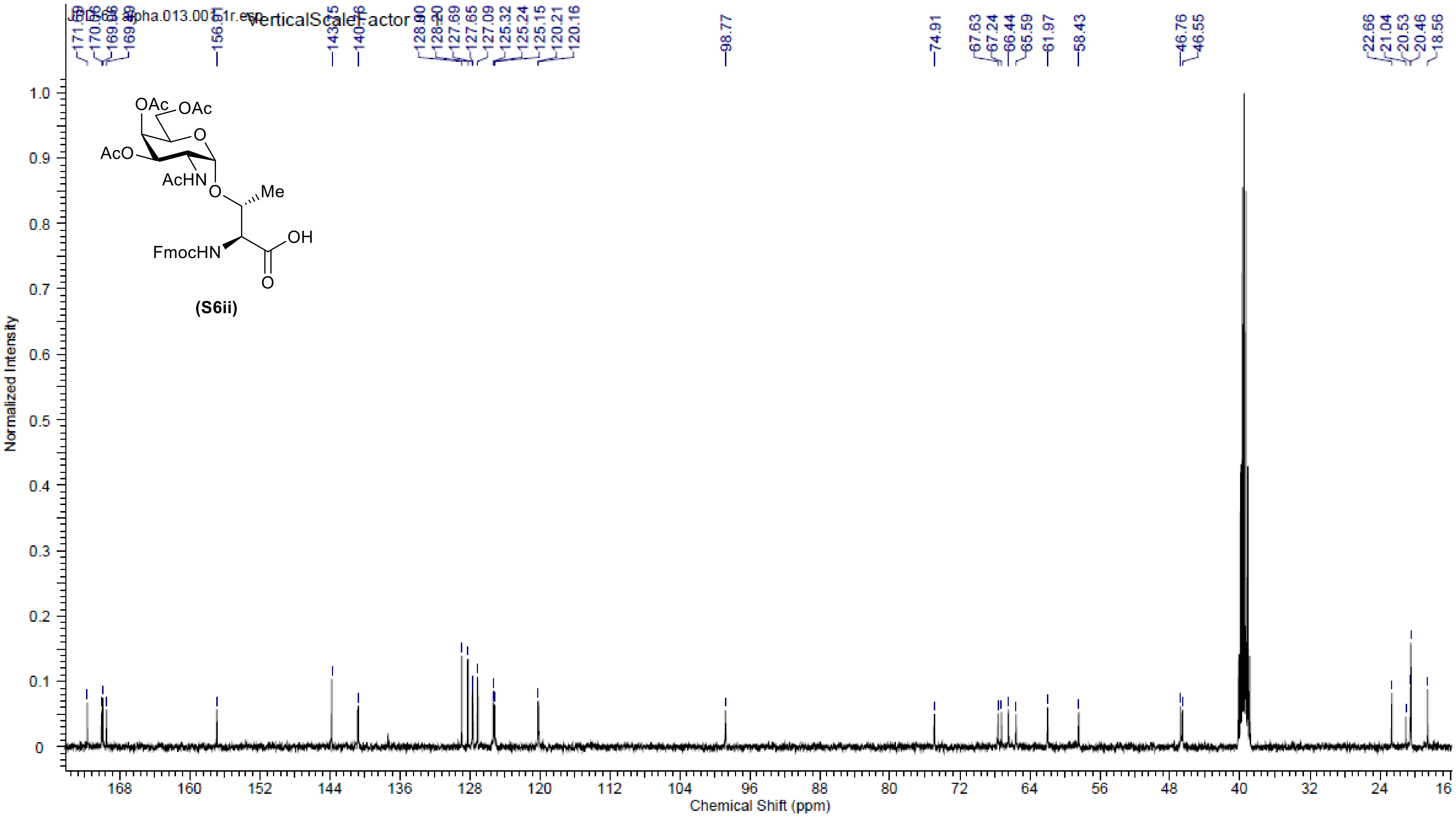

VerticalScaleFactor = 1
127.69
127.65
127.09
125.32
125.24
125.15
120.21
120.16
98.77
74.91
67.63
67.24
66.44
65.59
61.97
58.43
46.76
46.55
22.66
21.04
20.53
20.46
18.56
OAc
OAc
O
AcO
AcHN
O
Me
OH
FmocHN
O
(S6ii)
Normalized Intensity
Chemical Shift (ppm)

HPLC Traces

*H$_2$N-GVAPDTRP-CONH$_2$ (**1**)*

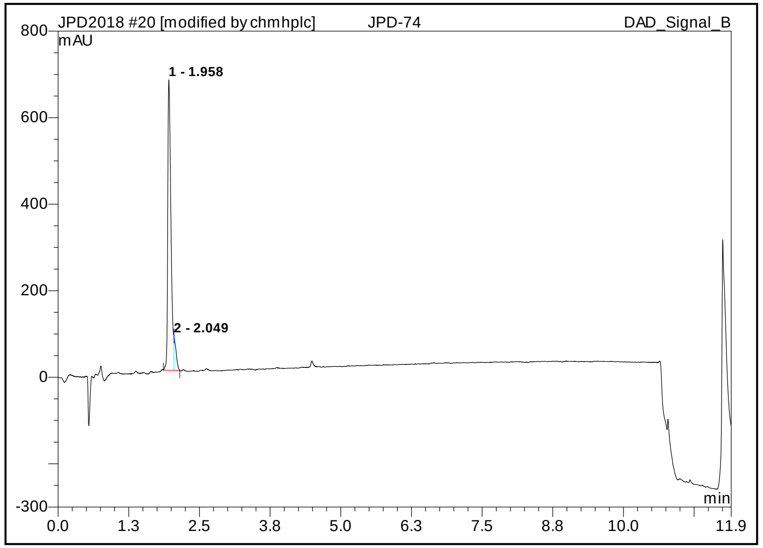

*H$_2$N-GVAPGSTAPPA-CONH$_2$ (**3**)*

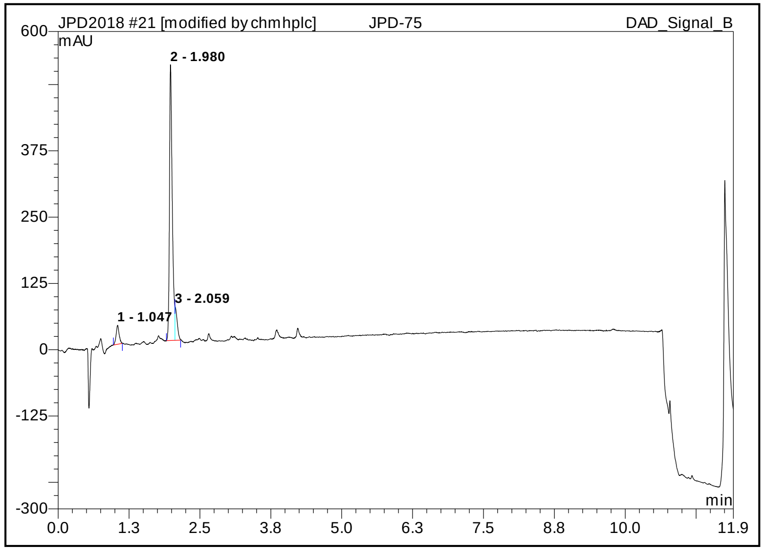

*H$_2$N-GVAPDT(GalNAc)RP-CONH$_2$ (**2**)*

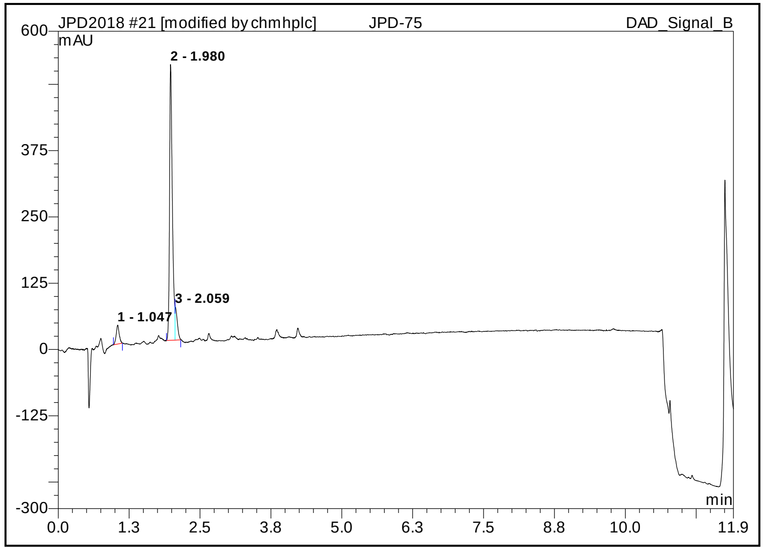

*H$_2$N-GVAPGST(GalNAc)APPA-CONH$_2$ (**4**)*

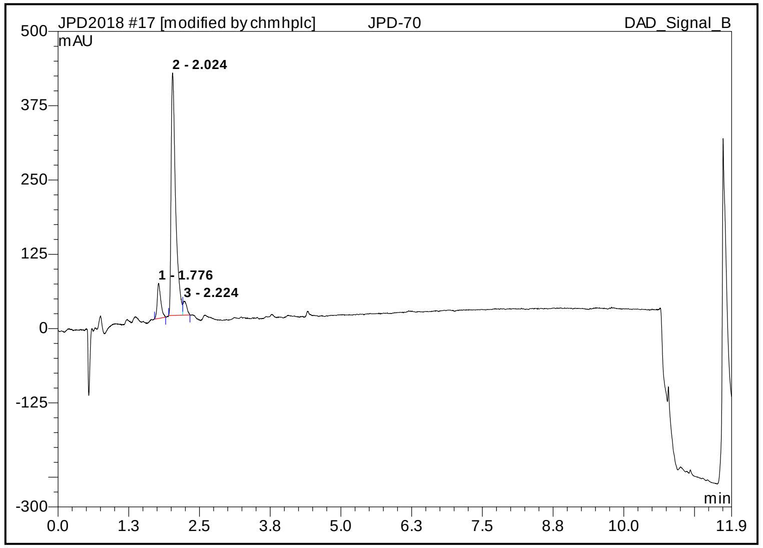

*H_2N-GVAPGS(GalNAc)TAPPA-$CONH_2$ (**5**)*

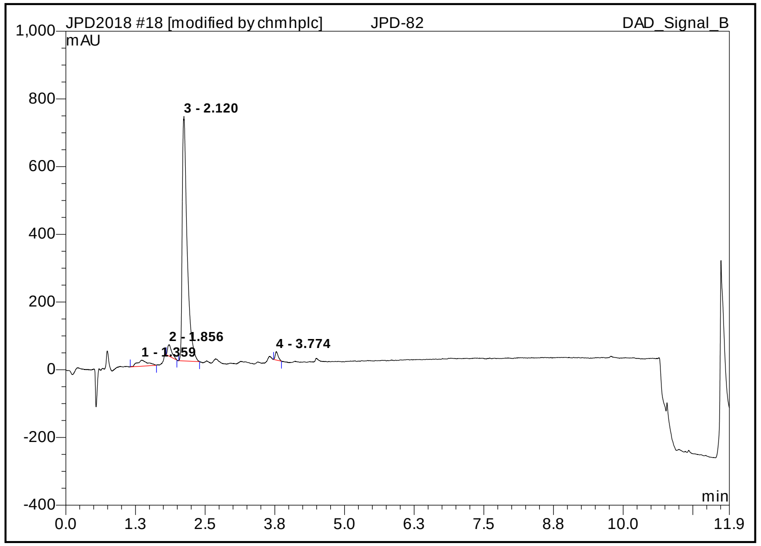

*H_2N-GVAADTRP-$CONH_2$ (**7**)*

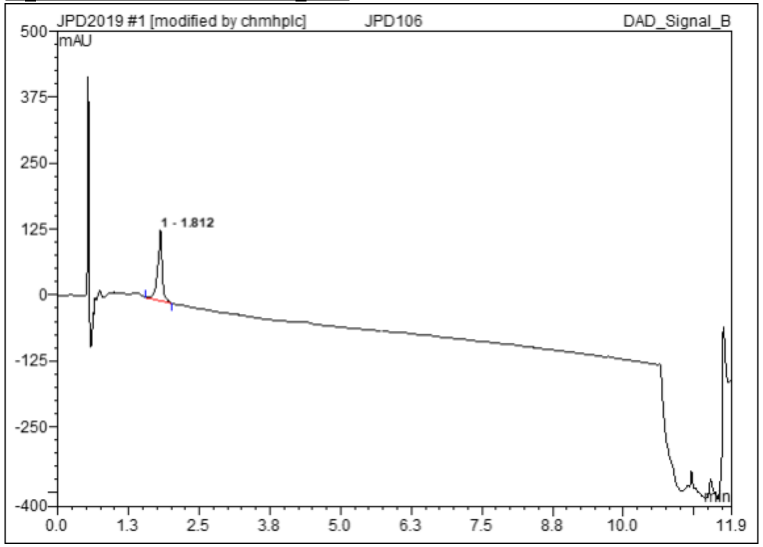

*H_2N-GVAPGS(GalNAc)T(GalNAc)APPA-$CONH_2$ (**6**)*

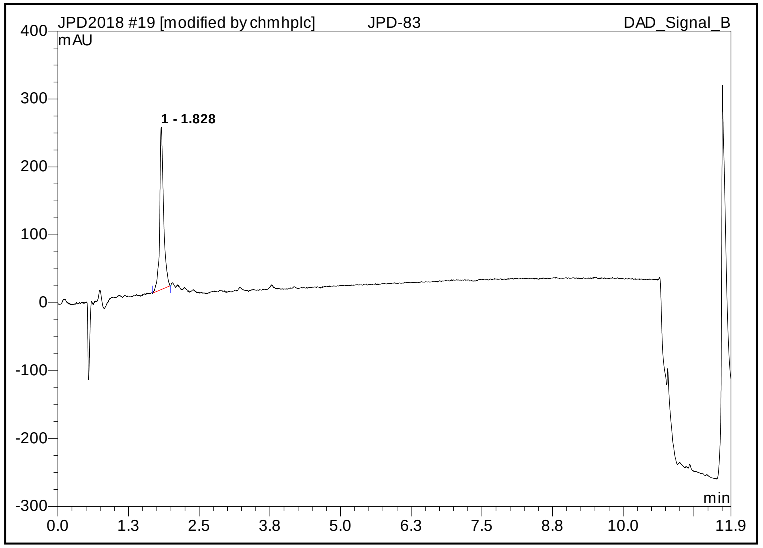

*H_2N-GVAADT(GalNAc)RP-$CONH_2$ (**8**)*

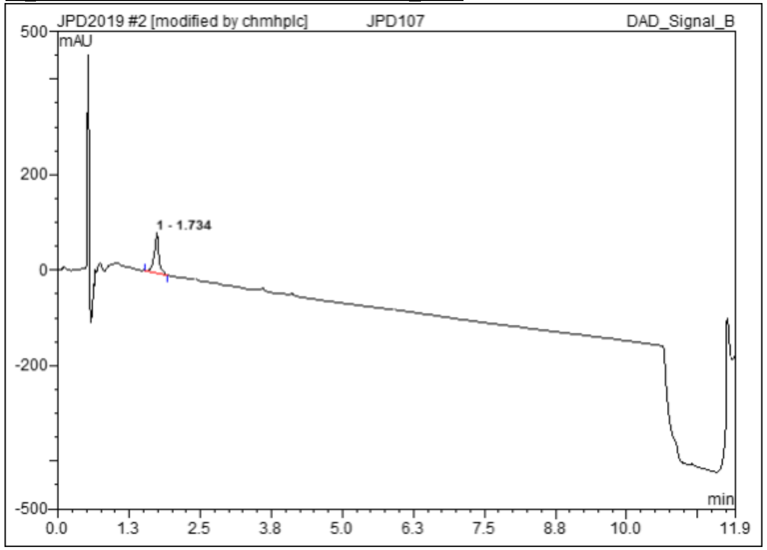

*H_2N-GV(AEEAc)$_2$APDT(GalNAc)RP-CONH$_2$ (**10**)*

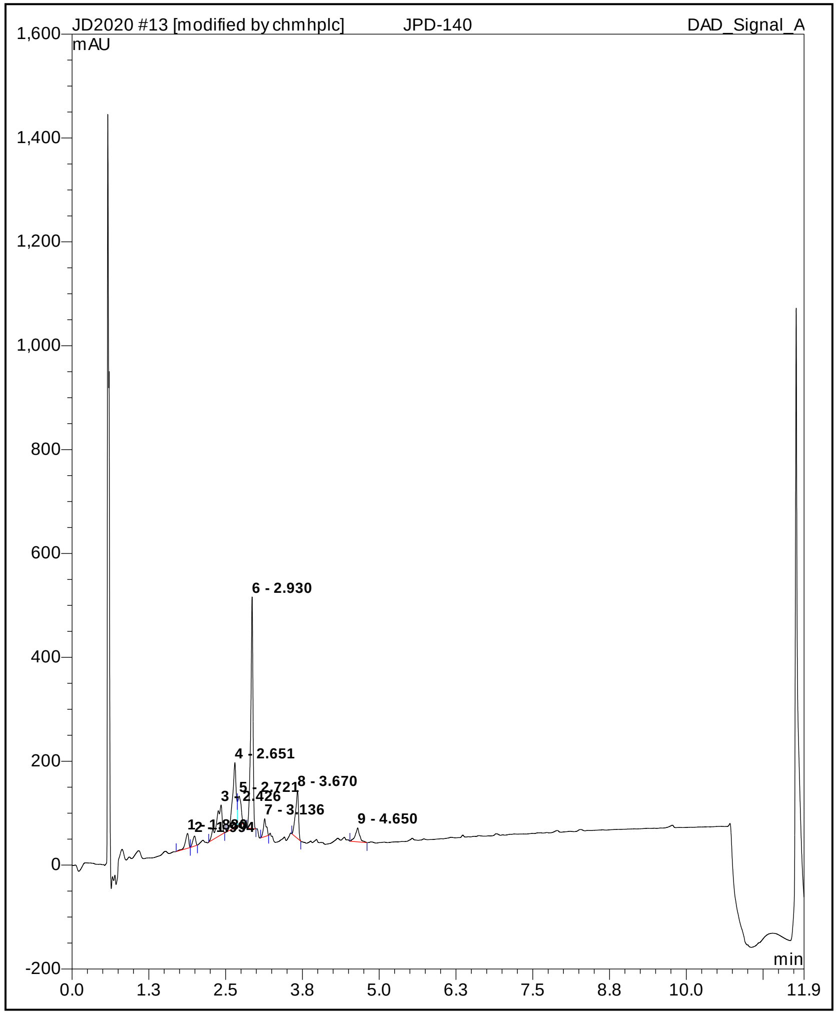

*H_2N-GV(AEEAc)$_2$APGSTAPPA-CONH$_2$ (**13**)*

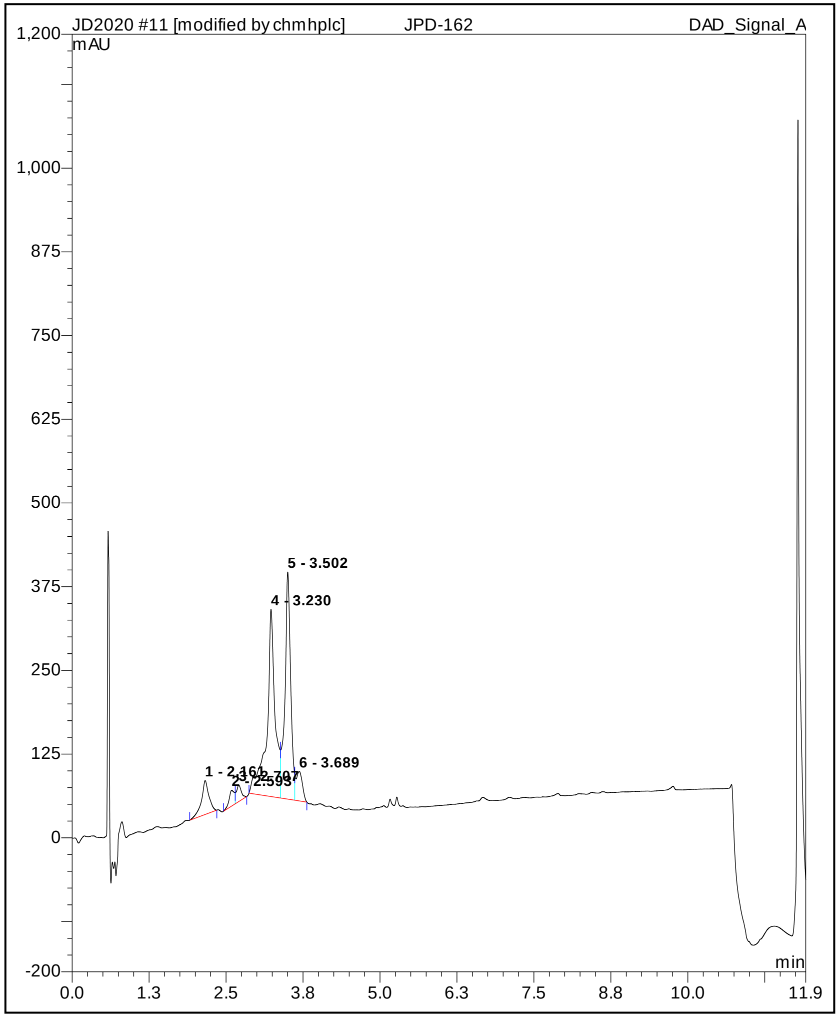

H_2N-GV(AEEAc)$_2$APGST(GalNAc)APPA-$CONH_2$ (**14**)

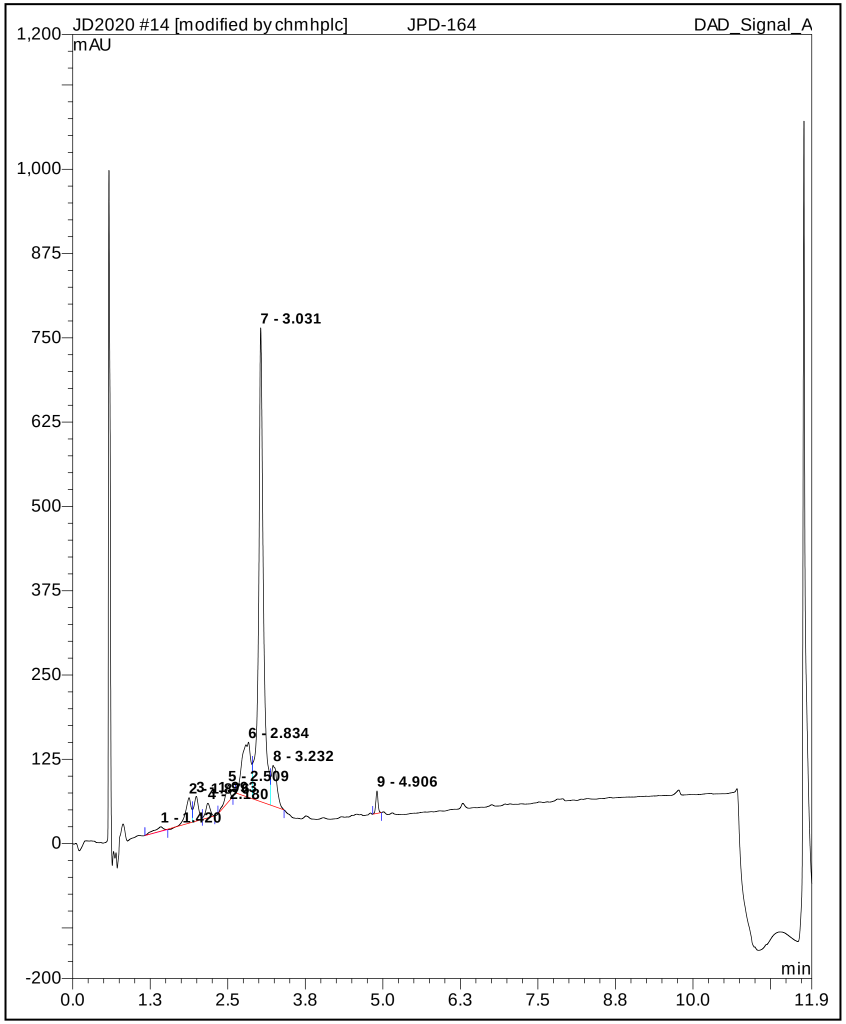

H_2N-GV(AEEAc)$_2$APGS(GalNAc)TAPPA-$CONH_2$ (**15**)

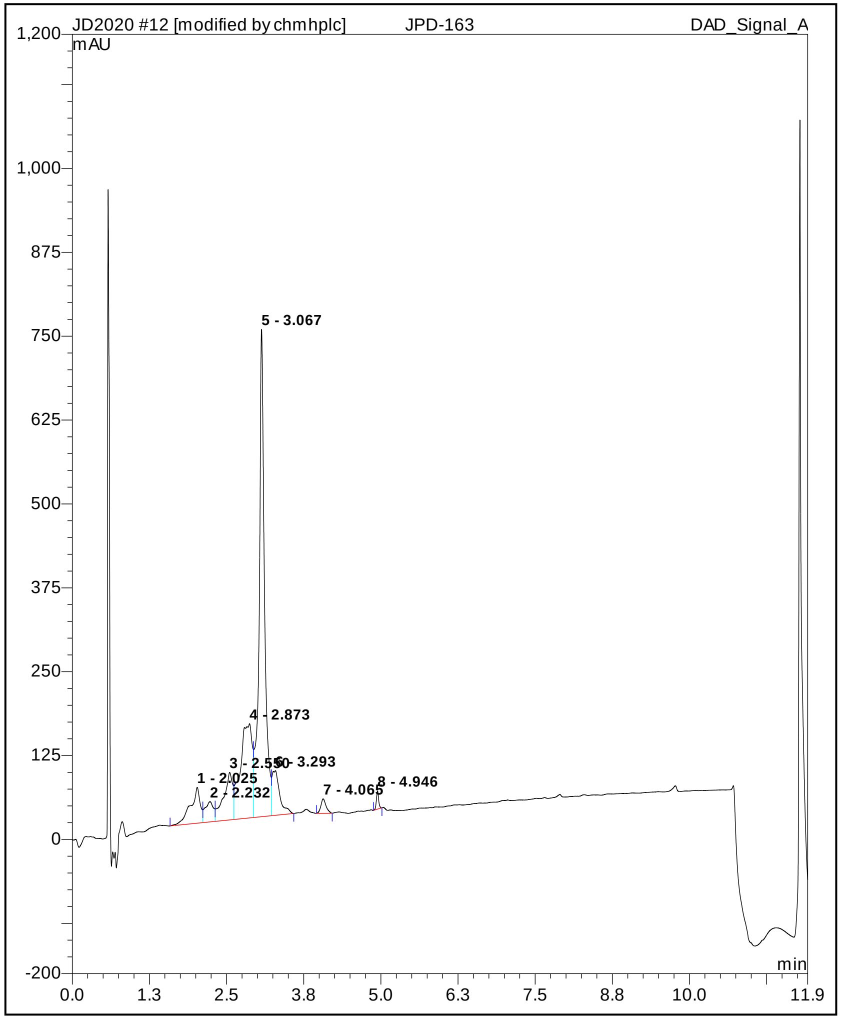

*H_2N-GV(AEEAc)$_2$APGS(GalNAc)T(GalNAc)APPA-CONH$_2$ (**16**)*

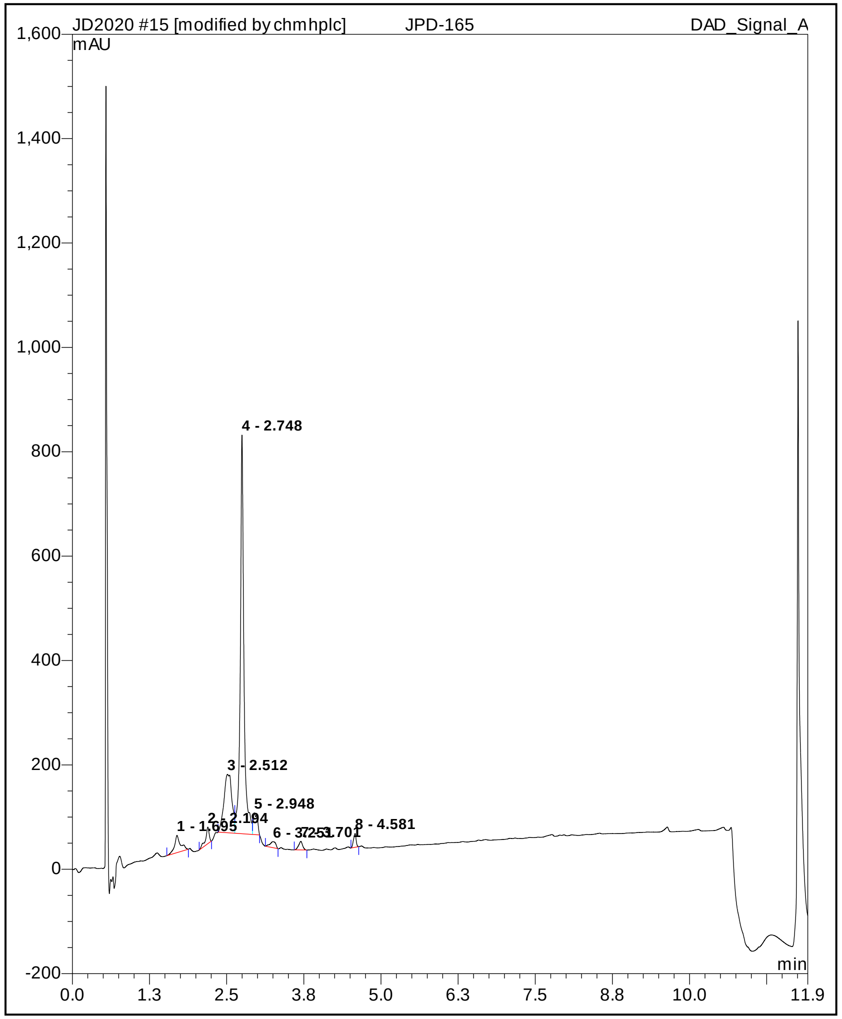

*H_2N-GV(AEEAc)$_2$APPATSGPA-CONH$_2$ (**17**)*

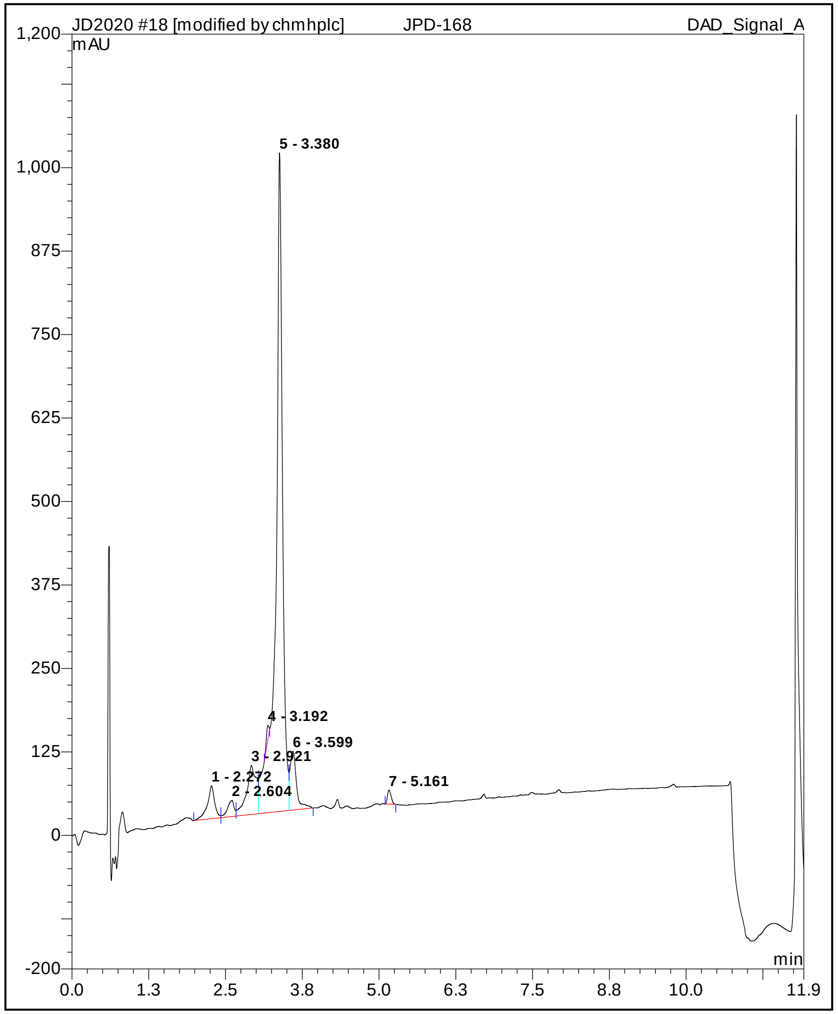

*H_2N-GV(AEEAc)$_2$APDTRP-$CONH_2$ (**18**)*

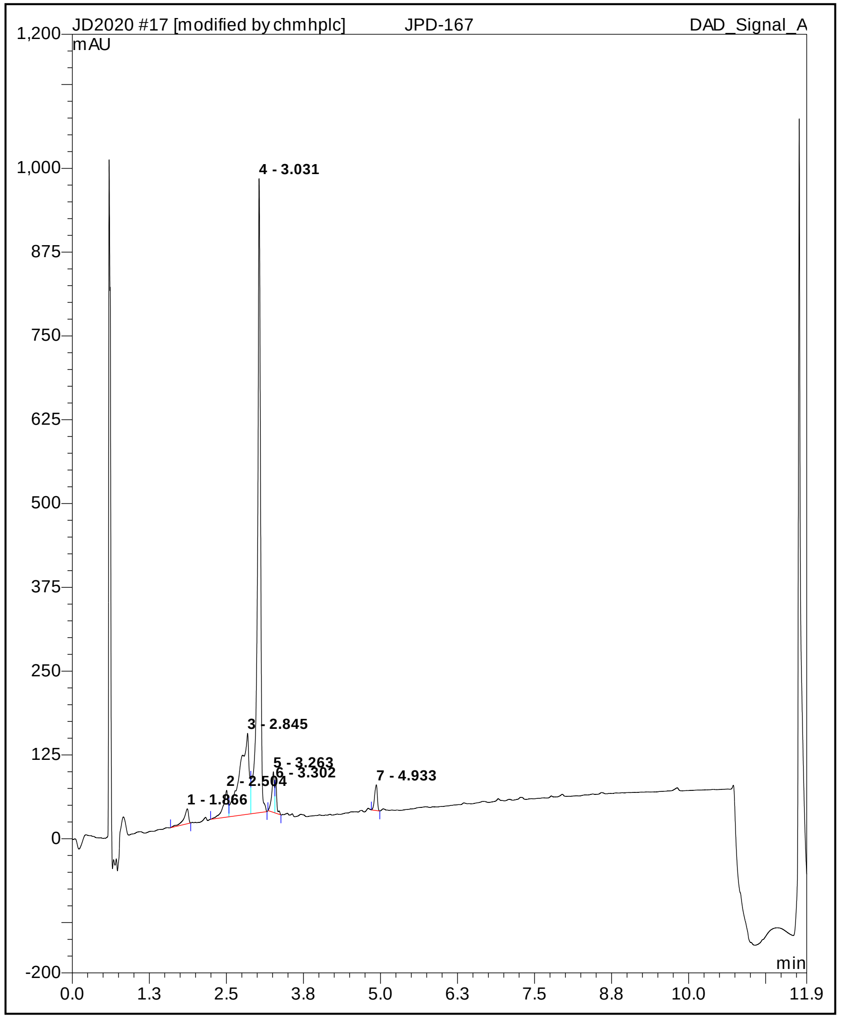

Supplement: Supplementary file 1 [file DataSheet1.pdf]
